# BBX19 fine-tunes the circadian rhythm by interacting with PSEUDO-RESPONSE REGULATOR proteins to facilitate their repressive effect on morning-phased clock genes

**DOI:** 10.1093/plcell/koab133

**Published:** 2021-05-14

**Authors:** Li Yuan, Yingjun Yu, Mingming Liu, Yang Song, Hongmin Li, Junqiu Sun, Qiao Wang, Qiguang Xie, Lei Wang, Xiaodong Xu

**Affiliations:** 1 State Key Laboratory of Crop Stress Adaptation and Improvement, School of Life Sciences, Henan University, Kaifeng 475004, China; 2 Key Laboratory of Plant Molecular Physiology, CAS Center for Excellence in Molecular Plant Sciences, Institute of Botany, Chinese Academy of Sciences, Beijing 100093, China; 3 University of Chinese Academy of Sciences, Beijing 100049, China; 4 College of Life Sciences, Hebei Normal University, Shijiazhuang 050024, China

## Abstract

The core plant circadian oscillator is composed of multiple interlocked transcriptional–translational feedback loops, which synchronize endogenous diel physiological rhythms to the cyclic changes of environmental cues. PSEUDO-RESPONSE REGULATORS (PRRs) have been identified as negative components in the circadian clock, though their underlying molecular mechanisms remain largely unknown. Here, we found that a subfamily of zinc finger transcription factors, B-box (BBX)-containing proteins, have a critical role in fine-tuning circadian rhythm. We demonstrated that overexpressing *Arabidopsis thaliana BBX19* and *BBX18* significantly lengthened the circadian period, while the null mutation of *BBX19* accelerated the circadian speed. Moreover, BBX19 and BBX18, which are expressed during the day, physically interacted with PRR9, PRR7, and PRR5 in the nucleus in precise temporal ordering from dawn to dusk, consistent with the respective protein accumulation pattern of PRRs. Our transcriptomic and genetic analysis indicated that BBX19 and PRR9, PRR7, and PRR5 cooperatively inhibited the expression of morning-phased clock genes. PRR proteins affected BBX19 recruitment to the *CCA1*, *LHY*, and *RVE8* promoters. Collectively, our findings show that BBX19 interacts with PRRs to orchestrate circadian rhythms, and suggest the indispensable role of transcriptional regulators in fine-tuning the circadian clock.

## Teaser

BBX19 functions as a regulator of circadian rhythm by complexing with PRR proteins to enhance their repressive effect on *CCA1* transcription.

## Introduction

The circadian clock is a timekeeping mechanism synchronizing self-sustained physiological rhythms to the 24-h environmental cycles. In land plants, the clock is composed of multiple interconnected transcriptional feedback loops (Creux and Harmer, 2019; McClung, 2019), in which sequentially expressed circadian core components allow plants to predict daily changes of zeitgebers by fine-tuning circadian parameters of the rhythmic expression of their target genes. In the *Arabidopsis thaliana* circadian clock, the morning loop consists of two MYB-like transcription factors CIRCADIAN CLOCK ASSOCIATED (CCA1) and LATE ELONGATED HYPOCOTYL (LHY), and their homologs, REVEILLE8 (RVE8/LHY-CCA1-LIKE5/LCL5) and RVE4, as well as PSEUDO-RESPONSE REGULATOR (PRR7 and PRR9). CCA1 and LHY inhibit transcription of evening-phased *PRR5*, *TOC1*/*PRR1*, and *LUX ARRHYTHMO* (*LUX*; Lau et al., 2011; Nagel et al., 2015; Kamioka et al., 2016). In contrast, RVE8 and RVE4 dynamically interact with transcriptional coactivators, NIGHT LIGHT-INDUCIBLE AND CLOCK-REGULATED1 (LNK1) and LNK2, in the morning to positively regulate expression of *PRR5* and *TOC1* (Rugnone et al., 2013; Xie et al., 2014). In turn, PRRs function as transcriptional repressors of morning-phased clock genes (Nakamichi et al., 2005, 2010; Gendron et al., 2012; Huang et al., 2012). TOC1 interacts with TCP transcription factor CCA1 HIKING EXPEDITION (CHE) to prevent the activation of *CCA1* at night (Pruneda-Paz et al., 2009). PRR5, PRR7, and PRR9 interact with the plant Groucho/TUP1 corepressor, TOPLESS (TPL), and binds to the *CCA1* promoter to inhibit its expression, thereby regulating the circadian period length (Wang et al., 2013). This highly wired transcriptional network ensures the stability and robustness of the circadian clock.

The B-box zinc-finger subfamily BBX IV in Arabidopsis consists of eight members, BBX18–BBX25, all of which have two B-box motifs in their N terminus but lack one C-terminal CONSTANS, CONSTANS-like (CO-like), and TOC1 (CCT) domain (Khanna et al., 2009). BBX18 and BBX23 are critical for thermomorphogenesis, as they interact with EARLY FLOWERING3 (ELF3) to regulate the PIF4-dependent gene expression and participate in modulating hypocotyl elongation under warm temperature conditions (Ding et al., 2018). ELF3 is required for the formation of the ELF3–ELF4–LUX evening complex (EC) of the circadian clock, and functions in clock gating, photoperiod sensing, and hypocotyl growth (Covington et al., 2001; Liu et al., 2001; Yu et al., 2008; Nusinow et al., 2011; Chow et al., 2012; Anwer et al., 2020). BBX19 overexpression caused photoperiodic late flowering, in which BBX19 interacted with CONSTANS to inhibit the transcription of *FLOWERING LOCUS T* (Wang et al., 2014). BBX19 was therefore considered to function in clock output pathways. In addition, ELF3 is recruited by BBX19 and then degraded by COP1 to regulate the formation of EC, which inhibits *PIF4* and *PIF5* expression, thus promoting evening hypocotyl growth (Wang et al., 2015).

Recently, BBX IV components were reported to modulate photomorphogenesis via their DNA binding ability. BBX21 (STH2) is required for anthocyanin accumulation through direct binding to the *HY5* promoter under light conditions (Datta et al., 2006, 2007; Xu et al., 2016, 2018). BBX21 binds to *MYB12* and *F3H* promoter regions, and this process depends on HY5 (Bursch et al., 2020). HY5 is also required for the binding of BBX20 and BBX23 to the promoter regions of target genes (Zhang et al., 2017; Bursch et al., 2020). BBX21, BBX22 (LZF1/STH3), BBX24 (STO), and BBX25 physically interacts with COP1, suggesting that light signaling regulates BBX proteins via COP1-mediated ubiquitination and proteasomal degradation (Datta et al., 2008; Jiang et al., 2012; Xu et al., 2016; Song et al., 2020). BBX24 and BBX25 interact with HY5, potentially to form inactive heterodimers, direct inhibiting binding of HY5 to the *BBX22* promoter during early seedling development (Gangappa et al., 2013).

BBX20 (DBB2/BZS1) is regulated by light and COP1-mediated ubiquitination, and acts as negative regulator in brassinosteroid pathway to mediate crosstalk between hormone and light signaling (Kumagai et al., 2008; Khanna et al., 2009; Fan et al., 2012; Wei et al., 2016). In addition, *BBX32*, a member of the BBX V family, is regulated by the circadian clock, and its overexpression resulted in a lengthened period of circadian rhythm and late flowering (Tripathi et al., 2017). The ectopic expression of Arabidopsis *BBX32* in soybeans affects the transcription pattern of soybean clock genes, thereby increasing grain yield (Preuss et al., 2012). However, due to the divergence of BBX family functions, the roles of BBX family members in plant growth, and especially in the circadian system are largely unknown.

In this study, we found that BBX19 and BBX18 proteins dynamically interact with PRR9, PRR7, and PRR5 from the early morning onward in the nucleus to regulate circadian periodicity. Temporal transcriptome and genetic analysis showed that BBX19 and PRR9, PRR7, and PRR5 jointly repressed the expression of morning-phased clock genes *CCA1*, *LHY*, and *RVE8*. BBX19 interacted with PRR9 and PRR7 to bind to *CCA1*, *LHY*, and *RVE8* promoters to modulate their transcription. These findings demonstrated that BBX19–PRRs complexes function directly in transcriptional regulation of the circadian clock, further bridging the feedback inhibition of morning circadian genes by sequentially expressed PRRs.

## Results

### Mutation of *BBX19* shortens the circadian period

**Figure 1:**
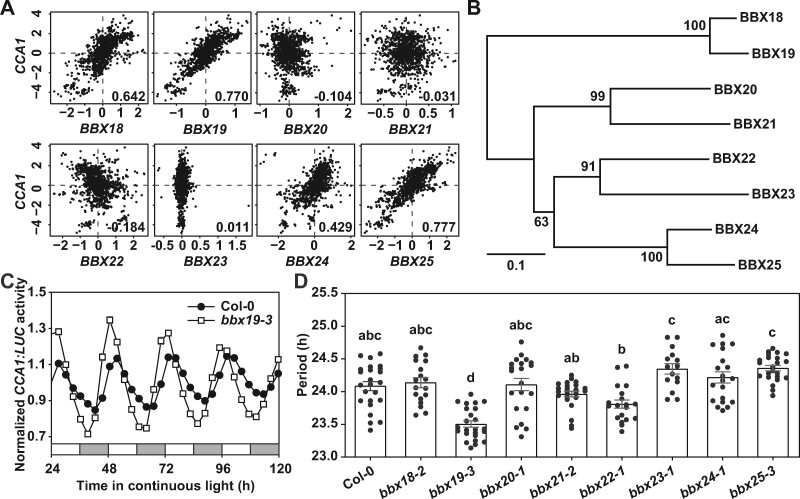
Dysfunction of *BBX19* leads to the accelerated circadian pace. A, Estimation of correlation between *CCA1* and *BBX* subfamily IV genes in co-expression analysis using the multiple microarray- and RNAseq-based coexpression data sets in ATTED-II (http://atted.jp/top_draw.shtml#CoexViewer). The Pearson’s correlation coefficient (*r*-value) was listed in the lower right corner of each panel, which is used to represent the linear association between *CCA1* and *BBX* subfamily genes. The *r*-value of 0 indicates that there is no association, while values of −1 or +1 indicates that there is a strongest linear correlation. B, The phylogenetic radiant tree of eight full-length orthologs of BBX subfamily IV in Arabidopsis. The evolutionary distance was inferred using the neighbor-joining method, and phylogenetic tree was constructed using the Jukes–Cantor genetic distance model in Geneious Tree Builder. C, D, Circadian rhythms of *CCA1:LUC* in the *bbx18-bbx25* mutants were monitored under free-running conditions. Data showing mean ± se for three independent experiments. At least 15 individual seedlings were used for each analysis. Open bars indicate subjective day, and gray bars indicate subjective night (C). Dots indicate individual samples and bars mean period ± se (D). Multiple groups were analyzed with one-way ANOVA followed by Tukey’s multiple comparison test, *P* < 0.05

To expand the known molecular architecture of the circadian clock, we checked multiple microarray- and RNAseq-based coexpression data sets in ATTED-II (http://atted.jp) and retained the top 50 genes highly co-expressed with *CCA1* or *LHY*. After alignment, 32 genes associated with both *CCA1* and *LHY* were obtained (Supplemental Table S1), including *RVE8* and *RVE4*, which encode MYB-like transcription factors similar to CCA1 and LHY (Farinas and Mas, 2011; Rawat et al., 2011). LNK2, LNK3, and LNK4 also showed high correlation coefficients, and LNK2 was reported to physically interact with CCA1, LHY, RVE4, and RVE8 (Xie et al., 2014). In addition, five genes *BBX18*, *BBX19*, *BBX25*, *COL1*, and *COL2* were identified that belong to the functionally diverse BBX family (Figure 1, A and B; Supplemental Table S1 and Supplemental Data Set S1). Overexpression of *COL1* and *COL2* (belonging to BBX structural group I) accelerates the circadian clock, thereby generating a shortened circadian rhythm (Ledger et al., 2001), but no further studies have revealed how they are involved in the circadian clock.

**Figure 2:**
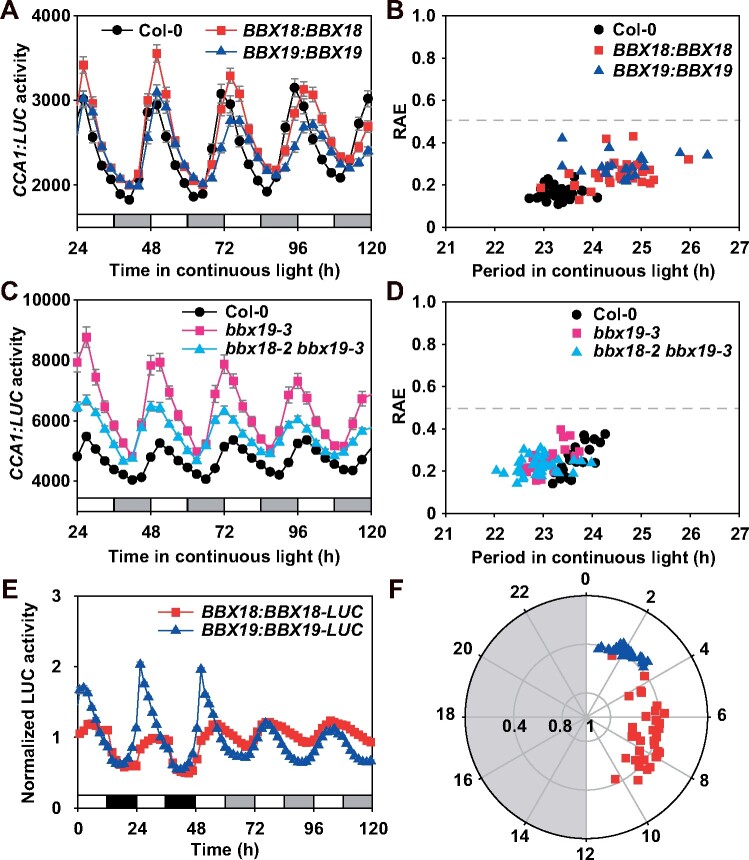
Morning-phased *BBX19* and *BBX18* are involved in regulating self-sustained circadian period. A, B, Increased expression of *BBX18* or *BBX19* lengthened the circadian period length. The full-length gene constructs of *BBX18:BBX18* and *BBX19:BBX19* were transformed into the wild-type plants to generate the overexpression transgenic lines. Period estimation for of individual *CCA1:LUC* rhythm (A) is plotted against their relative amplitude errors (RAEs) (B). RAE is used to define the limit of rhythmicity, a complete sine-fitting wave is defined as 0, and a value of 1 defines the weakest rhythm. Data represent mean ± se from three independent experiments. At least 24 individual seedlings were used for each analysis. Open bars indicate subjective day, and gray bars indicate subjective night. C, D, Circadian rhythm (C) and period estimate (D) of the *bbx18 bbx19* double mutant under free-running conditions. The *bbx18-2 bbx19-3*, together with Col-0 and *bbx19-3* seedlings were entrained under 12-h:12-h LD cycles for 2 weeks and then released to constant light (LL) at 22°C for 5 days. E, F, The daily expression of BBX18 and BBX19 proteins were regulated by the circadian clock, with a peak phase appeared in the morning. The CT phase angles for individual seedlings were plotted against their RAE values to indicate the peak position and the robustness of rhythmicity, respectively (F)

Circadian rhythms were therefore monitored in *BBX* subfamily IV gene mutants. A bioluminescence rhythms assay under free-running conditions (constant light, LL) indicated that only the null mutation of *BBX19* (*bbx19-1*, *bbx19-2*, and *bbx19-3*) significantly shortened the period of self-sustained *CCA1:LUC* expression (23.5 h in *bbx19-3* versus 24.1 h in the wild-type; Figure 1, C and D; Supplemental Figures S1 and S2, and Supplemental Tables S2 and S3). The genome sequence of *BBX19:BBX19* complemented the circadian phenotype of *bbx19* T-DNA insertion mutant lines (Supplemental Figure S2C and Supplemental Table S3). In addition, *bbx19-4*, a CRISPR/Cas9-mediated genome editing mutation line, was created and also showed a shortened circadian period (Supplemental Figure S2, D–F and Supplemental Table S3). BBX19 and BBX18 shared ∼69.4% identity at the amino acid level in evolutionary analyses (Supplemental Figure S3 and Supplemental Data Set S2). Moreover, increased expression of *BBX19* and *BBX18* showed a significant lengthening of the circadian period (24.5 h in *BBX18:BBX18*/Col-0, 24.7 h in *BBX19:BBX19*/Col-0 versus 23.3 h in Col-0), indicating that they function in maintaining the circadian clock (Figure 2, A and B;Supplemental Table S4). In addition, we found that the *bbx18-2 bbx19-3* double mutant had similarly shortened periodicity to that of the *bbx19-3* mutant alone (Figure 2, C and D; Supplemental Table S4). Furthermore, BBX19–GFP and BBX18–GFP fusion proteins were evident in the nucleus (Supplemental Figure S4). The accumulation of BBX19 and BBX18 proteins showed robust oscillations in both light/dark diurnal cycle and LL conditions, in which the BBX19 peak phase occurred around dawn while the peak of BBX18 occurred in the early afternoon (Figure 2, E and F). In summary, BBX19 and BBX18 are involved in adjusting the circadian rhythm.

### BBX19 and BBX18 sequentially interact with PRR9, PRR7, and PRR5 proteins

**Figure 3:**
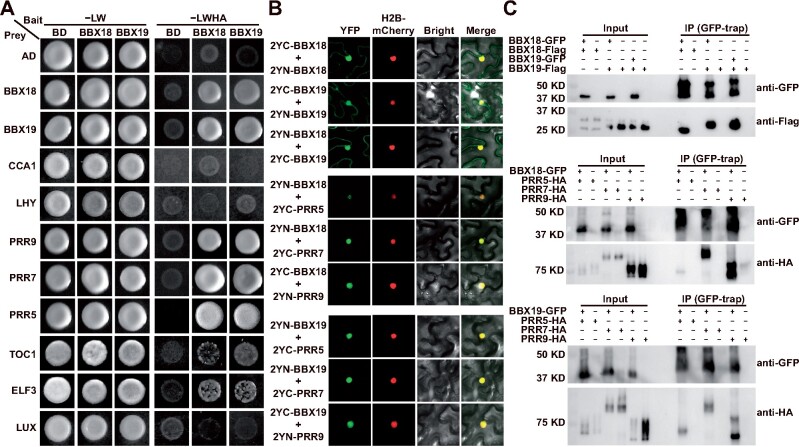
BBX19 and BBX18 physically interact with PRR proteins in vitro and in vivo. A, Yeast two-hybrid system to screen the interacting proteins of BBX18 and BBX19 among the known clock proteins. AD, activating domain; BD, binding domain; -LW, synthetic dropout medium without leucine and tryptophan; -LWHA, selective medium without leucine, tryptophan, histidine, and adenine. B, BiFC assay showing the interaction between BBX18/19 and PRR proteins predominantly occurred in nucleus. Each protein was tagged with either the N- or C-terminal fragment of YFP as indicated. The fluorescent signal in *N. benthamiana* epidermal cells was imaged at 48 h after *A. tumefaciens*-mediated infiltration. C, Co-immunoprecipitation analysis of BBX18, BBX19, and PRRs with transiently expressed proteins in *N. benthamiana*. Anti-GFP antibody was used for performing immunoprecipitation. The proteins were detected with anti-Flag and anti-HA for immunoblotting as indicated

To unravel the underlying mechanism of BBX family genes in circadian regulation, we first used a yeast two-hybrid system to identify whether core oscillators are direct partners of BBX19 and BBX18 (Figure 3A). The results demonstrated that BBX19, BBX18 interacted with PRR9, PRR7, and PRR5, but not CCA1, LHY, and LUX. In addition to complexing with clock proteins, BBX19 and BBX18 proteins also interact with themselves or each other to form homodimers and heterodimers, respectively. BiFC assays were also used to verify that the BBX19, BBX18 dimers, BBX19–BBX18–PRR9, PRR7–PRR5 interactions occur in the nucleus in epidermis cells of the co-infiltrated leaves of *Nicotiana benthamiana* (Figure 3B;Supplemental Figure S5). Moreover, co-immunoprecipitations were further performed with protein extracts from infiltrated *N. benthamiana* leaves using anti-GFP antibody (Figure 3C). Together, BBX19 and BBX18 were characterized as forming protein complexes with PRR proteins.

**Figure 4:**
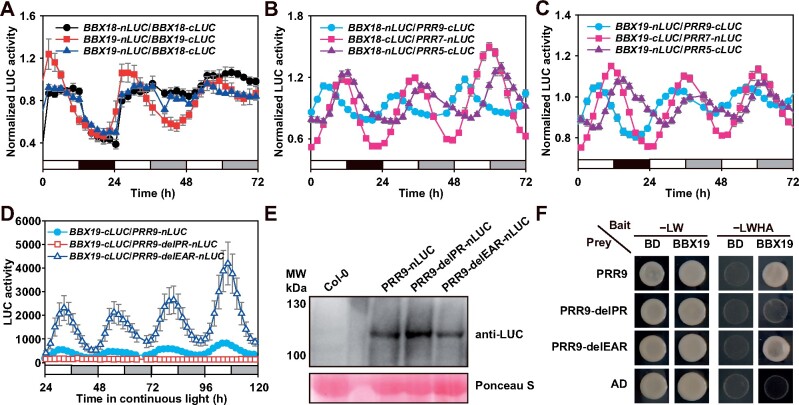
Dynamic protein–protein interactions between BBX19/18 and PRR proteins. A–C, The diurnal and circadian oscillations of the formation of each protein pair. The fusion proteins driven by their own promoters were fused to C-terminal domain of nLUC or cLUC, then the transgenic Arabidopsis plants were generated by genetic cross. The recombined LUC activity in F1 generation was continuously monitored for 72 h with a TopCount luminometer. Data represent mean ± se for three independent experiments. D, Deletion analysis showed that the PR domain of PRR9 is essential for its interaction with BBX19. E, Immunoblot analysis showed the expression of PRR9 in *PRR9-nLUC*, *PRR9-delPR-nLUC*, and *PRR9-delEAR-nLUC* plants. The seedlings were grown under 12-h:12-h LD cycles for 10 days and then sampled at ZT5. Total proteins were separated by 10% sodium dodecyl sulphate–polyacrylamide gel electrophoresis and PRR9 proteins were confirmed by immunoblotting with anti-LUC (AS163691A, from Agrisera). The molecular weight of the PRR9–nLUC fusion protein is expected to be about 99 kDa; PRR9-delPR-nLUC to be about 86 kDa; PRR9-delEAR-nLUC to be about 97 kDa. F, Yeast two-hybrid analysis of BBX19 and PRR9 protein interaction domains

Furthermore, a luciferase complementation analysis was used to check the dynamic interactions of PRRs with BBX19 or BBX18 under both 12-h:12-h light: dark (LD) cycle and LL conditions (Figure 4;Supplemental Figure S6). The expression of the fusion proteins was driven by their own promoters. The formation of BBX19 and BBX18 homodimers and heterodimers all displayed oscillation patterns, and the dynamic interaction of BBX19 homodimer showed good robustness (Figure 4A;Supplemental Figure S6A). In the LD cycle, the BBX19 dimer peaked in the early morning, while the BBX18 dimer lagged slightly. Overall, the dynamic pattern of BBX19–BBX18 interaction is similar to that of the BBX18 homodimer. The protein–protein interactions of BBX19 and BBX18 with PRR9, PRR7, and PRR5 also displayed robust circadian oscillations in LL conditions (Figure 4, B and C; Supplemental Figure S6, B–D), and the interaction peak of each pair occurred at different times of the day, including a BBX–PRR9 peak in the morning, a BBX–PRR7 peak around late afternoon, and a BBX–PRR5 peak in the evening. Also, from the Y2H analysis and recombinant LUC activity, BBX19 showed very weak interactions with TOC1 and ELF3 (Figure 3A;Supplemental Figure S7). Collectively, our findings suggested that BBX19 and BBX18 likely act as partners of sequentially expressed PRR9, PRR7, and PRR5 in the circadian clock.

Ethylene-responsive element binding factor-associated amphiphilic repression (EAR) is a conserved repression motif in plant transcriptional regulators (Kagale and Rozwadowski, 2011), which is necessary for PRR9, PRR7, and PRR5 to interact with TPL family proteins and inhibit *CCA1* and *LHY* expression (Wang et al., 2013). The PR domain is similar to the conserved signal receiver domain of response regulators (Farré and Liu, 2013). To identify which motif mediates protein–protein interactions, we examined the function of N-terminal EAR and PR domains of the PRR9 protein (Figure 4D;Supplemental Figure S6D). The results suggested that deleting EAR caused more robust protein–protein interactions between PRR9 and BBX19. However, the lack of a PR domain resulted in a complete loss of the dynamic protein–protein interactions between PRR9 and BBX19 (Figure 4D). To examine whether deleting the PR or EAR domain affects stability of the PRR9 protein, we analyzed the protein accumulation of PRR9 using immunoblotting (Figure 4E). The levels of PRR9 protein in the wild-type *PRR9-nLUC*, *PRR9-delPR-nLUC*, and *PRR9-delEAR-nLUC* were similar. Also, yeast two-hybrid analysis confirmed that the interaction between BBX19 and PRR9 depends on its PR domain (Figure 4F). In summary, the above results suggested that the PR domain of PRR9 protein is essential for interacting with BBX19 protein, and the EAR domain probably hinders their interaction.

### *PRR* genes are genetically required to regulate *BBX19* in the circadian period

**Figure 5:**
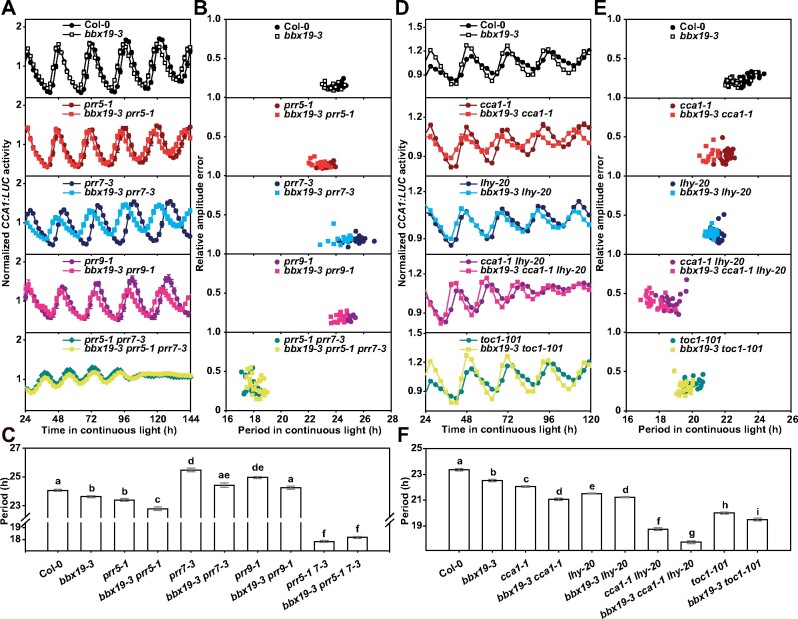
*PRR*s are genetically required for the regulation of *BBX19* on circadian period. A, B, Circadian rhythm of *CCA1:LUC* was measured in the *bbx19-3 prr5-1*, *bbx19-3 prr7-3*, *bbx19-3 prr9-1*, and *bbx19-3 prr5-1 prr7-3* knockout mutants. Arabidopsis seedlings were grown under 12:12 LD cycles, 22°C, for 7 days before transferred to LL for luminescence measurement. The circadian parameters analysis was performed using the fast Fourier transform-nonlinear least squares based on LL24-120 rhythmic traces (A). Period estimation for individual seedlings is plotted against their relative amplitude errors (RAEs value the robustness of rhythmicity) (B). C, Period length estimation of *CCA1:LUC* circadian rhythm (B). Multiple groups were analyzed with one-way ANOVA followed by Tukey’s multiple comparison test, *P* < 0.05. D–F, Circadian rhythm of *CCA1:LUC* was measured in the *bbx19-3 cca1-1*, *bbx19-3 lhy-20*, *bbx19-3 cca1-1 lhy-*20, and *bbx19-3 toc1-101* mutants

To clarify the genetic relationship between *BBX* and *PRR* genes, we generated the *bbx19-3 prr5-1*, *bbx19-3 prr7-3*, *bbx19-3 prr9-1*, and *bbx19-3 prr5-1 prr7-3* mutant lines. Circadian rhythms of *CCA1:LUC* reporter in the mutants were monitored under LL conditions, and variance of circadian period length was compared within groups (Figure 5, A–C; Supplemental Table S5). We found that *bbx19-3 prr7-3* and *bbx19-3 prr9-1* double mutants exhibited a relatively short period, compared with the long period in the *prr7-3* and *prr9-1* single mutants. In addition, both *bbx19-3* and *prr5-1* displayed a shortened period (23.6 h and 23.4 h, respectively), while the *bbx19-3 prr5-1* double mutant had a shorter period length (22.8 h). The consistently shortened phenotype in the *bbx19 prr* double null mutant indicated that *BBX19* potentially imposes a brake on the circadian rhythm. However, the periods in *bbx19-3 prr7-3* and *bbx19-3 prr9-1* were still longer than that in *bbx19-3*. The *bbx19-3 prr5-1 prr7-3* triple mutant (18.2 h) showed a slightly longer period than the *prr5-1 prr7-3* line (17.8 h). In summary, the results suggested an epistatic effect of *prr5*, *prr7*, and *prr9* over *bbx19*.

Furthermore, we found that the period length in the double mutants *bbx19-3 cca1-1* and *bbx19-3 lhy-20* were similar (21.1 h and 21.2 h, respectively), and slightly shorter than single mutants of *cca1-1* and *lhy-20* (Figure 5, D–F; Supplemental Table S6). The period length in the *bbx19-3 cca1-1 lhy-20* triple mutant was about 17.7 h, which is much shorter than *cca1-1 lhy-20* (18.8 h), indicating that *BBX19* acts independently with *CCA1* and *LHY*. In addition, the period of *bbx19-3 toc1-101* was about 20 h, which is slightly shorter than *toc1-101* by about half an hour (Figure 5, D–F; Supplemental Table S6). The data suggested that *bbx19-3* also produces an additive effect to the short period displayed by *prr5-1*, *cca1-1*, *lhy-20*, and *toc1-101*. Given the physical interactions with PRRs and the epistasis of the *prr* null mutant over *bbx19*, our results showed that BBX19 likely regulates the circadian period through the interaction with PRRs.

### Temporal transcriptome analysis of the BBX19-regulated circadian process

**Figure 6:**
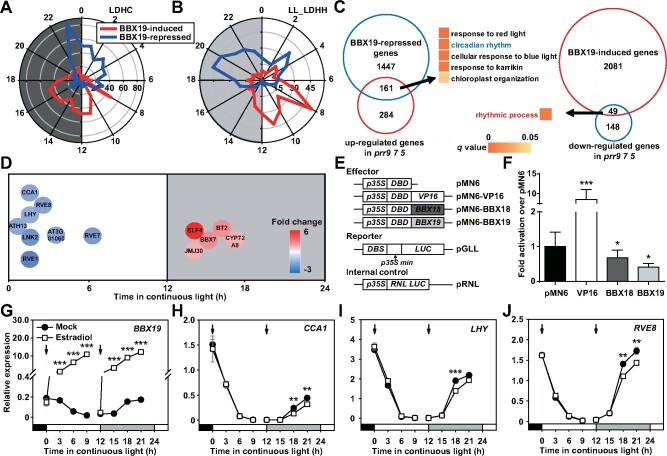
BBX19 inhibits the expression of morning-phased circadian core components. A, B, Radial plots with number of BBX19-controlled genes on the radius and circadian phase (peak phase) on the circumference. For RNA-sequencing, the Arabidopsis seedlings carrying a *pER8-BBX19-YFP-HA* transgene were grown under 12:12 LD cycles for 10 days before *BBX19* were induced with β-estradiol at ZT12. Samples were harvested at ZT2 of the next day for RNA extraction and the subsequent RNA seq experiments. Analysis of DEGs (*P* < 0.05 and fold change >1.5) using the microarray data (http://diurnal.mocklerlab.org/) identified circadian-regulated genes (rhythmic expression under LD and LL conditions). Light and shading represent day and night, respectively. C, GO analysis of the overlapping genes between BBX19-controlled genes and DEGs in the *d975* triple mutant of *PRR9*, *7* and *5* (Nakamichi et al., 2009). D, A plot showing circadian phase of the genes co-regulated by BBX19 and PRR9, PRR7, and PRR5 over the course of a 24-h day. The background color of the letters represents the changes of the genes in the inducible *BBX19* expression lines. E, F, Identifying the transcriptional repressive activity of BBX19 and BBX18 in Arabidopsis protoplasts. Schematic diagrams of the effectors and *LUC* reporter constructs used for transient dual-luciferase transactivation assays in Arabidopsis protoplasts (E). DBD, GAL4 DNA binding domain; DBS, GAL4 DNA binding site; RNL LUC, *Renilla luciferase*. *35S:RLUC*, internal control. BBX19 and BBX18 inhibited the expression of the *LUC* reporter gene (F). The transcriptional activation is indicated by the ratio of LUC/RLUC. Data showing mean ± se for three independent experiments (*P < 0.05; ***P < 0.001 compared to the negative control using Student’s *t* test). G–J, Estradiol-induced *BBX19* expression at subjective night inhibited the transcript accumulation of *CCA1*, *LHY*, and *RVE8* (**P < 0.01; ***P < 0.001; Student’s *t* test). Data show mean ± se of three technical replicates from one of the three independent biological experiments (also shown in Supplemental Figure S8); *IPP2* was used as a normalization control; all experiments yielded congruent results

To further investigate the potential mechanism of *BBX19* in regulating the circadian clock, we used RNA sequencing to profile the circadian transcriptome from *BBX19* inducible overexpression lines (Figure 6;Supplemental Data Set S3). We cloned *BBX19* into the *pER8* vector system to check for inducible expression in transgenic plants (Zuo et al., 2000). Estradiol was applied to *pER8-BBX19* transgenic seedlings at ZT12 to induce excessive accumulation of *BBX19* in the next morning. Analyzing samples taken from ZT2 and oscillated differentially expressed genes (DEGs) using the microarray data (http://diurnal.mocklerlab.org/), we identified several hundred transcripts whose accumulation oscillated with a 24-h period in either LD diurnal cycles or LL conditions (Figure 6, A and B). There were 1,608 genes specifically inhibited by BBX19 (fold change >1.5), 34% of which exhibited diurnal rhythms and 27% of which exhibited circadian rhythms, with peaks appearing around dawn (ZT19-ZT4). Among the genes with increased expression promoted by BBX19, 26% in LD and 20% in LL showed enrichment of rhythmic transcripts, but their peaks appeared from the afternoon to late evening.

Comparing the transcriptome in *BBX19*-inducible overexpression material with the transcriptome in the *prr975* triple mutant (Nakamichi et al., 2009), we found that 36% of the genes up-regulated in *prr975* (161 genes) were inhibited by BBX19 (Figure 6C). Gene ontology enrichment analysis indicated that these 161 genes participated in diverse biological processes including the circadian rhythm and those closely related to the function of the circadian system such as responses to light (Figure 6C, left). Correspondingly, 25% of the genes downregulated in *prr975* (49 genes) were promoted by BBX19, and they were also mainly involved in the circadian processes (Figure 6C, right). We further analyzed the acrophase (peak phase) of genes related to clock regulation and found a few morning-phased genes, including *CCA1*, *LHY*, *RVE8*, and *RVE1*, whose expression was negatively regulated by BBX19; evening-phased genes, including *ELF4* and *JMJ30*, were positively regulated by BBX19 (Figure 6D).

Moreover, the transient gene expression system using Arabidopsis mesophyll protoplasts indicated that BBX19 alone had no transcriptional activation activity, and instead slightly repressed the expression of *LUC* reporter compared to the negative control (Figure 6, E and F). To substantiate the effect of BBX19 on inhibiting gene transcription, we induced the expression of *BBX19* during the day or night, and examined the transcript levels of *CCA1*, *LHY*, and *RVE8* (Figure 6, G–J; Supplemental Figure S8). Estradiol was applied to *pER8-BBX19* materials at ZT0 and ZT12. BBX19 was significantly overexpressed at ZT3 or ZT15 (i.e. 3 h after estradiol treatment), and with the time extension, the transcript levels of *BBX19* were very similar between the two independent treatments (Figure 6G;Supplemental Figure S8A). The overexpression of *BBX19* inhibited the transcript accumulation of *CCA1*, *LHY*, and *RVE8* before dawn, but not during the daytime (Figure 6, H–J; Supplemental Figure S8, B–D).

**Figure 7:**
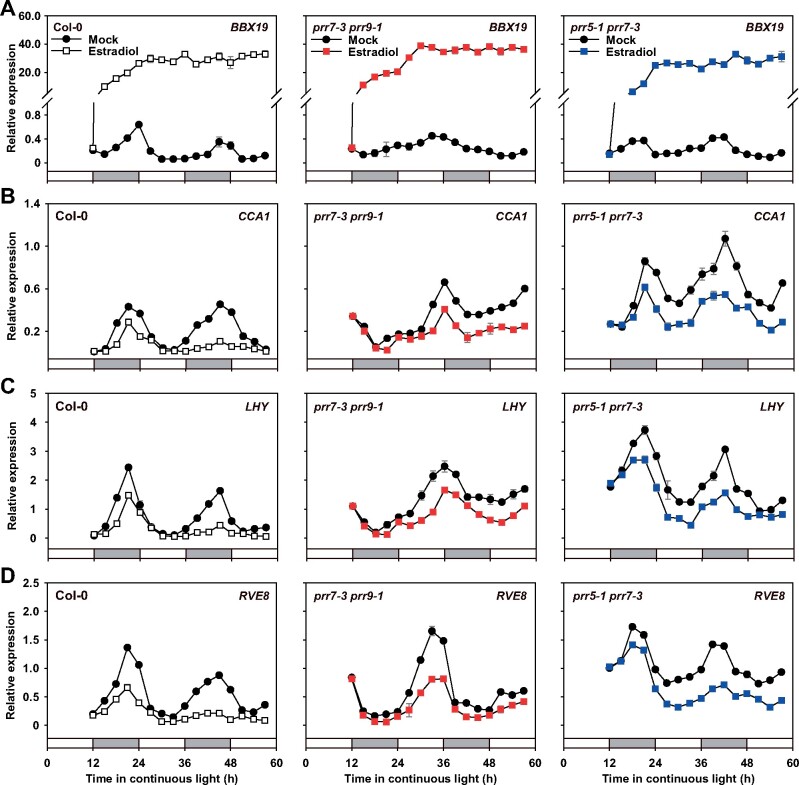
BBX19 inhibits the accumulation of *CCA1*, *LHY*, and *RVE8* transcripts. The wild-type (Col-0), *prr7-3 prr9-1*, and *prr5-1 prr7-3* mutants containing *pER8-BBX19* were grown under 12:12 LD cycles for 10 days before *BBX19* were induced at ZT12 with β-estradiol (A). quantitative reverse transcription polymerase chain reaction analysis of the transcript accumulation of *CCA1* (B), *LHY* (C), and *RVE8* (D) in the Col-0, *prr7-3 prr9-1*, and *prr5-1 prr7-3* mutants. Data show mean ± se of three technical replicates from one of three independent biological experiments (also shown in Supplemental Figure S9); *IPP2* was used as a normalization control; all experiments yielded congruent results. White or gray bars represent subjective day or subjective night, respectively

After BBX19 overexpression, transcript accumulation was monitored under LL conditions for 48 h (Figure 7;Supplemental Figure S9). The results showed that accumulation of *CCA1*, *LHY*, and *RVE8* transcripts began to decline in Col-0 within 12 h after treatment with estradiol. After that, the level of transcripts for each gene was extremely low. Hence, we proposed that, after dawn, the transcription of *CCA1*, *LHY*, and *RVE8* is already declining or at a trough, and the effect of overexpressing *BBX19* is not significant during the day. However, from evening to dawn, when the transcripts of *CCA1*, *LHY*, and *RVE8* would be rising, overexpressing *BBX19* will significantly inhibit those target genes. In addition, we further analyzed the function of BBX19 overexpression on morning-phased genes in the *prr7-3 prr9-1* and *prr5-1 7-3* mutants. The results showed that the inhibitory effect of *BBX19* on *CCA1*, *LHY*, or *RVE8* expression in the mutants was significantly weakened compared to the wild-type (Col-0), especially on the second day after inducing *BBX19*, when the transcription peaks of *CCA1*, *LHY*, and *RVE8* in the wild-type were strongly suppressed (Figure 7;Supplemental Figure S9). The data predicted that *PRR9*, *PRR7*, and *PRR5* are required for *BBX19* to negatively regulate the expression of *CCA1*, *LHY*, or *RVE8*. Therefore, combined with transcriptome analysis, we proposed that *BBX19* maintains the endogenous circadian rhythm by modulating the expression of morning-phased clock components such as *CCA1*, *LHY*, and *RVE8*.

### PRR9, PRR7, and PRR5 are involved in the binding of BBX19 to the *CCA1* promoter and inhibit its transcription

**Figure 8:**
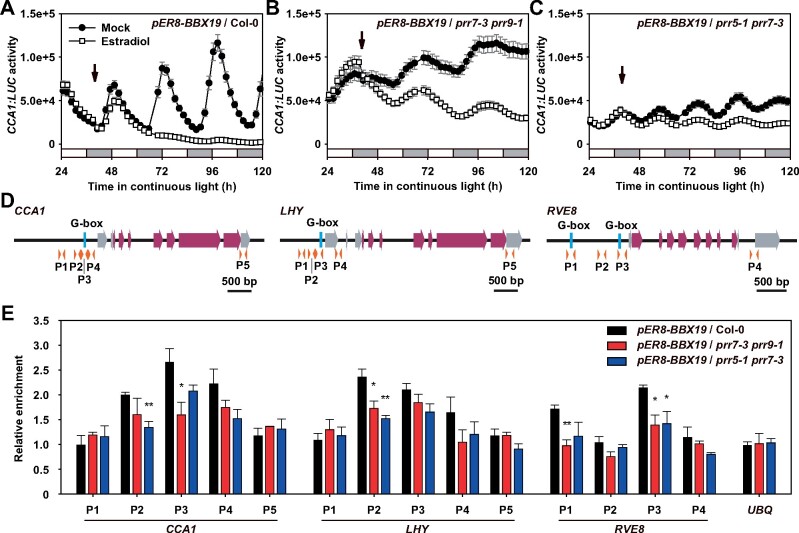
PRR9, PRR7, and PRR5 are required for the association of BBX19 with *CCA1* promoter and inhibit its transcription. A–C, Measurement of *CCA1:LUC* activity in the *prr7-3 prr9-1* and *prr5-1 prr7-3* mutant with or without the induced expression of *BBX19*. Arabidopsis seedlings carrying *pER8-BBX19* were grown under 12:12 LD cycles for 7 days before transferred into LL and treated with β-estradiol at CT39. *LUC* activity was measured in LL using a TopCount luminometer. D, Schematic diagram of *CCA1*, *LHY*, and *RVE8* gene structure including the upstream region. G-box elements in the promoter region (blue vertical bar), exon (purple box with arrow), 5′ and 3′ untranslated region (gray box with arrow), and orange arrow heads below represent the location of primers used in chromatin immunoprecipitation quantitative real-time polymerase chain reaction (ChIP-qPCR) assay. E, ChIP-qPCR assay of BBX19-YFP-HA protein in Col-0, *prr7-3 prr9-1*, and *prr5-1 prr7-3* mutants with promoters of *CCA1*, *LHY*, and *RVE8*. Seedlings were grown under 12:12 LD cycles for 14 days before BBX19 were induced at ZT12 with β-estradiol. Sampling was performed at ZT3 when BBX19 expression reached a significant peak. Anti-HA antibody was used for precipitating of BBX19 protein, followed by qPCR detection. For relative enrichment of DNA fragments, the ratios between the levels of immuno-precipitated DNA in signal samples (using anti-HA antibody) and in reference samples (no antibody) were calculated. Data represent mean ± se of three biological replicates (**P < 0.01; *P < 0.05; Student’s *t* test)

To investigate the effect of BBX19 in clock gene expression in real time, we examined the promoter activity of *CCA1* in *pER8-BBX19* materials. We found that estradiol treatment did not affect *CCA1:LUC* activity in the wild-type (Supplemental Figure S10A), but overexpressing *BBX19* significantly inhibited *CCA1:LUC* activity (Figure 8A), indicating its inhibitory function on transcription of *CCA1*. The inhibitory effect of *BBX19* overexpression on *CCA1:LUC* activity was markedly blocked in the *prr7-3 prr9-1* and *prr5-1 prr7-3* mutants (Figure 8, B and C). In addition, overexpressing *BBX19* caused a lengthened period and slightly reduced the circadian amplitude of *TOC1:LUC* in free-running conditions (Supplemental Figure S10B), consistent with the circadian phenotype of *CCA1:LUC* in *BBX19:BBX19*/Col-0 plants (Figure 2, A and B). Collectively, these results indicated that BBX19 and its interacting proteins, PRR9, PRR7, and PRR5, jointly modulated morning clock gene expression.

Given the physical interactions between BBX19 and PRR9, PRR7, and PRR5, together with the genetic requirement for PRR9, PRR7, and PRR5 in regulating the circadian period, chromatin immunoprecipitation was used to compare the relative abundance of BBX19 protein within the promoter regions of its putative target genes, *CCA1*, *LHY*, and *RVE8* (Figure 8, D and E). Chromatin was isolated from *BBX19-YFP-HA*/Col-0, *BBX19-YFP-HA*/*prr7-3 prr9-1*, and *BBX19-YFP-HA*/*prr5-1 7-3* seedlings, which were harvested at ZT3 to match the peak expression of *BBX19* in a 24-h day. The results showed a significant association of BBX19 in Col-0 plants with the *CCA1* promoter, and the regions around the G-box were necessary to mediate the transcriptional regulation (Figure 8E). In the *prr7-3 prr9-1* or *prr5-1 7-3* mutants, the associations of BBX19 with *CCA1*, *LHY*, and *RVE8* promoter regions were weakened (Figure 8E), and the *prr7-3 prr9-1* and *prr5-1 7-3* mutations did not affect the accumulation of BBX19 protein (Supplemental Figure S11). Thus, the data suggested that protein complexes formed by BBX19 and PRR9, PRR7, and PRR5 might facilitate their binding to common target genes. Together, our data demonstrated that BBX19 negatively regulates morning-phased clock gene expression by forming protein complexes with PRRs.

## Discussion

The transcript and protein accumulation of CCA1 exhibited a robust 24-h rhythm, reaching a peak immediately after dawn, and then its expression was continuously suppressed until the night, when the *CCA1* transcript level reached a trough and then began to be enriched again (Yakir et al., 2009); the mechanism of this is unclear. PRR9, PRR7, PRR5, and TOC1 are expressed sequentially throughout the day, and act as inhibitors to regulate the expression of *CCA1* and *LHY* (Nakamichi et al., 2010; Gendron et al., 2012; Huang et al., 2012). Previous results of Chromatin immunoprecipitation (ChIP)-sequencing show that PRR proteins, including PRR9, PRR7, and PRR5 associate to chromatin regions rich in G-box-like motifs, and distinct PRR-targeted genes include the morning-phased clock genes, *CCA1*, *LHY*, *RVE1*, *RVE2*, *RVE7*, *RVE8*, and the transcriptional cofactor genes *LNK1*, *LNK2*, *LNK3*, and *LNK4* (Liu et al., 2016). Here, we identified a member of BBX subfamily IV with DNA binding activity, BBX19, which acted on the self-sustained circadian rhythm (Figures 1 and 2). Chromatin immunoprecipitation analysis showed that BBX19 preferentially associated to the chromatin region containing a G-box element (Figure 8) and negatively regulated the expression of morning-phased core clock genes, including *CCA1*, *LHY*, and *RVE8*. In the *prr9-1 prr7-3* and *prr5-1 7-3* mutants, the binding ability of BBX19 with *CCA1*, *LHY*, and *RVE8* promoters was weakened, together with the physical interaction between BBX19 and PRR proteins, indicating that BBX19 regulates the transcription process by interacting with PRR proteins.

**Figure 9:**
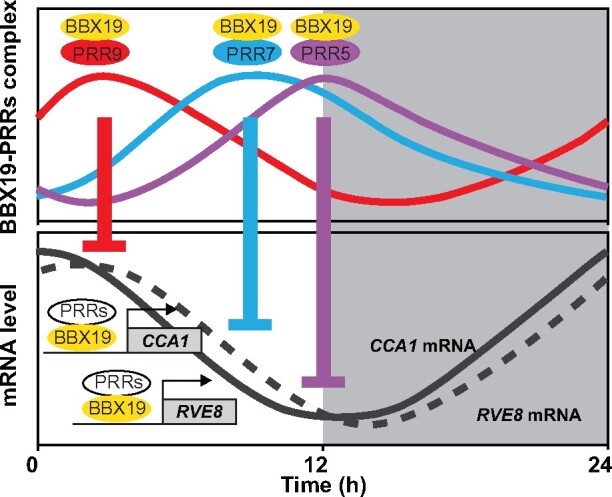
A proposed working model for the dynamic formation of BBX19–PRRs complex over a 24 h in regulating the *CCA1* and *RVE8* expression. Zinc finger transcription factor, BBX19 protein, is expressed during the daytime. Sequentially expressed PRR9, PRR7, and PRR5 interact with BBX19 in precise temporal ordering from dawn to dusk. PRR proteins affect BBX19 recruitment to the *CCA1* and *RVE8* promoters. BBX19–PRRs complexes function directly in transcriptional regulation of the circadian clock to orchestrate circadian rhythms

In this study, it was noteworthy that the protein–protein interactions between PRR9, PRR7, PRR5 and BBX19 displayed robust circadian oscillations over a 24-h day, with the BBX19-PRR9 protein pair peak appearing at noon, BBX19-PRR7 peaking in late afternoon, and BBX19-PRR5 peaking in the evening (Figures 3 and 4). Our results hence revealed a dynamic molecular mechanism in which BBX19, a zinc-finger transcription factor, interacts with PRR9, PRR7, and PRR5 sequentially from early morning to evening, to directly inhibit *CCA1*, *LHY*, and *RVE8* expression (Figure 9). Previously, BBX19 was also reported to interact with ELF3 and then be degraded by COP1 to participate in the formation of clock ELF3–ELF4–LUX evening complex (Wang et al., 2015). Regarding how PRRs regulate the transcription of target genes, there are two possible mechanisms based on previous studies. Early studies shown that TOC1 and PRR5 can directly bind to the promoter through the CCT domain, and the latest studies have shown that PRRs can also be recruited by PIFs and indirectly bind to G-box cis-elements on the promoters of target genes (Gendron et al., 2012; Nakamichi et al., 2012; Zhu et al., 2016; Zhang et al., 2020). Studies have also shown that TPL can interact with the EAR motif of PRR and contribute to the inhibitory effect of PRRs (Wang et al., 2013).

The plant Groucho/TUP1 family component has been identified as transcriptional corepressor of the circadian clock (Wang et al., 2013). TPL physically interacts with PRR9, PRR7, and PRR5 separately, and jointly bound to the promoters of *CCA1* and *LHY* in the ChIP assay. Dysfunction of *TPL* causes increased levels of *CCA1* and *LHY* transcripts, as well as a lengthened circadian period. As the common interacting protein of TPL and BBX19, the working model for PRR9, PRR7, and PRR5 sequential expression on *CCA1* transcriptional regulation has become more complicated. Notably, the peak of *TPL* transcript and protein enrichment occurs around dawn of a 24-h day (Wang et al., 2013), which is quite different from the peak expression of BBX19 in the morning (Figure 2, E and F). TPL interacts with the EAR motif of PRRs. However, we found that BBX19 interacted with the PR domain, but the interaction between BBX19 and PRR9 was even augmented when EAR is missing (Figure 4D), implying that the regulatory mechanism for PRRs, BBX19, and TPL needs to be further investigated. In addition, BBX19 was previously reported to have particularly high expression in the vasculature (Wang et al., 2014). Therefore, it would be helpful to analyze the genetic relationship between *TPL* and *BBX19* in the circadian system, and to examine the spatial and temporal organization of TPL and BBX19 in the circadian clock and clock outputs. Nonetheless, our findings provided new insights into how the circadian clock finely regulates growth and development.

Previously, BBX19 was shown to act similarly to BBX21 of the BBX IV family in mediating photomorphogenesis: BBX21 specifically binds to the T/G-box (CACGTT) element in the *HY5* promoter but activates its expression (Xu et al., 2016), while PRRs have inhibitory roles in the transcriptional regulation of circadian oscillators. Here, we found that BBX19 significantly inhibited *CCA1* promoter activity through interacting with PRR proteins (Figure 8, A and C). In the *prr7-3 prr9-1* or *prr5-1 prr7-3* mutants, the amplitude of *CCA1:LUC* rhythmic expression was significantly rescued compared to the wild-type material, indicating that PRRs are necessary for the inhibitory effect of BBX19 on *CCA1* expression. The expression pattern of *BBX19* is very similar to that of *CCA1*. The transcription and translation of both start around midnight and peak in the morning. Based on our results, the inhibitory effect of BBX19 on the accumulation of *CCA1*, *LHY*, and *RVE8* is likely to start at midnight. This implied that BBX19–PRRs worked as a transcriptional repressor complex involved in regulating transcription initiation of morning-phased circadian oscillators.

In view of this, the other BBX IV transcription factors may form a transcription repressive complex with certain components in a similar way as BBX19–PRRs and function directly in the temporal and spatial expression of their target genes. The overexpression of *COL1* and *COL2*, which belongs to the BBX subfamily, leads to a short-period phenotype (Ledger et al., 2001). PRRs interact with the CO protein of the BBX family to stabilize CO, thereby regulating photoperiod-dependent flowering. Also, the results of chromatin immunoprecipitation quantitative real-time polymerase chain reaction indicate that CO in the *prr975 toc1* quadrant cannot bind to the FT promoter region (Hayama et al., 2017). In addition to BBX19, there are a few members from different BBX subfamilies that participate in circadian clock-related transcriptional regulation, and there may be synergy or antagonism among them.

Circadian core components—such as CCA1, PRR7, and ELF3—regulate multiple physiological outputs, such as hypocotyl elongation, in response to photoperiodic zeitgebers (Harmer, 2009; Lu et al., 2012; Martin et al., 2018; Zheng et al., 2018). We further investigated whether BBX19 also responds to light. Although the lack of *BBX19* altered circadian periodicity (Figure 1), we found that the trend of the phase response curve to light pulses and the fluence-rate response curve were consistent with that of the wild-type, indicating that the responsiveness of the circadian clock in the *bbx19-3* to external light signals was not affected (Supplemental Figure S12). We speculate that there may be zeitgebers other than light that reset the circadian clock via BBX19. Our results provide a molecular mechanism enhancing in-depth understanding of the fine regulation mechanism of PRRs, which may help elucidate how the circadian clock regulates growth and development in the future.

## Materials and methods

### Plant materials and growth conditions

The following T-DNA insertion lines were obtained from the Arabidopsis Biological Resource Center (ABRC, Ohio State University): *bbx18-2* (SALK_061956), *bbx19-1* (SALK_088902), *bbx19-2* (SALK_087493), *bbx19-3* (SALK_032997), *bbx20-1* (CS878932), *bbx21-2* (SALK_105390), *bbx22-1* (SALK_105367), *bbx23-1* (SALK_053389), *bbx24-1* (SALK_067473), and *bbx25-3* (CS2103310). The *cca1-1 lhy-20* double mutant, in which *cca1-1* is in a Ws background (Green and Tobin, 1999), was created by backcrossing six times with *lhy-20* in a Col-0 background (Michael et al., 2003). *toc1-101* was a gift from Peter Quail (Kikis et al., 2005). Arabidopsis seeds were sterilized in 20% bleach before being placed on 1/2 Murashige and Skoog (MS) medium (M524, PhytoTechnology Laboratories) plus 2% sucrose, and then stratified for 3 days at 4°C in the dark. Plants were grown under a 12:12 LD cycle (white light, 70 μmol m^−2^ s^−1^) at 22°C in a growth chamber (Percival CU-36L5).

### Constructs

For the split luciferase complementation assays, constructs were produced following the method described previously (Li et al., 2020). Full-length *BBX18*, *BBX19*, *PRR9*, *PRR7*, and *PRR5* genomic DNAs were amplified from Col-0 genomic DNA with primer pairs BBX18-F/BBX18-R and BBX19-F/BBX19-R (Supplemental Table S7), then PCR products were cloned into the pENTR 1A vector. Two *Sfi*I sites were inserted just before the stop codons of *BBX18*, *BBX19*, *PRR9*, *PRR7*, and *PRR5* through PCR amplification with primer pairs *BBX18*-SfiI-F/*BBX18*-SfiI-R and *BBX19*-SfiI-F/*BBX19*-SfiI-R (Supplemental Table S7). PCR products of either *LUC*, *nLUC* or *cLUC* with *Sfi*I sites at both ends were amplified and then cloned to create in-frame translational fusions. The donor vectors with *BBX18*, *BBX19*, *PRR9*, *PRR7*, and *PRR5* were finally recombined into binary vectors. Constructs consisting of *PRR9* lacking the PR domain (*PRR9-delPR*, 118 amino acids, positions 38–156) or EAR domain (*PRR9-delEAR*, 20 amino acids, positions 250–269) were fused to the N-terminal domain of *LUC* (*nLUC*) before being transformed into *BBX19-cLUC*/Col-0 plants. To generate an estradiol-inducible pER8 expression vector, the *BBX19* CDS sequences were amplified by PCR before they were inserted into pENTR/SD/D-TOPO (Invitrogen), and were then recombined by LR reaction Gateway technology into destination vector pER8-GW (Papdi et al., 2008).

The *bbx19-4* Cas9-free mutant was generated using a CRISPR/Cas9 approach according to the previously published paper (Gao et al., 2016). The target sequence was cloned into the U6-gRNA unit, then the U6-gRNA unit was assembled into the *pHDE-35SCas9-mCherry* vector though the *PmeI* site. The *bbx19-4* CRISPR/Cas9 constructs were transformed into Arabidopsis using the floral dip method. T1 plants were screened on MS medium with hygromycin, genomic DNA samples extracted from T1 plants were used as templates for PCR, and *bbx19-4*-F(PCR) and *bbx19-4*-R(PCR) primers were used to amplify the fragment containing the target site for Sanger sequencing. Cas9-free T2 seeds were separated by a fluorescence microscope according to the mCherry signals and the Cas9-free T2 plants were sequenced to obtain homozygous genome-editing plants. All primer sequences are listed in Supplemental Table S7.

### Circadian rhythm measurement

The luciferase reporter gene fusion *CCA1:LUC* was introduced into the wild-type and *bbx18-bbx2*5 mutant lines. Transgenic seedlings were entrained under 12:12 LD cycles for 7 days before they were grown in constant light (LL) at 22°C for 5 days. Circadian rhythms of *LUC* activity were captured using a back-illuminated CCD sensor from e2v (CCD47-40) and normalized to the mean value over the time series. Fast Fourier transform-nonlinear least squares analysis of circadian parameters were conducted on a data window of ZT24-120. The bioluminescence activity of *BBX18:BBX18-LUC* and *BBX19:BBX19-LUC* fusion proteins were measured on a Packard TopCount^TM^ luminometer and used as a read-out of the state of *BBX18* and *BBX19* under LD (ZT0-48) and LL (ZT48-120) conditions.

### Temporal transcriptome (RNA-seq) analysis

Seedlings of *pER8:BBX19-YFP-HA*/Col-0 were grown under 12:12 LD cycles at 22°C for 10 days, and then were treated by 30-μM β-estradiol or mock at ZT12. The materials were collected at ZT2 of the next morning and were immediately frozen in liquid nitrogen. RNA-seq libraries were prepared using the Illumina Directional mRNA-Seq Library Preparation Kit and sequenced on an Illumina HiSeq 2000, resulting in single-end 50-bp reads in each sample. RNA sequencing produced an average of 10.9 million reads for the mock sample and an average of 11.2 million reads for the estradiol sample. Sequence reads were aligned to the TAIR10 genome and analyzed using CLC Genomics Workbench 11 software (Qiagen). The ratio of reads mapped to the reference genome in the two groups was 99.59% and 98.86%, respectively. DEGs between the estradiol- and mock-treated *pER8-BBX19-YFP-HA*/Col-0 transgenic plants were identified by a significance analysis when the change was more than 1.5-fold with *P* < 0.05. The diurnal rhythm and circadian rhythm of DEGs were identified using microarray data (http://diurnal.mocklerlab.org/). Gene ontology (GO) term enrichment analysis for the DEGs was performed using PANTHER (http://www.pantherdb.org;Mi et al., 2013).

### Co-immunoprecipitation assays

To generate *pCsVMV:PRRs-HA-1300* constructs, full-length *PRR9*, *PRR7*, and *PRR5* coding sequence were amplified and inserted into the vector of *pCsVMV:HA-1300*. Fragments containing the ORFs of *BBX18* and *BBX19* were separately inserted into *pCsVMV:GFP-1300* and *2 × 35S:FLAG-1307* vectors. All primer sequences are listed in Supplemental Table S7. The combinations of *Agrobacterium* carrying the indicated vectors were co-infiltrated into the leaves of 5-week-old *N. benthamiana*, and the samples were collected after 3 days of infiltration. Protein extraction and immunoprecipitation assays were performed following a method described previously (Wang et al., 2013) using GFP-Trap (GTMA-20, ChromoTek) magnetic beads. The incubation was about 1 h at 4°C followed by washing four times with protein extraction buffer using a magnetic stand. For immunoblot detection, GFP antibody (Cat#ab6556, Abcam), HA antibody (Cat#11867423001, Roche), and FLAG antibody (Cat#M20008M, Abmart) were used to detect the tagged proteins.

### ChIP assays

ChIP assays were performed following a previously described method (Saleh et al., 2008). Seedlings of *pER8-BBX19-YFP-HA*/Col-0, *pER8-BBX19-YFP-HA*/*prr7-3 prr9-1*, and *pER8-BBX19-YFP-HA*/*prr5-1 prr7-3* were grown under 12:12 LD cycles at 22°C for 2 weeks, and then treated with 30-μM β-estradiol at ZT12. The materials were harvested and cross-linked with 1% formaldehyde at ZT3 of the next morning. Protein G-Agarose beads (Roche, Cat. # 11243233001) and an anti-HA antibody (Sigma-Aldrich, Cat. #H3663) were used for ChIP analysis. Primers amplifying a fragment in *UBQ* were used for the negative control. All primer sequences are listed in Supplemental Table S7.

### Phylogenetic analysis

For the phylogenetic tree, sequence information on different plants was retrieved via a BLASTP search of Phytozome 12 (https://phytozome.jgi.doe.gov/pz/portal.html). Sequence alignments and evolutionary analyses were performed with the software MEGA 7 (Kumar et al., 2016). Multiple sequence alignments were performed using ClustalW and phylogenic trees were generated using the neighbor-joining method (Saitou and Nei, 1987). Statistical support of the nodes was calculated with the bootstrap method with 1,000 replicates (Felsenstein, 1985).

### Accession numbers

Sequence data for the genes described in this article can be found in the GenBank/EMBL databases under the following accession numbers: *BBX18* (AT2G21320), *BBX19* (AT4G38960), *BBX20* (AT4G39070), *BBX21* (AT1G75540), *BBX22* (AT1G78600), *BBX23* (AT4G10240), *BBX24* (AT1G06040), *BBX25* (AT2G31380), *CCA1* (AT2G46830), *LHY* (AT1G01060), *RVE8* (AT3G09600), *TOC1* (AT5G61380), *PRR5* (AT5G24470), *PRR7* (AT5G02810), *PRR9* (AT2G46790), *ELF3* (AT2G25930), *LUX* (AT3G46640), *IPP2* (AT3G02780), and *UBQ* (AT4G05320).

## Supplemental data

The following materials are available in the online version of this article.

**Supplemental Figure S1.** Circadian rhythms of *CCA1:LUC* in *BBX* subfamily IV gene mutation lines under free-running conditions.

**Supplemental Figure S2.** Circadian rhythms in the *BBX19* mutation and complementation lines.

**Supplemental Figure S3.** Phylogenetic assessment of AtBBX18 and AtBBX19 orthologs in land plants.

**Supplemental Figure S4.** Subcellular localization of BBX18 and BBX19.

**Supplemental Figure S5.** Negative controls for BiFC assays.

**Supplemental Figure S6.** LUC bioluminescence analysis showed dynamic protein–protein interactions between BBX19/18 and PRR proteins.

**Supplemental Figure S7.** Dynamic protein–protein interactions between BBX19 and TOC1, ELF3 proteins.

**Supplemental Figure S8.** Estradiol-induced *BBX19* expression at subjective night inhibited the transcript accumulation of *CCA1*, *LHY*, and *RVE8*.

**Supplemental Figure S9.** BBX19 inhibits the accumulation of *CCA1*, *LHY*, and *RVE8* transcripts.

**Supplemental Figure S10.** *BBX19* overexpression leads to the reduced amplitude and lengthened period of *TOC1:LUC*.

**Supplemental Figure S11.** Inducible expression of BBX19 protein in the *BBX19-YFP-HA* transgenic lines.

**Supplemental Figure S12.** Characteristics of circadian rhythms in response to environmental light cues.

**Supplemental Table S1.** Five genes of BBX subfamily IV, co-expressed with *CCA1* and *LHY* in multiple microarray- and RNAseq-based coexpression data sets in ATTED-II (http://atted.jp), were highly ranked in the co-expression list.

**Supplemental Table S2.** Period length of *CCA1:LUC* circadian rhythms shown in Figure 1, C and D.

**Supplemental Table S3.** Period length of circadian rhythms shown in Figure S2.

**Supplemental Table S4.** Period length of *CCA1:LUC* circadian rhythms shown in Figure 2, A–D.

**Supplemental Table S5.** Period length of *CCA1:LUC* circadian rhythms shown in Figure 5, A–C.

**Supplemental Table S6.** Period length of *CCA1:LUC* circadian rhythms shown in Figure 5, D–F.

**Supplemental Table S7.** Oligonucleotides (shown 5′ to 3′) used in this study.

**Supplemental Data Set S1.** Text file of the alignment used for the phylogenetic analysis shown in Figure 1B.

**Supplemental Data Set S2.** Text file of the alignment used for the phylogenetic analysis shown in Supplemental Figure S3.

**Supplemental Data Set S3.** RNA sequencing of the circadian transcriptome from *BBX19* inducible overexpression lines shown in Figure 6, A–D.

## Supplementary Material

koab133_Supplementary_Data

## Notes

These authors contributed equally to this work (L.Y., Y.Y.).

X.X., Q.X., and L.W. conceived the project and wrote the article. L.Y. and Y.Y. analyzed circadian rhythm with *BBX*s gene mutants. L.Y. constructed *pER8-BBX19* expression vector, generated genetic materials, performed temporal transcriptome, RT-PCR, dynamic protein–protein interactions, and ChIP analysis. Y.Y. performed confocal imaging, completed Y2H, BiFC, and Co-IP analysis and related constructs. M.L. performed the WB assay, Y.S. performed RT-PCR assay, H.L. completed partial Y2H construct. Q.X. and Q.W. completed the constructs of PRRs for LCA analysis. X.X., Q.X., L.Y., Y.Y. and L.W. analyzed the data.

The authors responsible for distribution of materials integral to the findings presented in this article in accordance with the policy described in the Instructions for Authors (https://academic.oup.com/plcell) are: Xiaodong Xu (xiaodong.xu@henu.edu.cn), Lei Wang (wanglei@ibcas.ac.cn), and Qiguang Xie (qiguang.xie@henu.edu.cn).
