# Supplementary material for: BBX19 fine-tunes the circadian rhythm by interacting with PSEUDO-RESPONSE REGULATOR proteins to facilitate their repressive effect on morning-phased clock genes
Source: Plant Cell. 2021 May 14;33(8):2602–17. doi: 10.1093/plcell/koab133 (PMC8408442; doi:10.1093/plcell/koab133)
Supplement: koab133_Supplementary_Data [file koab133_supplementary_data.zip › tpc.00221.2021-s02.pdf]

>BBX18
MRILCDACESAAAIVFCAADEAALCCSCDEKVHKCNKLASRHLRVGLADP-------
SNAPSCDICENAPAFFYCEIDGSSLCLQCDMVVHVG--GKRTHRRFLLLRQRIEFPGDKPNHADQLG-
LRCQKASSGRGQE---------------SNGNGDHDHNMIDLNSNPQRVHEPGS------------
HNQEEGIDVNNANNHEHE---

>BBX19
MRILCDACENAAAIIFCAADEAALCRPCDEKVHMCNKLASRHVRVGLAEP-------
SNAPCCDICENAPAFFYCEIDGSSLCLQCDMVVHVG--
GKRTHGRFLLLRQRIEFPGDKPKENNTRDNLQNQRVSTNGNGE---------------ANG--
KIDDEMIDLNANPQRVHEPSS------------NNN--GIDVNNENNHEPAGLVP---------------
---VGPFKRESEK--

>BBX20
MKIWCAVCDKEEASVFCCADEAALCNGCDRHVHFANKLAGKHLRFSLTSP-----
TFKDAPLCDICGERRALLFCQEDRAILCRECDIPIHQANEHTKKHNRFLLTGVKISASPSAYPRASNSNS
AAAFGRAKTRPKSVSSEVPSSASNEVFTSSSSTTTSNCYYGIEENYHHVSDSGSGSGCTGSISEYLMETL
PGWRVEDLLEHPSCVSY--------------------EDNIITNNNNNSES--------------------------
------YRVYDGSSQF---------------------HHQG-----FWDHKPFS--------------------
>BBX21
MKIRCDVCDKEEASVFCTADEASLCGGCDHQVHHANKLASKHLRFSLLYPS---
SSNTSSPLCDICQDKKALLFCQQDRAILCKDCDSSIHAANEHTKKHDRFLLTGVKLSATSSVYKPTSKSS
SSSSSNQDFSVPGSSISNPPP--LKKPLSAPPQSNKIQPFSKINGGDASVNQWGS----
TSTISEYLMDTLPGWHVEDFLDS-
SLPTYGFSKSGDDDGVLPYMEPEDDNNTKRNNNNNNNNNNNNTVSLPSKNLGIWVPQIPQTLPSSY
PNQYFSQDNNIQFGMYNKETSPEVVSFAPIQNMKQQGQNNKRWYDDGGFTVPQITPPPLSSNKKF
RSFW
>BBX22
MKIQCNVCEAAEATVLCCADEAALCWACDEKIHAANKLAGKHQRVPLS------
ASASSIPKCDICQEASGFFFCLQDRALLCRKCDVAIHTVNPHVSAHQRFLLTGIKVGLESIDTGPSTKSS
PTNDDKTMETKPFVQSIPEPQKMAFDHHHHQQQQEQQEGVIPGTKVNDQTSTKLP-----
LVSSGSTTGSIPQWQIEEIFGLTDFDQSYEYMENNGS-------
SKTDVLKMKLLDSACLGKKLEKADTSRRGDSDSSSMMRSAEEDGEDNNNCLGGETSWAVPQIQSPP
TASGLNWPKHFHHHSVFVPDITSSTPYTGSSPNQRVGKRRRRF----
>BBX23
MKIQCEVCEKAEAEVLCCSDEAVLCKPCDIKVHEANKLFQRHHRVALQKDAASATTASGAPLCDICQ
ERKGYFFCLEDRAMLCNDCDEAIHTCN----SHQRFLLSGVQV-----------------SD----------
QSLTE----------------------------NSECST----------SFSSET-----YQIQSKVSLN-------
--SQYSS------EETEAGN---
SGEIVHKNPSVILSP--
>BBX24
MKIQCDVCEKAPATVICCADEAALCPQCDIEIHAANKLASKHQRLHLNSLS------
TKFPRCDICQEKAAFIFCVEDRALLCRDCDESIHVANSRSANHQRFLATGIKVALTSTICSKEIEKNQPEP
SNNQQKANQIPAKSTSQ--------QQQQPSSATPLPWAVDDFFHFSDIES------TDK-

KGQLDLGAGELDWFSDMGFFGDQI--
--------NDKALPAAEVPELSVSHLGHVHSY-KPMKSNVSHKKPRFETRYDDDDEEHFIVPDLG----
----
>BBX25
MKIQCDVCEKAPATLICCADEAALCAKCDVEVHAANKLASKHQRLFLDSLS------
TKFPPCDICLEKAAFIFCVEDRALLCRDCDEATHAPNTRSANHQRFLATGIRVALSSTSCNQEVEKNHF
DPSN-QQSLSKPPT----------------QQPAAPSPL-WATDEFFSYSDLDC------
SNKEKEQLDLG--ELDWLAEMGLFGDQP--
------------DQEALPVAEVPELSFSHLAHAHSYNRPMKSNVPNKKQRLEYRYDDE-
EEHFLVPDLG--------------

(BBX18:0.04570944,BBX19:0.06655786,((BBX20:0.17445276,BBX21:0.20912963)0.9980:0.13472892,((BBX22:0.19087294,BBX23:0.25984875)0.9100:0.08097744,(BBX24:0.07721644,BBX25:0.11757189)1.0000:0.19891049)0.6370:0.03556241)1.0000:0.47396745);
