# Supplementary material for: BBX19 fine-tunes the circadian rhythm by interacting with PSEUDO-RESPONSE REGULATOR proteins to facilitate their repressive effect on morning-phased clock genes
Source: Plant Cell. 2021 May 14;33(8):2602–17. doi: 10.1093/plcell/koab133 (PMC8408442; doi:10.1093/plcell/koab133)
Supplement: koab133_Supplementary_Data [file koab133_supplementary_data.zip › tpc.00221.2021-s03.pdf]

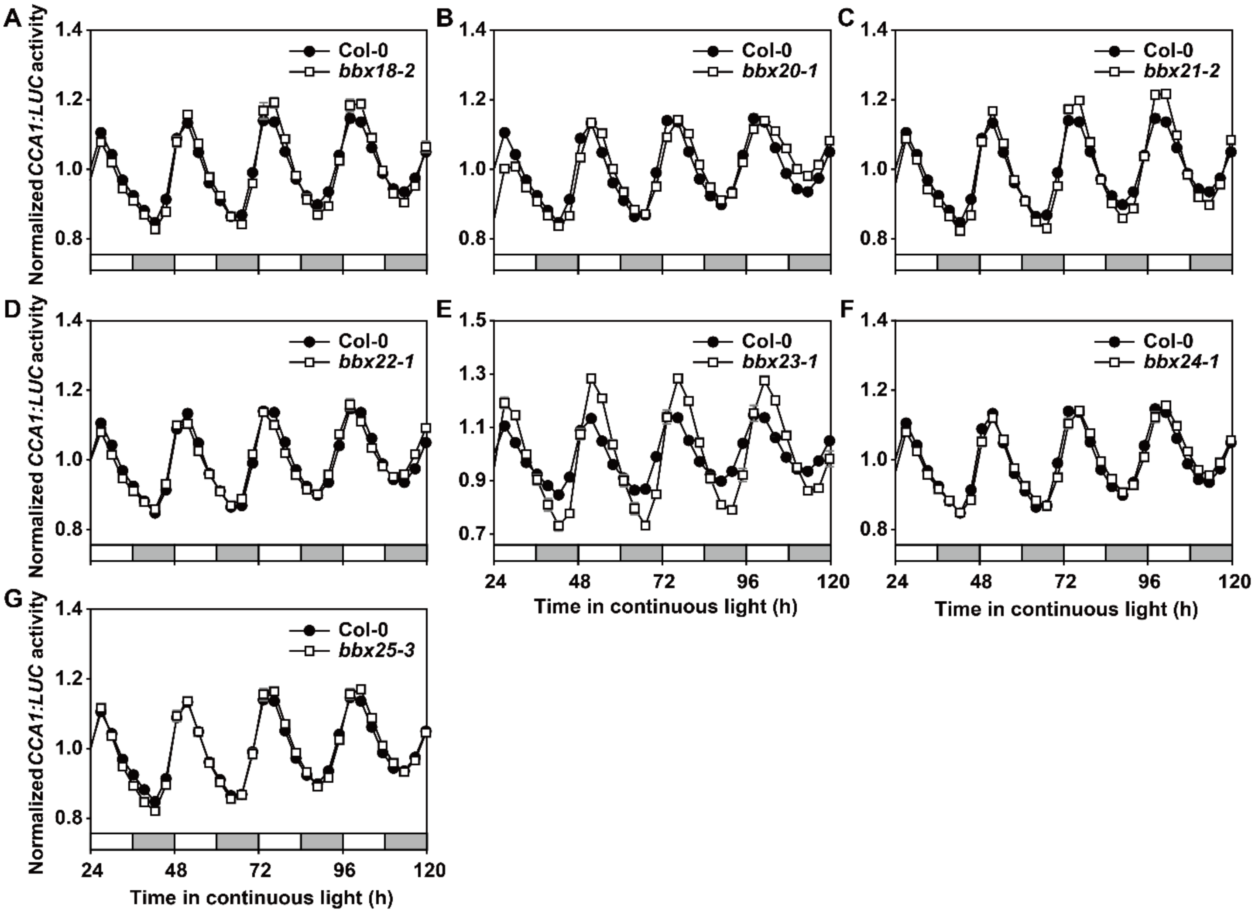

Supplemental Figure S1. Circadian rhythms of *CCA1:LUC* in the *BBX* subfamily IV gene mutation lines under free-running conditions. (Supports Figure 1.)

The luciferase reporter gene fusion *CCA1:LUC* was introduced into wild-type and *bbx18-2* (SALK_061956) (A), *bbx20-1* (CS878932) (B), *bbx21-2* (SALK_105390) (C), *bbx22-1* (SALK_105367) (D), *bbx23-1* (SALK_053389) (E), *bbx24-1* (SALK_067473) (F), and *bbx25-3* (CS2103310) (G) mutant plants. The transgenic seedlings were entrained under 12-h light:12-h dark (LD) cycles for 2 weeks before released to constant light (LL) at 22°C for 5 d. Circadian rhythms of *LUC* activity were captured with EM-CCD and normalized to the mean value over the time series. Data represent mean ± SE from three independent experiments; at least 24 individual seedlings were used for each analysis. Open bars indicate subjective day, and gray bars indicate subjective night.

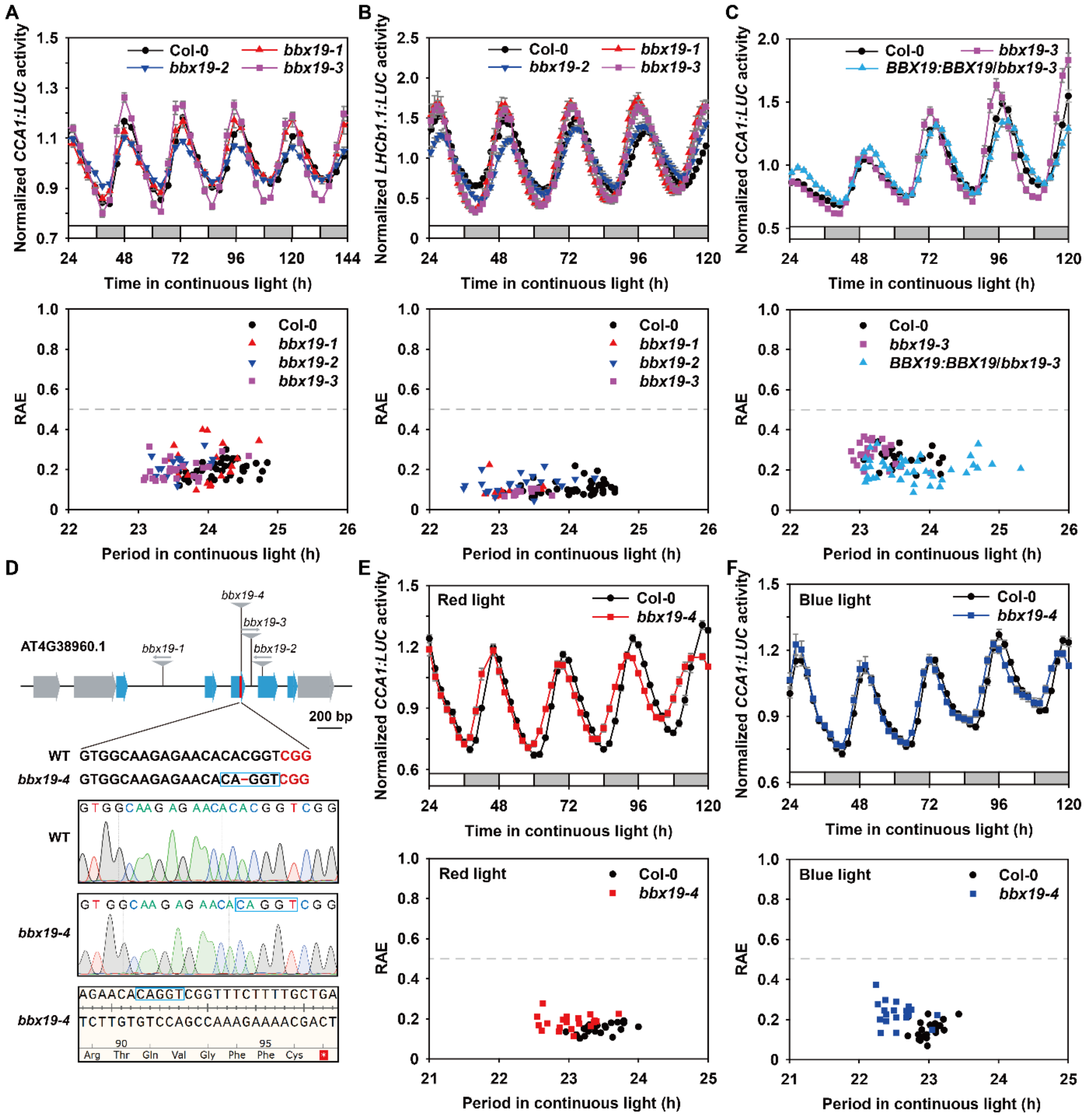

Supplemental Figure S2. Circadian rhythms in the *BBX19* mutation and complementation lines. (Supports Figure 1.)

(A-B) Circadian rhythms of *CCA1:LUC* (A) or *LHCB1.1:LUC* (B) in *bbx19-1* (SALK_088902), *bbx19-2* (SALK_087493), and *bbx19-3* (SALK_032997) T-DNA insertion individual lines were monitored under free-running conditions. *bbx19-1* and *bbx19-2* were previously published by Wang et al (2014) Plant Cell 26, 3589-3602.

(C) Circadian rhythm of *CCA1:LUC* in *BBX19:*BBX19/*bbx19-3* complementation line.

(D) Schematic diagram of the *BBX19* gene structure with the position of each T-DNA insertion mutation and CRISPR/Cas9-mediated genome editing mutation illustrated. The protein translation of the *bbx19-4* was terminated prematurely due to a single base deletion.

(E-F) Circadian rhythms of *CCA1:LUC* in *bbx19-4* mutant under constant red light or blue light conditions.

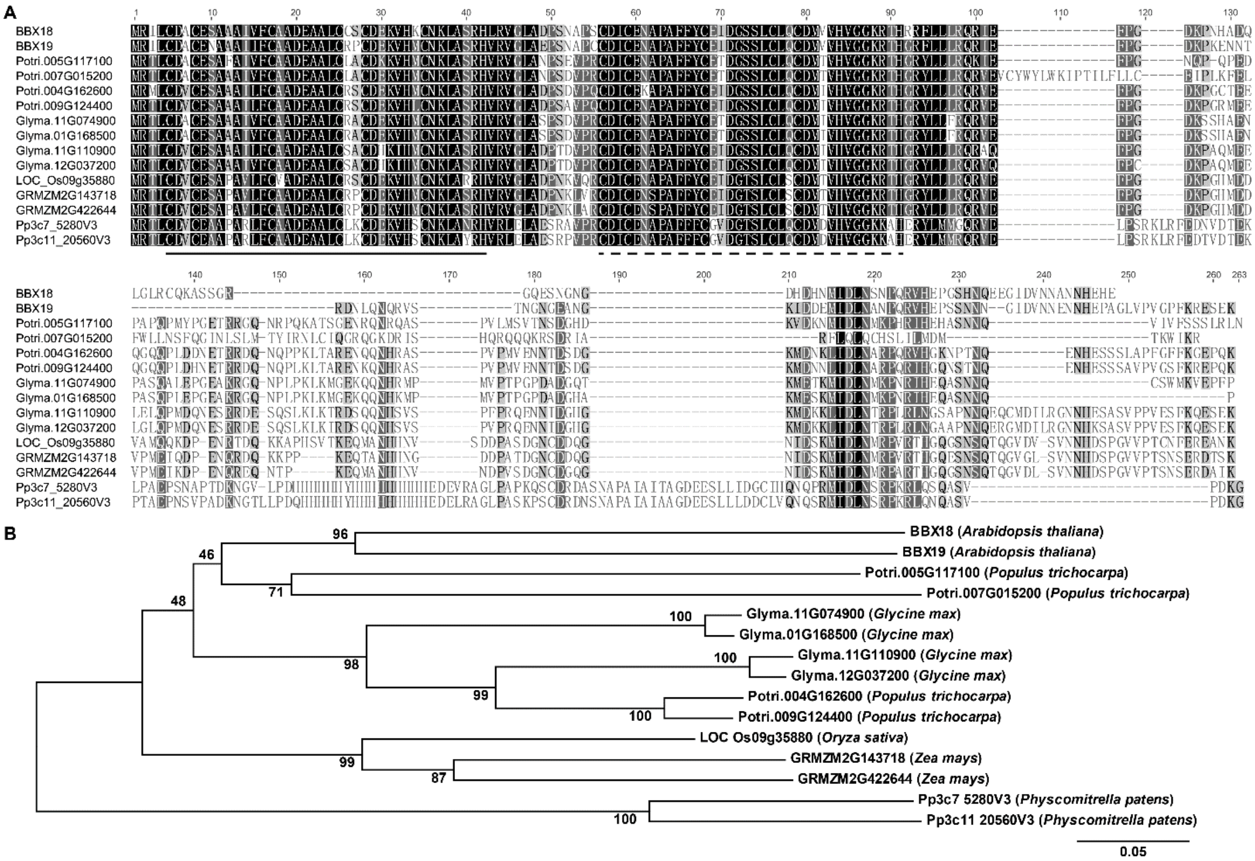

Supplemental Figure S3. Phylogenetic analysis of AtBBX18 and AtBBX19 orthologs in land plants. (Supports Figure 1.)

(A) Comparison of amino acid sequences of BBX18, BBX19 among a few representative species of higher plants including bryophyte (*Physcomitrella patens*), dicotyledonous legume family (*Glycine max*), dicotyledonous tree (*Populus trichocarpa*), monocotyledonous C3 plant (*Oryza sativa*) and monocotyledonous C4 plant (*Zea mays*). Two homologs in Arabidopsis, bryophyte or corn, four homologs in soybean or populous, and one homolog in rice were investigated using MUSCLE multiple sequence alignment with maximum 8 iterations, and the sequence data are available at Phytozome (https://phytozome.jgi.doe.gov/). Black solid and dashed underlines represent the two B-box domains, respectively. Highly conserved amino acids (threshold was set to 75%) are highlighted in shading.

(B) The phylogenetic radiant tree of BBX18, BBX19 orthologs. Evolutionary analyses were conducted with MEGA7, using Neighbor-Joining, a distance algorithmic method. The percentage of replicate trees in which the associated taxa clustered together in the bootstrap test (1000 replicates) is shown next to the branches. 0.05 indicates the genetic distance.

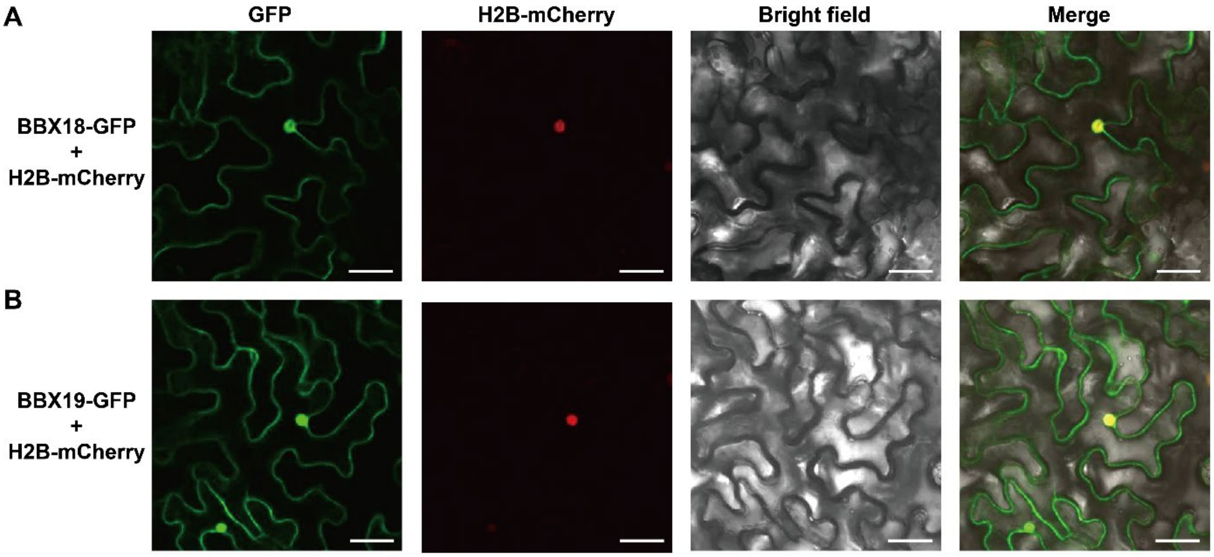

Supplemental Figure S4. Subcellular localization of BBX18 and BBX19. (Supports Figure 3.)

Confocal images of 5-week-old *Nicotiana benthamiana* leaves expressing GFP fused BBX18 **(A)** or BBX19 **(B)**. Construct of *CsVMV:BBX18-GFP* or *CsVMV:BBX19-GFP*, together with the *H2B-mCherry* plasmid, were co-injected into the leaf epidermal cells by *Agrobacterium*-mediated infiltration. Following 72 hours incubation, the fluorescence distribution in transfected leaves were observed under a two-photon confocal laser scanning microscope (CLSM). The H2B-mCherry was used as a nuclear marker. Scale bars indicate 20 μm.

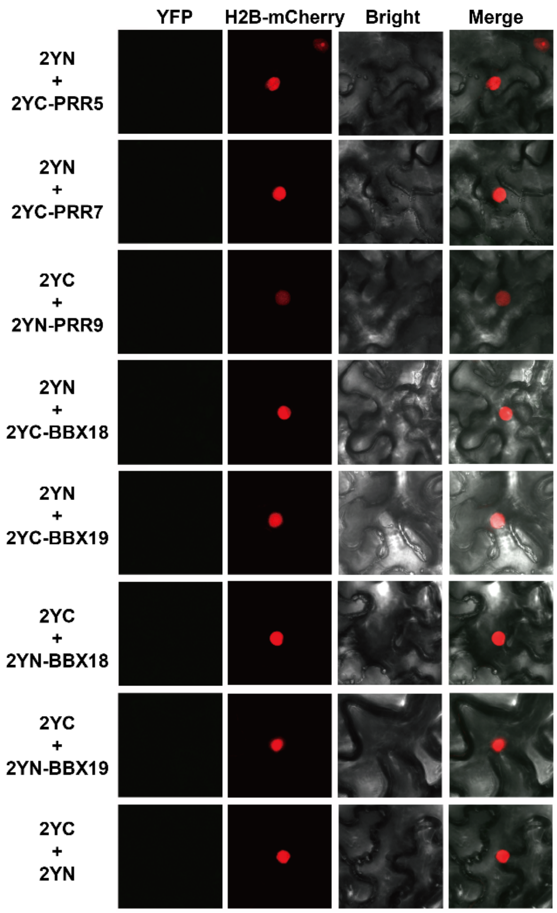

Supplemental Figure S5. Negative controls for BiFC assays. (Supports Figure 3.)

Each protein (PRR5, PRR7, PRR9, BBX18, or BBX19) was tagged with either the N- or C-terminal fragment of YFP as indicated. BiFC constructs together with the empty vector control were coinfiltrated into the *N. benthamiana* leaves. The fluorescent signal in epidermal cells was imaged at 48 hours after *A. tumefaciens*-mediated infiltration.

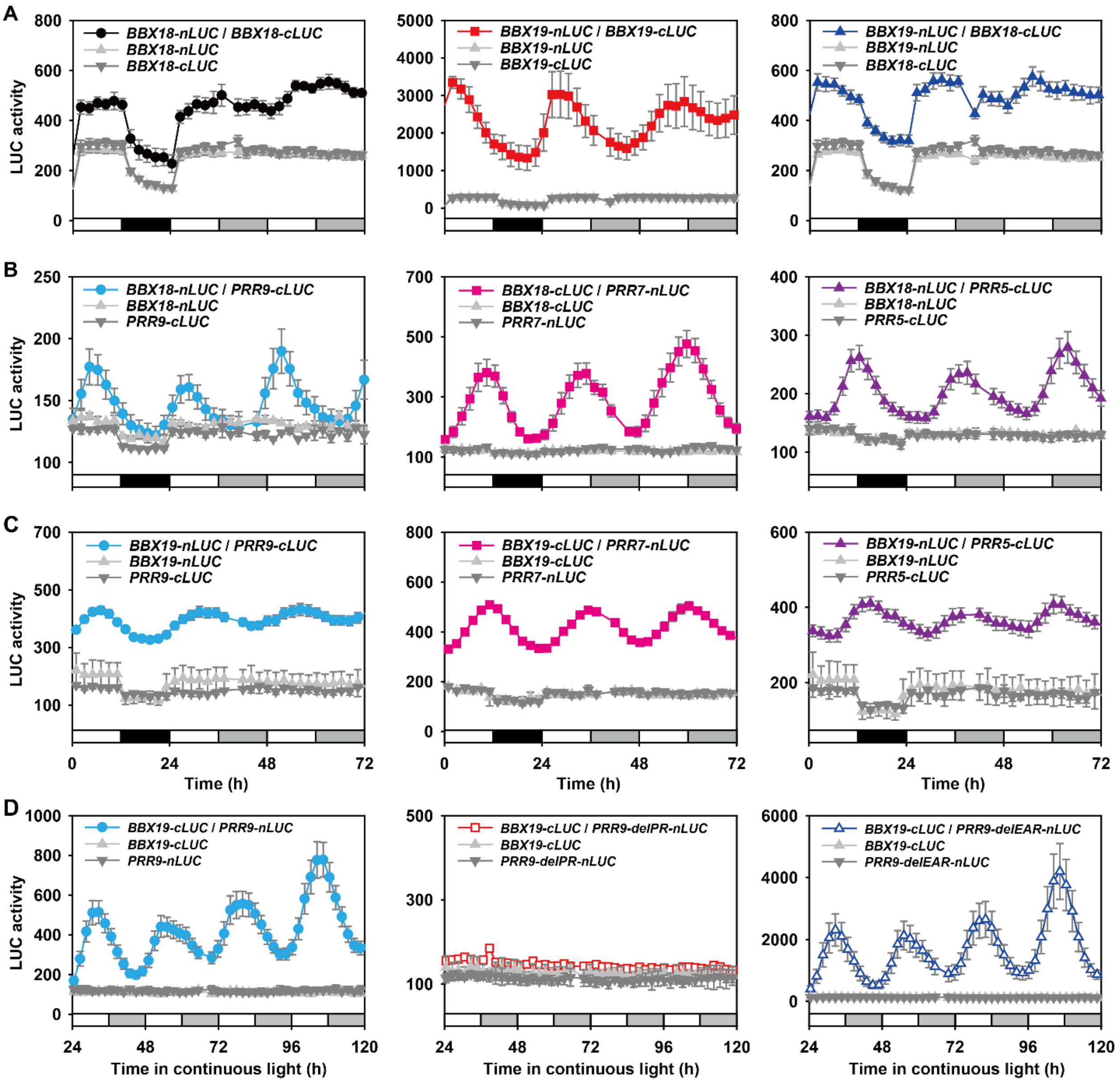

Supplemental Figure S6. LUC bioluminescence analysis showed dynamic protein-protein interactions between BBX19/18 and PRR proteins. (Supports Figure 4.)

The diurnal and circadian oscillations of the formation of each protein pair shown in Figure 4A-D. Each protein was fused to C- or N-terminal domain of LUC (cLUC or nLUC), then the transgenic Arabidopsis plants were generated by genetic cross. The LUC bioluminescence in F1 generation was continuously monitored for 72 or 120 hours with a TopCount™ luminometer. Data represent mean ± SE for three independent experiments. The white/black and white/gray bars indicate day/night and subjective day/night, respectively.

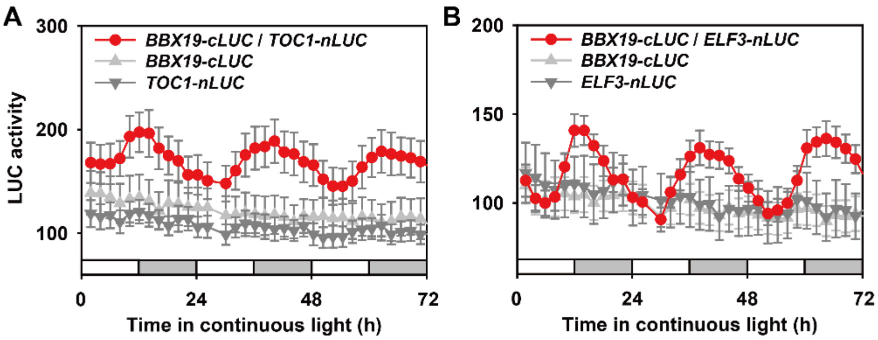

Supplemental Figure S7. Dynamic protein-protein interactions between BBX19 and TOC1, ELF3 proteins. (Supports Figures 3 and 4).

Each protein (BBX19, TOC1, or ELF3) was fused to C- or N-terminal domain of LUC (cLUC or nLUC). The transgenic Arabidopsis plants were generated by genetic cross. The LUC bioluminescence in F1 generation was continuously monitored for 72 hours with a TopCount^{TM} luminometer. Data represent mean ± SE for three independent experiments.

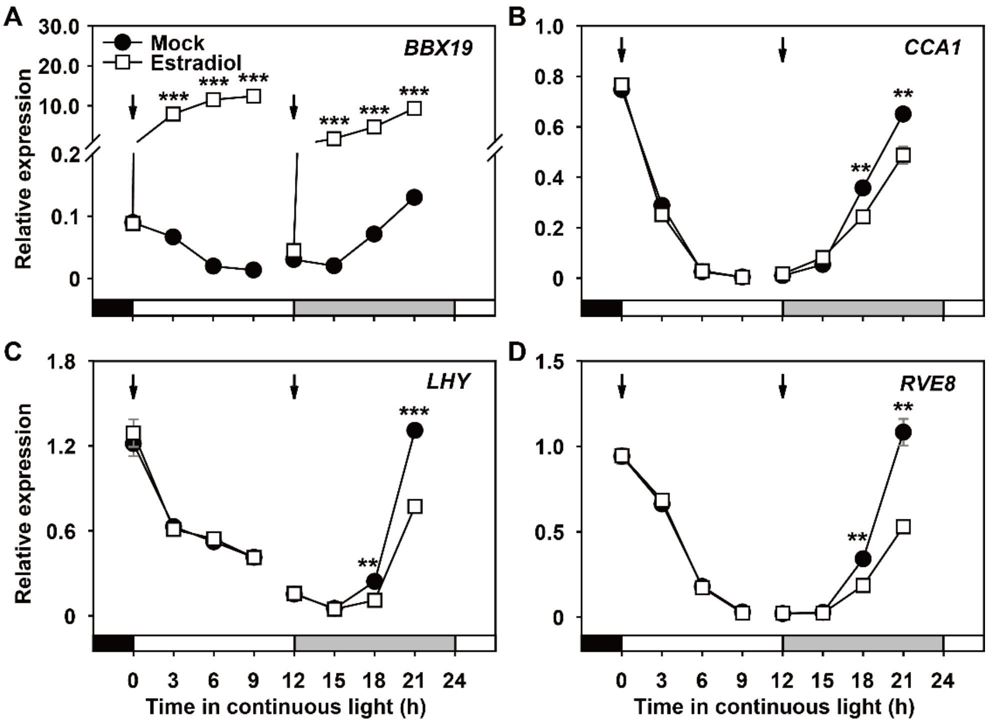

Supplemental Figure S8. Estradiol-induced *BBX19* expression at subjective night inhibited the transcript accumulation of *CCA1*, *LHY*, and *RVE8*. (Supports Figure 6.)

The biological replicate data of Figure 6G-J. The Arabidopsis seedlings carrying a *pER8-BBX19-YFP-HA* transgene were grown under 12:12 LD cycles for 10 days before *BBX19* were induced with β-estradiol during the day (ZT0) or night (ZT12) (A). Samples were then harvested for qRT-PCR analysis of the transcript accumulation of *CCA1* (B), *LHY* (C), and *RVE8* (D). Data shown mean ± SE of three technical replicates from one of three independent biological experiments, as shown in Figure 6G-J; *IPP2* was used as a normalization control; all experiments yielded congruent results.

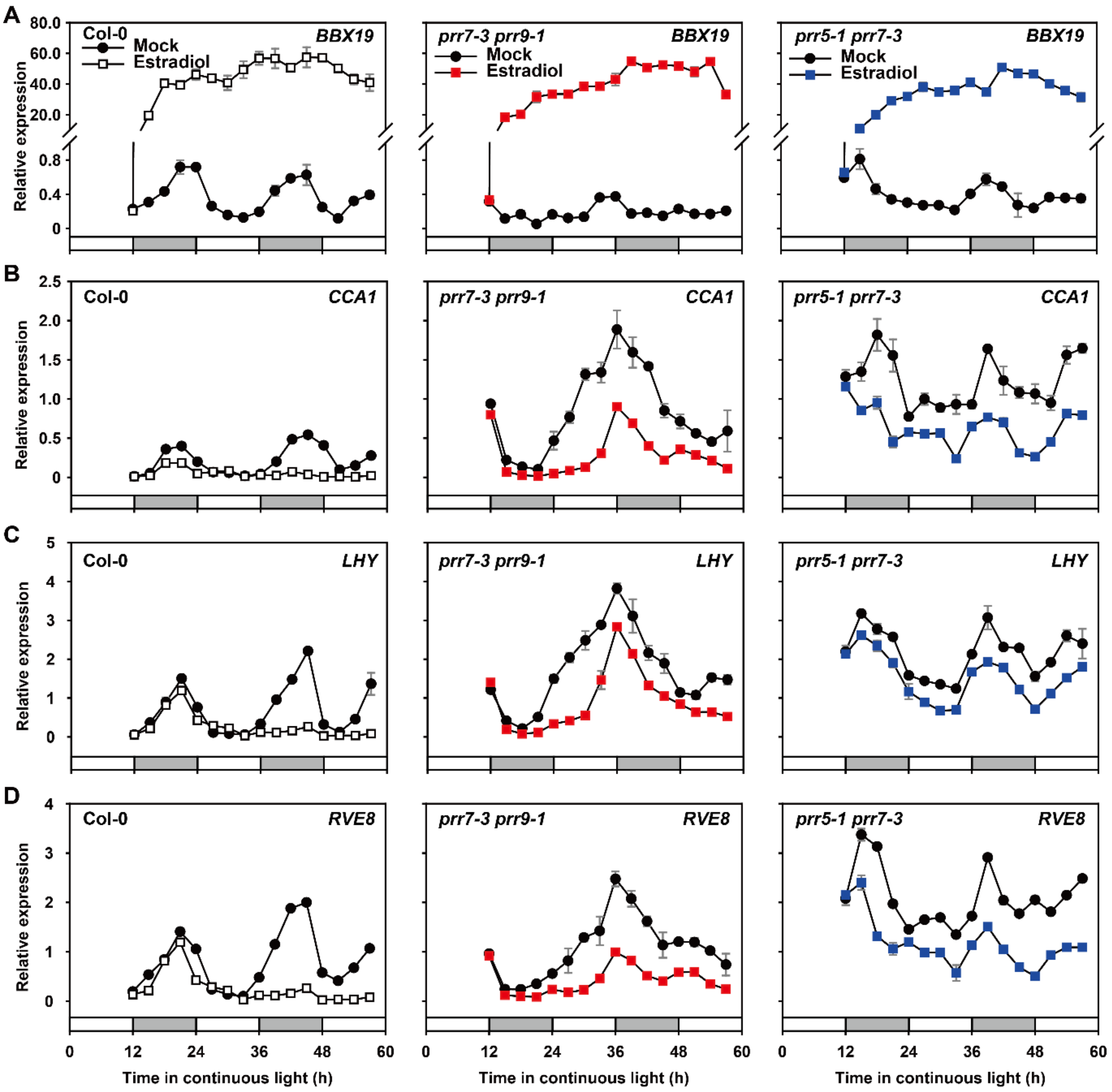

Supplemental Figure S9. BBX19 inhibits the accumulation of *CCA1*, *LHY*, and *RVE8* transcripts. (Supports Figure 7.)

The biological replicate data of Figure 7. The wild-type (Col-0), *prr7-3 prr9-1*, and *prr5-1 prr7-3* mutants containing *pER8-BBX19* were grown under 12:12 LD cycles for 10 days before *BBX19* were induced at ZT12 with β-estradiol (A). qRT-PCR analysis of the transcript accumulation of *CCA1* (B), *LHY* (C), and *RVE8* (D) in the Col-0, *prr7-3 prr9-1*, and *prr5-1 prr7-3* mutants. Data shown mean ± SE of three technical replicates from one of three independent biological experiments, as shown in Figure 7; *IPP2* was used as a normalization control; all experiments yielded congruent results. White or gray bars represent subjective day or subjective night, respectively.

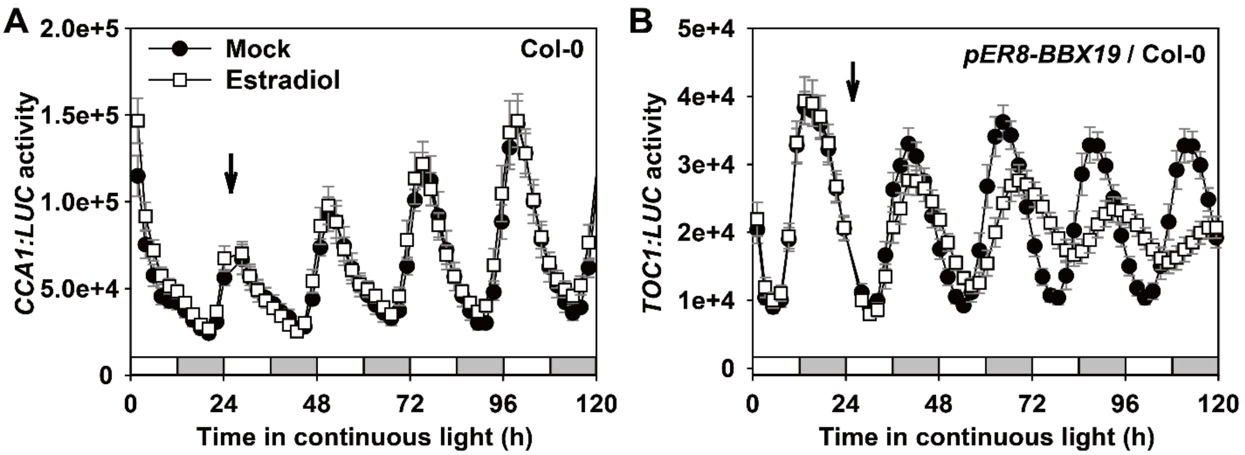

Supplemental Figure S10. *BBX19* overexpression leads to the reduced amplitude and lengthened period of *TOC1:LUC*. (Supports Figure 8A.)

(A) Seedlings of *CCA1:LUC*/Col-0 were treated with estradiol, which does not affect *CCA1:LUC* activity.

(B) Seedlings carrying both inducible *BBX19* and circadian reporter which were generated by cross *pER8-BBX19* with *TOC1:LUC*. seedlings were grown under 12:12 LD cycles for 7 days before transferred to LL at 22°C. Mock (filled circle) or estradiol (empty square) were applied to the plants at LL25. *LUC* activity was analyzed with TopCount™ luminometer. Data represent mean ± SE for three independent experiments. At least 24 individual seedlings were used for each analysis. White or gray bars represent subjective day or subjective night, respectively.

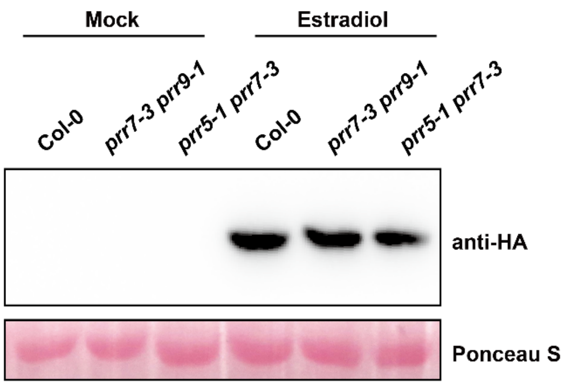

Supplemental Figure S11. Inducible expression of BBX19 protein in the *BBX19-YFP-HA* transgenic lines. (Supports Figure 8E.)

The wild type (Col-0), *prr7-3 prr9-1*, and *prr5-1 prr7-3* mutants containing *pER8-BBX19* were grown under 12:12 LD cycles for 14 days before BBX19 were induced at ZT12 with β-estradiol. Sampling was performed at ZT3 when BBX19 expression reached a significant peak. Total proteins were separated by SDS-PAGE and BBX19 proteins were confirmed by immunoblot with anti-HA.

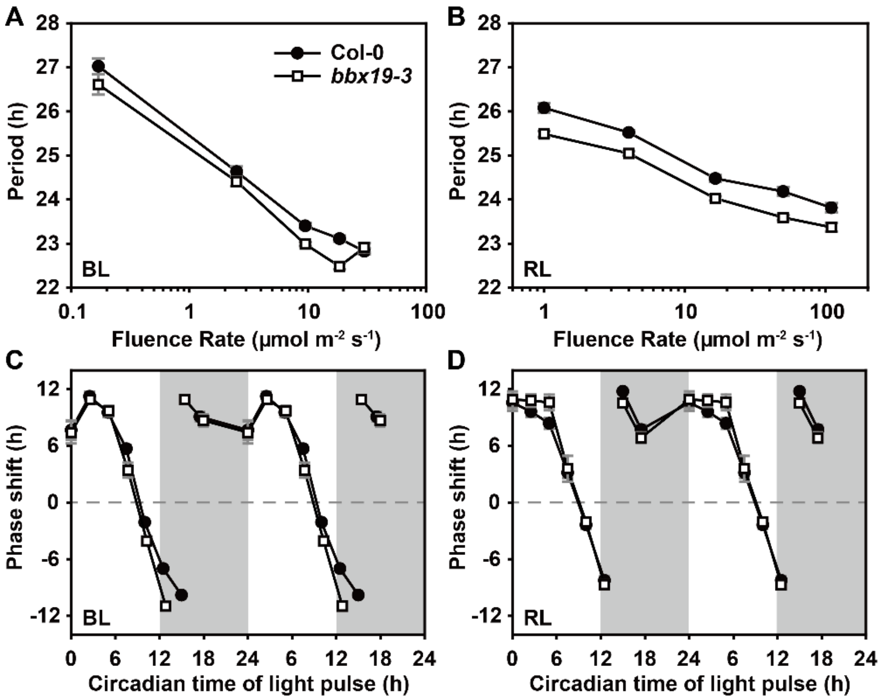

Supplemental Figure S12. Characteristics of circadian rhythms in response to environmental light cues. (Supports Discussion.)

(A-B) Fluence-rate response curve (FRC) of the circadian period to the increase of blue (A) or red (B) light intensity. After entraining for 7 days under 12:12 LD cycles, the bioluminescence trace of *CCA1:LUC* in wild-type (Col-0) and *bbx19-3* were measured every 3 h using a TopCount^{TM} luminometer under free-running conditions at the indicated fluence rate of blue (0.2, 2.5, 9.5, 18.5, or 30 µmol m^{-2} s^{-1}) (A) or red (1, 4, 16.5, 50, or 110 µmol m^{-2} s^{-1}) (B) light, respectively. The average period length of LL24-120 were estimated by BRASS 2.1.4 with FFT-NLLS method. Data shown mean ± SE from a representative experiment of three biological replicates. At least 24 individual seedlings were used for each analysis. X-axis values are plotted against the logarithm of the light fluence rate.

(C-D) Phase response curves (PRC) of circadian rhythmicity to discontinuous blue- or red-light pulses. After entrainment under 12-h L/12-h D cycles, wild-type (filled circle) and *bbx19-3* (empty square) seedlings were released into constant dark (DD) for one day. Samples were exposed to light at 3-h intervals over one complete circadian cycle (24 h) before returned to continuous dark for bioluminescence measurement the ensuing 5 days. Changes in acrophase (peak phase) are expressed relative to the acrophase in samples not exposed to light pulse and are plotted against the time of onset of the pulse. Advanced phase shifts are plotted as positive values, delayed phase shifts are plotted as negative values. PRC waveforms are double-plotted to emphasize the pacemaker sensitivity to light pulses.

Supplemental Table S1. Five genes of BBX subfamily IV, co-expressed with *CCA1* and *LHY* in multiple microarray- and RNAseq-based coexpression data sets in ATTED-II (http://atted.jp), were highly ranked in the co-expression list. (Supports Figure 1.)

Locus ID	Name	Description
At5g17300	*RVE1*	Homeodomain-like superfamily protein
At5g02840	*RVE4/LCL1*	LHY/CCA1-like 1, a homolog of the circadian rhythm regulator RVE8
At3g09600	*RVE8/LCL5*	LHY/CCA1-like 5
At3g54500	*LNK2*	NIGHT LIGHT-INDUCIBLE AND CLOCK-REGULATED 2
At3g12320	*LNK3*	NIGHT LIGHT-INDUCIBLE AND CLOCK-REGULATED 3
At5g06980	*LNK4*	NIGHT LIGHT-INDUCIBLE AND CLOCK-REGULATED 4
At5g15850	*COL1/BBX2*	CONSTANS-like 1
At3g02380	*COL2/BBX3*	CONSTANS-like 2
At2g21320	*BBX18*	B-box zinc finger family protein
At4g38960	*BBX19*	B-box type zinc finger family protein
At2g31380	*BBX25*	a B-box zinc finger protein that interacts with COP1
At3g17609	*HYH*	HY5-homolog
At5g24120	*SIGE*	Sigma factor E
At3g24170	*GR1*	Glutathione-disulfide reductase
At5g39660	*CDF2*	Cycling DOF factor 2
At1g69570	*CDF5*	Cycling DOF factor 5
At3g47420	*PS3*	Phosphate starvation-induced gene 3
At4g15430	*ERD*	ERD (early-responsive to dehydration stress) family protein
At4g20070	*AAH*	Allantoate amidohydrolase
At1g75100	*JAC1*	J-domain protein required for chloroplast accumulation response 1
At2g24540	*AFR*	Galactose oxidase/kelch repeat superfamily protein
At2g26800	*HGML*	3-HYDROXY-3-METHYLGLUTARYL-COA (HMGCOA) LYASE
At5g03555	*NCS1*	Permease, cytosine/purines, uracil, thiamine, allantoin family protein
At5g52570	*CHY2*	Beta-carotene hydroxylase 2
At1g32900	*GBSS1*	UDP-Glycosyltransferase superfamily protein
At2g41250	*HAD*	Haloacid dehalogenase-like hydrolase (HAD) superfamily protein
At2g47490	*NDT1*	NAD+ transporter 1
At5g64940	*ATH13*	ABC2 homolog 13
At5g62130	n/a	Per1-like family protein
At1g55960	n/a	Polyketide cyclase/dehydrase and lipid transport superfamily protein
At1g64500	n/a	Glutaredoxin family protein
At3g27350	n/a	Transcriptional regulator ATRX-like protein

Supplemental Table S2. Period length of *CCA1:LUC* circadian rhythms shown in Figure 1C-D.

**Genotype**	**Period (h, mean ± SE）**	**No.**	***P*-value**
WT (Col-0)	24.1 ± 0.1	24	
bbx18-2	24.2 ± 0.1	18	0.655
bbx19-3	23.5 ± 0.1	24	<0.001
bbx20-1	24.1 ± 0.1	21	0.964
bbx21-2	24.0 ± 0.1	24	0.132
bbx22-1	23.8 ± 0.1	19	0.014
bbx23-1	24.4 ± 0.1	15	0.031
bbx24-1	24.2 ± 0.1	20	0.331
bbx25-3	24.3 ± 0.1	23	0.046

Supplemental Table S3. Period length of circadian rhythms shown in Figure S2.

	**Period (h, mean ± SE）**	**No.**	***P*-value**
**Figure S2A**			
Col-0	24.2 ± 0.1	40	
bbx19-1	24.0 ± 0.1	20	0.033 (vs. Col-0)
bbx19-2	23.6 ± 0.1	19	<0.001 (vs. Col-0)
bbx19-3	23.6 ± 0.1	24	<0.001 (vs. Col-0)
**Figure S2B**			
Col-0	24.0 ± 0.1	42	
bbx19-1	23.1 ± 0.1	12	<0.001 (vs. Col-0)
bbx19-2	23.3 ± 0.1	23	<0.001 (vs. Col-0)
bbx19-3	23.4 ± 0.1	10	<0.001 (vs. Col-0)
**Figure S2C**			
Col-0	23.6 ± 0.1	23	
bbx19-3	23.2 ± 0.1	24	<0.001 (vs. Col-0)
BBX19:BBX19/bbx19-3	23.7 ± 0.1	44	0.513 (vs. Col-0)<br><0.001 (vs. *bbx19-3*)
**Figure S2E**			
Col-0	23.4 ± 0.1	22	
bbx19-4	23.0 ± 0.1	20	<0.001 (vs. Col-0)
**Figure S2F**			
Col-0	23.0 ± 0.0	20	
bbx19-4	22.6 ± 0.1	20	<0.001 (vs. Col-0)

Supplemental Table S4. Period length of *CCA1:LUC* circadian rhythms shown in Figure 2A-D.

	Period (h, mean ± SE)	RAE	No.	*P*-value
**Figure 2A-B**				
WT (Col-0)	23.3 ± 0.1	0.16 ± 0.01	31	
BBX18:BBX18/Col-0	24.5 ± 0.1	0.26 ± 0.01	33	<0.001 (vs. Col-0)
BBX19:BBX19/Col-0	24.7 ± 0.1	0.29 ± 0.01	22	<0.001 (vs. Col-0)
**Figure 2C-D**				
WT (Col-0)	23.7 ± 0.1	0.25 ± 0.02	21	
bbx19-3	23.1 ± 0.1	0.26 ± 0.01	21	<0.001 (vs. Col-0)
bbx18 bbx19-3	23.0 ± 0.1	0.23 ± 0.01	35	<0.001 (vs. Col-0)<br>0.083 (vs. *bbx19-3*)

Supplemental Table S5. Period length of *CCA1:LUC* circadian rhythms shown in Figure 5A-C.

	Period (h, mean ± SE)	RAE	No.	*P*-value
WT (Col-0)	24.1 ± 0.1	0.14 ± 0.01	24	
bbx19-3	23.6 ± 0.1	0.14 ± 0.01	24	<0.001 (vs. Col-0)
prr9-1	25.0 ± 0.1	0.19 ± 0.01	14	
bbx19-3 prr9-1	24.2 ± 0.1	0.19 ± 0.01	13	<0.001 (vs. *prr9-1*)<br><0.001 (vs. *bbx19-3*)
prr7-3	25.5 ± 0.1	0.18 ± 0.01	23	
bbx19-3 prr7-3	24.4 ± 0.2	0.19 ± 0.01	17	<0.001 (vs. *prr7-3*)<br><0.001 (vs. *bbx19-3*)
prr5-1	23.4 ± 0.1	0.13 ± 0.01	18	
bbx19-3 prr5-1	22.8 ± 0.1	0.16 ± 0.01	15	<0.001 (vs. *prr5-1*)<br><0.001 (vs. *bbx19-3*)
prr5-1 7-3	17.8 ± 0.1	0.31 ± 0.02	22	
bbx19-3 prr5-1 7-3	18.2 ± 0.1	0.32 ± 0.02	25	0.008 (vs. *prr5-1 prr7-3*)<br><0.001 (vs. *bbx19-3*)

Supplemental Table S6. Period length of *CCA1:LUC* circadian rhythms shown in Figure 5D-F.

	Period (h, mean ± SE)	RAE	No.	*P*-value
WT (Col-0)	23.4 ± 0.1	0.24 ± 0.01	24	
bbx19-3	22.5 ± 0.1	0.20 ± 0.01	24	<0.001 (vs. Col-0)
cca1-1	22.1 ± 0.1	0.27 ± 0.02	24	
bbx19-3 cca1-1	21.1 ± 0.1	0.28 ± 0.02	12	<0.001 (vs. *cca1-1*)
lhy-20	21.5 ± 0.1	0.27 ± 0.02	24	
bbx19-1 lhy-20	21.2 ± 0.1	0.28 ± 0.01	24	<0.001 (vs. *lhy-20*)
cca1-1 lhy-20	18.8 ± 0.1	0.39 ± 0.02	21	
bbx19-3 cca1-1 lhy-20	17.7 ± 0.1	0.40 ± 0.02	24	<0.001 (vs. *cca1-1 lhy-20*)
toc1-101	20.0 ± 0.1	0.34 ± 0.01	24	
bbx19-3 toc1-101	19.6 ± 0.1	0.30 ± 0.01	24	<0.001 (vs. *toc1-101*)

Supplemental Table S7. Oligonucleotides (shown 5' to 3’) used in this study.

**For Constructs**	
BBX18-F	CTGGGATTTAAACAAGGAGCGGTAG
BBX18-R	GATCACTTAAACCGAGCCAAGAAGG
BBX18-SfiI-F	GGCCTTGACGGCCTTCATGCTCGTGATTGTTTGC
BBX18-SfiI-R	GGCCTCATGGGCCTAGTGATCGCAGATACACAAG
BBX18-CDS-TOPO-F	CACCATGCGAATTTTGTGTGATGC
BBX18-CDS-TOPO-R	TTCATGCTCGTGATTGTTTG
BBX18-CDS-Y2H-F	CCGGAATTCATGCGAATTTTGTGTGATGC
BBX18-CDS-Y2H-R	CGCGTCGACTTCATGCTCGTGATTGTTTG
BBX18-CDS-SmaI-F	TCCCCCGGGATGCGAATTTTGTGTGATGC
BBX18-CDS-KpnI-R	CGGGGTACCCTATTCATGCTCGTGATTGTTTG
BBX18-2YC/N-PacI-F	ATTTACGAACGATAGTTAATTAAATGCGAATTTTGTGTGATGC
BBX18-2YC/N-SpeI-R	ACTGCCACCTCCTCCACTAGTTTCATGCTCGTGATTGTTTG
BBX18-KpnI-GFP-1300-F	AATTCTGCAGTCGACGGTACCATGCGAATTTTGTGTGATGC
BBX18-XmaI-GFP-1300-R	TTCTCCTTTACTCATCCCGGGCTTCATGCTCGTGATTGTTTG
BBX18-BamHI-cFlag-F	CGGGATCCATGCGAATTTTGTGTGATGC
BBX18-HindIII-cFlag-R	CCCAAGCTTTTCATGCTCGTGATTGTTTG
BBX19-F	GCGTCGACCTGTAATGGTCACCCACTATGC
BBX19-R	GGGGTACCGCTTACTTTGCGTTAGCTACTCC

BBX19-SfiI-F	GGCCTTGACGGCCCTTCTCAGACTCTCGTTTAAAGG
BBX19-SfiI-R	GGCCTCATGGGCCTGATAAGGTGAGAGAGAGAGAG
BBX19-CDS-TOPO-F	CACCATGCGGATTTTGTGCGATG
BBX19-CDS-TOPO-R	CTTCTCAGACTCTCGTTTAAAGGGTC
BBX19-CDS-Y2H-F	CCGGAATTCATGCGGATTTTGTGCGATG
BBX19-CDS-Y2H-R	CGCGTCGACCTTCTCAGACTCTCGTTTAAAGGGTC
BBX19-CDS-SmaI-F	TCCCCCGGGATGCGGATTTTGTGCGATG
BBX19-CDS-KpnI-R	CGGGGTACCTCACTTCTCAGACTCTCGTTTAAAGG
BBX19-2YC/N-PacI-F	ATTTACGAACGATAGTTAATTAAATGCGGATTTTGTGCGATG
BBX19-2YC/N-SpeI-R	ACTGCCACCTCCTCCACTAGTCTTCTCAGACTCTCGTTTAAAGGG
BBX19-KpnI-GFP-1300-F	AATTCTGCAGTCGACGGTACCATGCGGATTTTGTGCGATG
BBX19-XmaI-GFP-1300-R	TTCTCCTTTACTCATCCCGGGCCTTCTCAGACTCTCGTTTAAAGGG
BBX19-BamHI-cFlag-F	CGGGATCCATGCGGATTTTGTGCGATG
BBX19-HindIII-cFlag-R	CCCAAGCTTCTTCTCAGACTCTCGTTTAAAGGG
PRR9-2YC/N-PacI-F	ATTTACGAACGATAGTTAATTAAATGGGGGAGATTGTGGTTTT
PRR9-2YC/N-SpeI-R	ACTGCCACCTCCTCCACTAGTTGATTTTGTAGACGCGTCTG
PRR9-KpnI-HA-1300-F	AATTCTGCAGTCGACGGTACCATGGGGGAGATTGTGGTTTT
PRR9-XmaI-HA-1300-R	TAACCTGCCATGGATCCCGGGGTTGATTTTGTAGACGCGTCTG
PRR7-2YC/N-PacI-F	ATTTACGAACGATAGTTAATTAAATGAATGCTAATGAGGAGGG
PRR7-2YC/N-SpeI-R	ACTGCCACCTCCTCCACTAGTGCTATCCTCAATGTTTTTTA
PRR7-KpnI-HA-1300-F	AATTCTGCAGTCGACGGTACCATGAATGCTAATGAGGAGGG
PRR7-XmaI-HA-1300-R	TAACCTGCCATGGATCCCGGGGTGCTATCCTCAATGTTTTTTA
PRR5-2YC/N-PacI-F	ATTTACGAACGATAGTTAATTAAATGACTAGTAGCGAGGAAGT
PRR5-2YC/N-SpeI-R	ACTGCCACCTCCTCCACTAGTTGGAGCTTGTGTGGATT
PRR5-KpnI-HA-1300-F	AATTCTGCAGTCGACGGTACCATGACTAGTAGCGAGGAAGT
PRR5-XmaI-HA-1300-R	TAACCTGCCATGGATCCCGGGGTTGGAGCTTGTGTGGATT
PRR9-LOCUS-F	AAGCGGCCGCGCAGCAAAGATGGTAATGAAGC
PRR9-LOCUS-R	AAGCGGCCGCGAAGAAGCGTTGAGACTCTC
PRR9-SF	GTCGGCCTTGACGGCCTGATTTTGTAGACGCGTCTG
PRR9-SR	GACGGCCTCATGGGCCTGAAGAGCTCTTTCAGTTTTTCC
PRR7-LOCUS-F	ACGCGGCGGCCGCGAGAGTTCTGGT
PRR7-LOCUS-R	AAGCGGCCGCTCGATTGTGTTCGATGATCTC
PRR7-SR	GACGGCCTCATGGGCCTAACGGAAAATCAACAAAACCCAC

PRR5-LOCUS-F	GTGGGATCCATGTGTTGTTGGCTATTGTAAAC
PRR5-LOCUS-R	GAGCGGCCGCTATTTTTGTGTTAGTGGATAC
PRR5-SF	GTCGGCCTTGACGGCCTGGAGCTTGTGTGGATTGGACTTGAC
PRR5-SR	GACGGCCTCATGGGCCTAGTGATCCAACAAATTGATATATT
PRR7-SR	GACGGCCTCATGGGCCTAACGGAAAATCAACAAAACCCAC
Luc-SF	GACGGCCGTCAAGGCCACATGGTCACCGACGCCAAAAAC
Luc-SR	GTCGGCCCATGAGGCCTTACACGGCGATCTTTCCGCCCTTC
cLuc-SF	CGGCCGTCAAGGCCACATGTCCGGTTATGTAAACAATC
nLuc-SR	CGGCCCATGAGGCCTTATCCATCCTTGTCAATCAAGGCGT
PRR9-DelREC-R	ATTTACAGTCAGTCTTCTAAGTACAGTTTTAGGC
PRR9-DelREC-F	AGAAGACTGACTGTAAATTTTTTATTTCCTT
PRR9-DelEAR-F	TTTGAGAACCAAGATGAAAGCAAGCATCAAAAG
PRR9-DelEAR-R	TTCATCTTGGTTCTCAAAACTCTTGTCTTTATAAATAC
bbx19-4-F (crispr)	GTGGCAAGAGAACACACGGTGTTTTAGAGCTAGAAATAGCAAGTTA
bbx19-4-R (crispr)	ACCGTGTGTTCTCTTGCCACAATCACTACTTCGACTCTAGC
pER8-PgRNA-F	CCTGTCAAACACTGATAGTTTGTCCCAGGATTAGAATGATTAGG
NEW-pHDE-R	CAAGCTTCACTTCACTAGTTTCCATCAGAGGTGTAACGGAATG
bbx19-4-F (PCR)	CCTTCTTTTACTGTGAGATAGACG
bbx19-4-R (PCR)	TCACTTCTCAGACTCTCGTTTAAA
**For Genotyping**	
bbx19-T-DNA-F	TCTGTCATGTACATTGCAGGG
bbx19-T-DNA-R	AAGGGTCCAACTGGTACAAGG
LBb1.3	ATTTTGCCGATTTCGGAAC
**For qRT-PCR**	
BBX19-RT-F	GGATAAGCCTAAAGAAAACAATACGAG
BBX19-RT-R	CGTTATTTACATCAATCCCGTTG
CCA1-RT-F	CCAGATAAGAAGTCACGCTCAGAA
CCA1-RT-R	GTCTAGCGCTTGACCCATAGCT
LHY-RT-F	GACTCAAACACTGCCCAGAAGA
LHY-RT-R	CGTCACTCCCTGAAGGTGTATTT
RVE8-RT-F	GGGAAGCTCAAGCCGAACAGTATC

RVE8-RT-R	GGCCTCTCGTTTCAGGATCAAAGA
PIL1-RT-F	GGAAGCAAAACCCTTAGCATCAT
PIL1-RT-R	TCCATATAATCTTCATCTTTTAATTTTGGTTTA
IAA29-RT-F	ATCACCATCATTGCCCGTAT
IAA29-RT-R	ATTGCCACACCATCCATCTT
XTR7-RT-F	CGGCTTGCACAGCCTCTT
XTR7-RT-R	TCGGTTGCCACTTGCAATT
AtHB2-RT-F	GCTGAAGCAAACGGAGGTAG
AtHB2-RT-R	TTTGTAGCCGACGGTTCTCT
IPP2-RT-F	GTATGAGTTGCTTCTCCAGCAAAG
IPP2 -RT-R	GAGGATGGCTGCAACAAGTGT
PIF4-RT-F	TCAGATGCAGCCGATGGAG
PIF4-RT-R	GTTGTTGACTTTGCTGTCCCG
**For ChIP-qPCR**	
ChIP-*UBQ*-F	TCCAGGACAAGGAAGGTATTCCTCCG
ChIP-*UBQ*-R	CCACCAAAGTTTTACATGAAACGAA
ChIP-*CCA1*-P1-F	GGAGCAGCTGAATCAAAGGAGGA
ChIP-*CCA1*-P1-R	GAAGGACGTGGCCTTTGTGTTAG
ChIP-*CCA1*-P2-F	GTTGAATAACGATATGGGCCTTATGC
ChIP-*CCA1*-P2-R	CACGCGCTATTCTCACACTCT
ChIP-*CCA1*-P3-F	CGCGTTCGATTGTTGGTGAAG
ChIP-*CCA1*-P3-R	GATCTAGTGGGACCTACTTAAGATCG
ChIP-*CCA1*-P4-F	CCTTTCAGGTAGTCCCGGAACTC
ChIP-*CCA1*-P4-R	AGTGGCTGAAGAGATGATTCAGCT
ChIP-*CCA1*-P5-F	CTCAAGCTTCCACATGAGACTCT
ChIP-*CCA1*-P5-R	CAGGAAGACTATGGACAAGGAAAC
ChIP-*LHY*-P1-F	GCAAGTTGACCAAAGTTCTCGATG
ChIP-*LHY*-P1-R	ACTGCTCGGGGAACTTGC
ChIP-*LHY*-P2-F	CCAGATCCAGCATAGCCAATACG
ChIP-*LHY*-P2-R	AGTGTGGTGGTCCACAATTGC
ChIP-*LHY*-P3-F	GCTTCTGGCTCGTAGAGAAGC
ChIP-*LHY*-P3-R	GAGGCTGGAACAGCACCAAG
ChIP-*LHY*-P4-F	CTTGTCTCCTCCATGGCTACTCTC
ChIP-*LHY*-P4-R	GTTTCTCAGCAGCCAAACAGAG

ChIP-*LHY*-P5-F	GCTTCTACATGACAGACTTGGAGG
ChIP-*LHY*-P5-R	ATGGTACAGAACCTGACATGACCA
ChIP-*RVE8*-P1-F	GGAAAGATGTGCGGCCATTACAC
ChIP-*RVE8*-P1-R	CTTCGTAATTCCCGCGTGGATC
ChIP-*RVE8*-P2-F	ATAACGACATGGGCGGCAAG
ChIP-*RVE8*-P2-R	CTAAGCACCAAATACGCATCCTAG
ChIP-*RVE8*-P3-F	CTACAAGTCTACACGTGCTTCAAGC
ChIP-*RVE8*-P3-R	GTTGGGATGTGTATGTGGAGTAGG
ChIP-*RVE8*-P4-F	GCACCAGTTCAGGCTTCTTG
ChIP-*RVE8*-P4-R	GTGATTCCAGTGACGTATAACACCA
