# Supplementary material for: BBX19 fine-tunes the circadian rhythm by interacting with PSEUDO-RESPONSE REGULATOR proteins to facilitate their repressive effect on morning-phased clock genes
Source: Plant Cell. 2021 May 14;33(8):2602–17. doi: 10.1093/plcell/koab133 (PMC8408442; doi:10.1093/plcell/koab133)
Supplement: koab133_Supplementary_Data [file koab133_supplementary_data.zip › tpc.00221.2021-s04.pdf]

>BBX18_Arabidopsis_thaliana
MRILCDACESAAAIVFCAADEAALCCSCDEKVHKCNKLASRHLRVGLADPSNAPSCDICENAPAFFYC
EIDGSSLCLQCDMVVHVGGKRTHRRFLLLRQRIEFPGDKPNHAD---------QLG-LRCQKAS-----
-------------SGRGQESNGNGDHDHNMIDLNSNPQRVHEPGSHNQEEGIDVNNANNHEHE---
----
>BBX19_Arabidopsis_thaliana
MRILCDACENAAAIIFCAADEAALCRPCDEKVHMCNKLASRHVRVGLAEPSNAPCCDICENAPAFFY
CEIDGSSLCLQCDMVVHVGGKRTHGRFLLLRQRIEFPGDKPKENN---------TRDNLQNQRVS---
---------------TNGNGEANG--KIDDEMIDLNANPQRVHEPSSNNN--
GIDVNNENNHEPAGLVPVGPFKRESEK-------
>Glyma.01G168500_Glycine_max
MRTLCDACESAAAIVFCAADEAALCRACDEKVHMCNKLASRHVRVGLASPSDVPRCDICENAPAFFY
CETDGSSLCLQCDMIVHVGGKRTHGRYLLFRQRVEFPGDKSSHAENPASQ--
PLEPGEAKRGQNPLPKLKMGEKQQNHKMPMVPTPGPDADGHAKMESKMIDLNMKPNRIHEQASN
NQP-----------------------------------
>Glyma.11G074900_Glycine_max
MRTLCDACESAAAIVFCAADEAALCRACDEKVHMCNKLASRHVRVGLASPSDVPRCDICENAPAFFY
CETDGSSLCLQCDMIVHVGGKRTHGRYLLFRQRVEFPGDKSSHAENPASQ--
ALEPGEAKRGQNPLPKLKMGEKQQNHRMPMVPTPGPDADGQTKMETKMIDLNMKPNRIHEQAS
NNQC---------SWMKVEPFP-----------------
>Glyma.11G110900_Glycine_max
MRTLCDVCESAAAILFCAADEAALCSACDHKIHMCNKLASRHVRVGLADPTDVPRCDICENAPAFFY
CEIDGSSLCLQCDMIVHVGGKRTHGRYLLLRQRAQFPGDKPAQMEELELQ--
PMDQNESRRDESQSLKLKTRDSQQNHSVSPFPRQENNIDGHGKMDKKLIDLNTRPLRLNGSAPNN
QEQCMDILRGNNHESASVPPVESFKQESEK-------
>Glyma.12G037200_Glycine_max
MRTLCDVCESAAAIVFCAADEAALCSACDHKIHMCNKLASRHVRVGLADPTDVPRCDICENAPAFFY
CEIDGSSLCLQCDMIVHVGGKRTHGRYLLLRQRVQFPCDKPAQMEELGLQ--
PMDQNESRRDESQSLKLKIRDSQQNHSVSPVPRQENNIDGHGKMDKKLIDLNTRPLRLNGAAPNN
QERGMDILRGNNHKSASVPPVESFKQESEK-------
>GRMZM2G143718_Zea_mays
MRTICDVCESAPAVLFCAADEAALCRPCDEKVHMCNKLASRHVRVGLADPNKLVRCDICENSPAFFY
CEIDGTSLCLSCDMTVHVGGKRTHGRYLLLRQRVEFPGDKPGHMDDVPME--
IQDPENQRDQKKPP----KEQTANHHNG-
DDPATDGNCDDQGNIDSKMIDLNMRPVRTHGQESNSQTQGVGLS-
VNNHDSPGVVPTSNSERDTSK-------
>GRMZM2G422644_Zea_mays
MRTICDVCESAPAVLFCAADEAALCRPCDEKVHMCNKLASRHVRVGLADPNKLARCDICENSPAFFY
CEIDGTSLCLSCDMTVHVGGKRTHGRYLLLRQRVEFPGDKPGHMDDVPME--IKDPENQREQNTP-
----KEQMANHHNV-NDPVSDGNCDGQGNIDSKMIDLNMRPARTHGQGSNSQTQGVDLS-
VNNHDSPGVVPTSNSERDAIK-------
>LOC_Os09g35880_Oryza_sativa
MRTICDVCESAPAVLFCVADEAALCRSCDEKVHMCNKLARRHVRVGLADPNKVQRCDICENAPAFF
YCEIDGTSLCLSCDMTVHVGGKRTHGRYLLLRQRVEFPGDKPGHMDDVAMQ--

QKDPENRTDQKKAPHSVTKEQMANHHNVSDDPASDGNCDDQGNIDSKMIDLNMRPVRTHGQG
SNSQTQGVDVS-VNNHDSPGVVPTCNFEREANK-------
>Potri.004G162600_Populus_trichocarpa
MRMLCDVCESAAAILFCAADEAALCRSCDEKVHMCNKLASRHVRVGLADPSDVPQCDICEKAPAFF
YCEIDGSSLCLQCDMIVHVGGKRTHGRYLLLRQRVEFPGDKPGCTEEQGQQ--
PLDDNETRRDQNQPPKLTARENQQNHRASPVPMVENNTDSDGKMDNKLIDLNARPQRVHGKNP
TNQE---------NHESSSLAPFGFFKGEPQK-------
>Potri.005G117100_Populus_trichocarpa
MRTLCDACESAFAIVFCAADEAALCLACDKKVHMCNKLASRHVRVGLANPSEVPRCDICENAPAFFY
CETDGSSLCLQCDMTVHVGGKRTHGRYLLLRQKIEFPGNQP-QPEDPAPQ--
PMYPGETRRGQNRPQKATSGENRQNRQASPVLMSVTNSDGHDKVDKNMIDLNMKPHRIHEHAS
NNQV---------IVFSSSLRLN----------------
>Potri.007G015200_Populus_trichocarpa
MRTLCDACESAAAIVFCAADEAALCLACDEKVHMCNKLASRHVRVGLANPSDVPRCDICENAPAFF
YCETDGSSLCLQCDMTVHVGGKRTHGRYLLLRQRVEVCYWYLWKIPTILFL--LCEIPLKFELFWLLN--
--------------SFQGINLSLMTYIRNLCIQGRQGKDRISHQ-
RQQQKRSDRIARFLQLQCHSLILMDMTKWIKR--------
>Potri.009G124400_Populus_trichocarpa
MRTICDVCESAAAILFCAADEAALCRSCDEKVHLCNKLASRHVRVGLADPSAVPQCDICENAPAFFYC
EIDGSSLCLQCDMIVHVGGKRTHGRYLLLRQRVEFPGDKPGRMEEQGQQ--
PLDHNETRRDQNQPLKLTARENKQNHRASPVPMVENNTDSDGKMDNNLIDLNARPQRIHGQNST
NQE---------NHESSSAVPVGSFKREPQK-------
>Pp3c7_5280V3_Physcomitrella_patens
MRTLCDVCEAAPARLFCAADEAALCLKCDEKVHSCNKLANRHVRLELAESRAVPRCDICENAPAFFF
CGVDGTSLCLQCDMDVHVGGKKAHERYLMMGQRVELPSRKLRFEDNVDTEKLPAEPSNAPTDKNG
-
VLPDHHHHHHYHHHHHHHHHEDEVRAGLPAPKQSCDRDASNAPAIAITAGDEESLLIDGCIHQNQ
PRMIDLNSRPKRLQSQASVPDKG
>Pp3c11_20560V3_Physcomitrella_patens
MRTLCDVCEAAPARLFCAADEAALCLKCDEKVHSCNKLAYRHVRLELAESRPVPRCDICENAPAFFFC
GVDGTSLCLQCDMDVHVGGKKAHERYLMMRQRVELPSRKLRFEDTVDTEKPTAEPNSVPADKNGT
LLPDQHHHHHYHHHHHHHHHEDELRAGLPASKPSCDRDNSNAPAIAIAAGDEESLLLDDCLVQNQ
SRMIDLNSRPKRLQNQASVPDKG

((((BBX18_Arabidopsis_thaliana:0.24850072,BBX19_Arabidopsis_thaliana:0.24503341)0.9600:
0.06049503,(Potri.005G117100_Populus_trichocarpa:0.25727320,Potri.007G015200_Populus_
trichocarpa:0.28505109)0.7180:0.03160511)0.4670:0.01273844,((Glyma.11G074900_Glycine_
max:0.01639498,Glyma.01G168500_Glycine_max:0.01310569)1.0000:0.15321470,((Glyma.11
G110900_Glycine_max:0.01925182,Glyma.12G037200_Glycine_max:0.01625487)1.0000:0.115
01614,(Potri.004G162600_Populus_trichocarpa:0.03540801,Potri.009G124400_Populus_trich
ocarpa:0.03068210)1.0000:0.07641888)0.9990:0.05867552)0.9810:0.07827847)0.4830:0.0229
8339,(LOC_Os09g35880_Oryza_sativa:0.15050293,(GRMZM2G143718_Zea_mays:0.15005853
,GRMZM2G422644_Zea_mays:0.15324886)0.8790:0.04161924)0.9990:0.09948703,(Pp3c7_52
80V3_Physcomitrella_patens:0.11921626,Pp3c11_20560V3_Physcomitrella_patens:0.1230034
4)1.0000:0.32572532);
