# Supplementary material for: BBX19 fine-tunes the circadian rhythm by interacting with PSEUDO-RESPONSE REGULATOR proteins to facilitate their repressive effect on morning-phased clock genes
Source: Plant Cell. 2021 May 14;33(8):2602–17. doi: 10.1093/plcell/koab133 (PMC8408442; doi:10.1093/plcell/koab133)
Supplement: koab133_Supplementary_Data [file koab133_supplementary_data.zip › tpc.00221.2021-s05.pdf]

BBX19 fine-tunes the circadian rhythm by interacting with PSEUDO-RESPONSE REGULATOR proteins to facilitate their repressive effect on morning-phased clock genes

Li Yuan, Yingjun Yu, Mingming Liu, Yang Song, Hongmin Li, Junqiu Sun, Qiao Wang, Qiguang Xie, Lei Wang and Xiaodong Xu

Corresponding author: Xiaodong Xu xiaodong.xu@henu.edu.cn

Review timeline:

**TPC2020-RA-01000**	Submission received:	December 1, 2020
	1st Decision:	January 6, 2020 *manuscript declined*
**TPC2021-RA-00187D**	Submission received:	March 1, 2021
	1st Decision:	March 8, 2021 *manuscript declined*
**TPC2021-RA-00221D**	Submission received:	March 9, 2021
	1st Decision:	April 5, 2021 *revision requested*
**TPC2021-RA-00221DR1**	1st Revision received:	April 13, 2021
	2nd Decision:	April 18, 2021 *acceptance pending, sent to science editor*
	Final acceptance:	May 11, 2021

REPORT: (The report shows the major requests for revision and author responses. Minor comments for revision and miscellaneous correspondence are not included. The original format may not be reflected in this compilation, but the reviewer comments and author responses are not edited, except to correct minor typographical or spelling errors that could be a source of ambiguity.)

TPC2020-RA-01000 1st Editorial decision – *declined* January 6, 2020

Your submission has been evaluated by members of the editorial board as well as expert reviewers in your field, and we regret to inform you that we are not able to recommend publication of this manuscript in its current form. We have not made this decision lightly. We have had input from multiple scientists, and we have solicited post-review comments as well. Our present policy is to offer streamlined decisions and to not advise on the direction of the work by requesting extensive modifications or substantial additional experiments.

The reviewers found the topic of the manuscript interesting but were critical of the data based on the lack of negative controls in some key experiments. The reviewers also had concerns about your conclusions on protein function being drawn without testing protein accumulation. In the editorial assessment, the editors were also concerned that the genetic data were based on analysis of one bbx19 mutant allele with no complementation; the conclusions on the role of BBX19 in regulating period length would need to be confirmed with a second allele or complementation. In addition, the quantification of the ChIP data did not look very convincing and was difficult to compare as the data were shown in different panels. Based on these extensive criticisms, we must decline this version of your manuscript. We would be prepared to consider a revised version, but only if it fully answers the comments of the reviewers and editors by including the required new data.

During the post-review consultation session, we agreed that if you could address the major points raised by the reviewers by new experiments, we would welcome a resubmission. This may be treated as a new submission, but we would attempt to use at least some of the same reviewers. Nevertheless, reviewers will be asked to assess as a new manuscript (i.e. are the claims fully supported by the data and do the results presented move the field forward?), and not only whether previous reviewer comments have been addressed.

In addition, we find that the writing suffers from some problems with grammar and awkward phrasing, and in general lacks clarity and conciseness. We strongly encourage the use of a professional editing service to ensure that the work is presented in the best manner and reaches the broadest possible audience. Several editing services have agreed to extend a discount to authors coming to their website via this ASPB page https://aspb.org/aspb-journal-submission-editing-services/.

It will be important to convince the editors and reviewers that the study adds significant new understanding(s) of mechanisms or processes, or otherwise represents a significant advance in the field, and that the major claims made are fully justified by the data presented. This includes careful consideration and explanation of the various controls used in experiments, the extent and manner of replication, and the statistical analyses used. Sampling methods and the nature of "biological replicates" should be described precisely (i.e. different plants, parts of plants, pooled tissue, independent pools of tissue, sampled at different times, etc.), along with a clear description of and rationale for any statistical analyses conducted. The reader should know exactly what was sampled; what forms the basis of the calculation of any means and other statistical variables and parameters reported. This is also necessary to ensure that proper statistical analysis was conducted.

--- Reviewer comments:

Reviewer #1 (Comments for the Author):

Yuan et al. demonstrated that BBX19 is involved in the regulation of the circadian clock. They found that the expression patterns of BBX19 and related BBXs resembled with the morning clock gene ones. The bbx19 single mutant, not other related bbx mutants, showed slight short period phenotypes, indicating that BBX19 may influence the circadian clock. The authors showed that BBX19 (and BBX18) physically interact with PRR5, PRR7, and PRR9 proteins in heterologous systems as well as in Arabidopsis. These interactions occurred in the different time of the day. They also demonstrated that overexpression of BBX19 altered the expression of genes related to the circadian clock, including core clock genes. BBX19 can associate with the CCA1, LHY, and REV8 promoters and overexpression of BBX19 may attenuate the expression levels of these genes. Although the contribution of BBX19 seems to be minor, their results indicate the involvement of BBX19 in the circadian clock gene circuit. Below are several major concerns and minor comments, all of which needed to be addressed.

Major concerns:

1. The difference in results shown in Figures 7D and 8A is intriguing. In Figure 7D, the effect of BBX19 overexpression on CCA1 transcript levels is very weak, while in Figure 8A, the same BBX19 overexpression eventually depressed the circadian oscillation of CCA1:LUC activities completely. Is this caused by the difference of what they measured (the CCA1 promoter activity vs the CCA1 transcript levels)?or even for Figure 7D, if the authors analyzed the expression of CCA1 transcripts under the constant light conditions for longer time courses (maybe at least for more than 2 days), will they see the clear difference at the later time points, similar to the Figure 8A result? Since the clear CCA1:LUC activity changes caused by the overexpression of BBX19 were visible at the time points later than 24 hours after the induction of BBX19 expression, the authors should analyze the expression levels of CCA1 under the same longer time course conditions for Figure 7D to 7F (i.e. they should analyze the expression of CCA1 and other clock genes for longer time courses).

2. Also, for Figures 8A to 8C, the authors need to analyze BBX19 transcript levels, so that they could show that BBX19 is induced at the same levels under these genetic backgrounds (WT, prr7 prr9 and prr5 prr7). Otherwise, it is difficult to assess the effects of the prr mutations on the BBX19 function. Are BBX19 protein levels altered in the prr7 prr9 and prr5 prr7 backgrounds? As their BBX19 construct contains the YFP-HA tag, the authors can easily analyze whether the BBX19 protein levels are affected by prr mutations or not. This information (=BBX19 protein levels) is also crucial to understand the ChIP results shown in Figures 8E and 8F. By the way, the ChIP result difference between WT and prr9 prr7 backgrounds are very small. Perhaps, the authors should try BBX19 ChIP in the prr5 prr7 mutant as well. Since the difference is minor, having more supporting evidence will make their statement more convincing.

3. For Figure 4D, the authors analyzed the effects of either PRR9 EAR motif deletion or the PR domain deletion on the interaction with BBX19 using the luciferase complementation analysis. It is interesting to see that the BBX19 and the EAR motif deleted PRR9 pair showed higher LUC activity than the BBX19-intact PRR9 pair. Since the authors did not show whether these deletions changes the protein stability of these PRR9 variants, they cannot rule out the possibility that these deletions changed the protein stability but not the strength of the interaction. To eliminate this possibility, they need to show that these deletions did not change the stability of these PRR9 variants (or show these PRR9 variants are expressed at the similar levels with PRR9).

Minor issues:

1. On line 117, please remove the word "vital". As BBX19 has a minor role in the circadian clock, this sentence is overstated.

2. On lines 116 and 119, it would be informative if the authors add the average period length results for the BBX18 and BBX19 minigene overexpressors and the bbx18 bbx19 double mutants (together with the single mutants) in the text, since the differences in period length are small.

3. On line 141, the "BBX19 homodimer" should be the "BBX18 homodimer".

4. On lines 164-165, the period length phenotype results of the double mutants between bbx19 and prrs could not support the following statement "The result indicated that PRR9, 7 and BBX19 work together to regulate the pace of the clock". It is better to remove it.

5. For Figures S5 C and D, it looks like the same results were plotted for two days. The authors said they analyzed only for 1 day. They need to clearly state that they plotted the same results twice in the figure legend. They also need to change the X-axis labelling. The number should not go over 24 hours as they did not analyze the results for more than 24 hours (the current figure said "-6" to "48 h", which is totally misleading).

6. On line 238, please remove the word "evidently" as the difference mentioned is very minor. Related to this comment, on line 32, the author should remove or rephrase this summary sentence. Their results did not support this statement at all. If they would like to say that PRR proteins (I assume they mean PRR5, 7 and 9) are "required" for BBX19 to bind to the G-box containing regions of CCA1, LHY, and RVE8 promoters, they should test BBX19 binding at least in the prr 5 ppr7 prr9 triple mutant background.

7. There is no description of number of biological replicates used for their RNA-seq analysis. The authors should provide the basic information about the RNA seq (i.e. how many reads were obtained per sample, and how many reads were mapped to the reference genome, etc.) as well.

Reviewer #2 (Comments for the Author):

This paper describes identification characterization of the gene BBX19, which encodes a member of the B-box zinc-finger subfamily BBX IV in Arabidopsis that is involved in controlling the function of the circadian clock. In this paper they found that overexpression of BBX19 by using the estradiol-responsible ER8 promoter strongly dampens rhythmic expression of CCA1 to a low level in LL, suggesting its significant role in suppressing CCA1 transcription and controlling the circadian-clock function. They also found that loss of the BBX19 function shortens the period length of circadian expression of CCA1, whereas moderately increasing the level of BBX19 transcription through its own promoter slightly lengthens its rhythmic expression. The authors also showed that BBX19 physically binds to PRR9, PRR7 and PRR5 in planta, which act to suppress transcription of CCA1, mainly at times when expression of BBX19 coincides with that of PRRs. BBX19 was also found to bind to the CCA1 promoter, consistent with the idea that this factor directly suppresses expression of CCA1 by physically binding to and cooperating with PRRs. This idea has been further supported by the observation of a genetic experiment that the bbx19 mutation does not further shorten the circadian period in prr5/prr7 and data that prr mutations reduce the binding efficiency of BBX19 to the CCA1 promoter.

This paper demonstrates a novel molecular mechanism by which transcription of a major clock gene CCA1 is controlled in the circadian clock in Arabidopsis thaliana. This finding would be significant in the area of the circadian clock providing general interest, and therefore suitable for publication in the Plant Cell. However, I feel that the quality of data is often not enough because required control experiments have not been carried out. Prior to publication several points including missing control experiments would need to be further addressed.

Major points

1.Data with negative controls are missing in BiFC in Fig.3. Each of proteins fused to YC alone has to be expressed in N. benthamiana to check whether the YFP signal cannot be observed. This experiment also has to be performed for each of proteins fused to YN.

2. As for experiments for checking temporal interaction between BBX18/19 and PRRs in planta in Fig. 4, data with control plants are missing. Please provide luc data in BBX18-nLuc, BBX19-cLuc and BBX19-nLuc alone. Also, as for the data showing the domain necessary for PRR9 to bind to BBX19, please provide data of PRR9 accumulation in these transgenic lines so that binding efficiency of these PRR9 proteins to BBX19 can be comparable with each other. Alternatively, please perform co-IP in N. benthamiana, by which accumulation levels of these PRR9 versions can easily be accessed.

3. As for experiments for checking requirement of PRRs for association of BBX19 to the CCA1 promoter, several control data are missing. In the experiment for demonstrating effects of prr mutations on BBX19-ox mediated changes in CCA1 expression rhythm, data of BBX19 mRNA levels in pER8-BBX19/Col, pER8-BBX19/prr7prr9, and pER8-BBX19/prr5prr7 are missing. Please provide this data so that luc activities among these lines can really be comparable with each other. Also, in ChIP assays it is unclear whether the effect of prr7prr9 mutations on binding of BBX19 to the CCA1 promoter is statistically significant. Please provide with statistics data in which all the three biological replicates have been combined. Overall, present data are not enough to conclude that PRRs recruit BBX19 to the CCA1 promoter.

Minor points

1. On line 147 in the text, please provide explanation about the EAR domain.

2. On line 183 in the text, please explain pER8 in more detail.

3. On line 241 in the text, the authors describe "BBX19 negatively regulates morning-phased clock gene expression by recruiting PRR9, 7 proteins to their promoters". But what authors suggested in this paper is that PRR9/7 recruit BBX19 to promoters of morning-phased genes, so please change this description, or delete this sentence depending on results of additional experiments suggested in this comment. The similar description is also found on line 258 in Discussion section.

4. In figure 7A, please provide more detailed information about DBD, DBS and RNL.

Reviewer #3 (Comments for the Author):

Yuan and collaborators report findings on the involvement of BBX18 and BBX19 in the circadian clock. Although only BBX19 mutation has an impact on circadian period, shortening it, overexpression of both BBX19 and BBX18 lengthens the circadian period. Interestingly, both BBX18 and BBX19 interact with themselves, with each other and with PRRs (9, 7 and 5). This interaction with PRRs seems to occur in a phased manner, coinciding with the peak expression phases of the PRRs. Next, the authors focus on BBX19 and show that it seems to impinge on a common set of target genes as the PRRs, particularly repressing morning phased clock genes. Finally, the authors propose that the PRRs are required for the recruitment of BBX19 to the CCA1 promoter, although I do not agree with this last statement as I find it not well supported by the data (and also not well depicted in the model in Figure 9).

Little is known about regulators of the transcriptional activity of clock proteins, and hence the reported findings are interesting to the field. I find however that some statements (e. g. regarding the requirement of PRRs) should be backed up by more data or toned down. Another major concern is the language, as in many instances the text is difficult to understand and leads to confusion. The manuscript would undoubtedly benefit from English proofreading.

After carefully reading the manuscript, I encourage the authors to consider the following comments.

- Line 112, Figure 1 D, the mean period length of CCA1:LUC oscillations in bbx19-3 mutants was determined to be 23.6 h but the figure shows a mean period of 23.5 h. Please revise it. Also, regarding statistical analyses, in the figure legend it is stated that "at least 24 individual seedlings were used" but supplemental Table S2 shows a maximum of 24. Additionally, an ANOVA should have been performed instead of a t-test.

- Line 114: "evolutionary analyses in multiple species suggested the roles for B-box domains at their N-terminus in DNA binding and transcriptional regulation". I wonder, how do authors deduce this from supplemental figure 2? The analyses rather show that the N-terminus is conserved.

- Line 117: "...indicating that they play a vital role in the maintenance of the circadian clock". Authors should tone down the message here. The BBX are required to keep the pace of the clock, but are not vital, as the clock still runs in the bbx mutants.

- Line 123, I would rather just say that BBX19 and 18 are involved in adjusting the pace of the clock and are rhythmically expressed in the day. BBX18:BBX18 and BBX19:BBX19 lines lengthen the pace of the clock. One would assume that both proteins are still rhythmically expressed (because their expression is driven by an endogenous promoter fragment), though they likely reach higher levels than the WT, therefore slowing down the clock. So, it is not only the rhythmic expression of these BBX proteins what is important for period length, but also their level.

-Figure 2 legend would also benefit from some revision, better stating what information refers to which panel or panels.

- Figure 3A, I see weak interaction with ELF3 and maybe also for TOC1 in yeast. Did the authors investigate these possible associations any further (through BiFC or any other assay)

- Figure 3B, please provide negative controls for BiFC assays in which each fusion protein is co-expressed with the empty vector, or ideally with the other half of YFP fused to an unrelated protein.

- Line 137, I guess authors mean here that the expression of the fusion proteins is driven by their own promoters.

- Figure 4, it would be nice to see a negative control, given that, depending on the combination (nLUC or cLUC fused to the N or C terminus), the split luciferase assay is prone to giving non-specific signal. Nonetheless, I would assume that the BBX19-cLUC/PRR9-delPR-nLUC, where no LUC activity (as consequence of apparent lack of interaction) is detected, serves as negative control for this assay (please, include also the combination with BBX18-cLUC). In this regard, for panel D it would be nice to add a rescaled version (maybe added to supplementary figures) where the dynamics of LUC activity, if any, from the BBX19-cLUC/PRR9-delPR-nLUC F1 seedlings can be observed. Also, to serve as true negative control, the lack of interaction between BBX19 (and BBX18) with PRR9-delPR should be tested by other means. Why do panels A, B and C show "normalized LUC activity" and D "LUC activity"? How was it normalized? This happens in other figures, too, where some panels display normalized values and others do not.

- Line 141, Figure 4A, isn´t the pattern of interaction more like the BBX18 homodimer?

- Line 144, "Collectively, our findings suggested that BBX19 and BBX18 likely act as partners of sequentially expressed PRR9, 7, 5 to temporally regulate the expression of their target genes." I am not sure whether anything on the effect of BBX18/29 on PRR target gene expression can be stated at this point of the manuscript.

- Line 150, nothing is shown with regard to BBX protein recruitment to the chromatin, only important domains for protein-protein interactions. Also, as stated earlier, for Figure 4D it would be nice to add a rescaled version where the dynamics of the BBX19-cLUC/PRR9-delPR-nLUC interaction, or the lack of, can be observed.

- Line 164, Figure 5, when analyzing genetic interactions, an additive effect is considered as suggesting parallel roles of the investigated genes. That would be the case of BBX19 and PPR5, as well as BBX19 and CCA1, BBX19 and LHY, and BBX19 and TOC1. But the authors interpret these additive effects differently in each case. Could the authors please elaborate on this? Also, looking at the intermediate effects observed for BBX19 and PRR9/PRR7, which might be due to redundancy, it would be interesting to analyze period length in bbx19 prr9 prr7 triple mutants. When combined with clock mutants, bbx19 always shortens the period, but in the case of prr5 prr7 bbx19, which suggests that BBX19 imposes a brake to the pace of the clock. The physical interaction with PRR5, 7, and 9 and the epistasis of prr5,7 over bbx19 suggest that this action takes place through the interaction with these PRRs.

-Line 168, I would rather say that the prr7 prr5 mutation is epistatic over bbx19.

- Line 223, overexpression of BBX19 strongly reduces CCA1:LUC activity. Looking back at Figure 2A, the repressive effect of BBX19 can also be seen (and in Figure 2D, too), but the opposite seems to happen for BBX18, which seems to slightly induce CCA1:LUC (see also the intermediate effect of the double bbx18 bbx19 mutant on CCA1:LUC amplitude). Could the authors please elaborate on the possible different functions of BBX18 and BBX19, even though they both interact with PRRs at the same times (Figure 4)? Figure S4B is also a bit puzzling for me, because such a reduction in TOC1:LUC would be consistent with an increase in CCA1, not a reduction. Finally, regarding the

requirement of the PRRs, CCA1:LUC reduction by BBX19 in the prr7/9 and prr5/7 mutants might be less pronounced, but still very significant.

-Figure 8E-F. The interpretation of results in these two panels would benefit from a reorganization of the information provided. I'd suggest the authors to show enrichments in the WT and in the mutant side-by-side for better comparison. ChIP analysis for each gene can be presented as separate panels.

- Line 237, the "G-box-containing elements" are not necessary, or at least the authors do not show if they are or not. Only thing that can be stated is that BBX19 binds to a region around the G-box. Furthermore, the association to some promoters but not all (there is practically no difference in the association to the CCA1 promoter) is reduced in the absence of PRR7 and PRR9, which again makes the claim about the requirement of PRRs doubtful. Are PRRs required for the association, for the repressive effect of BBX19 or for both?

- Line 241, also line 258, to show that BBX19 recruits PRRs, a ChIP of PRRs in the bbx19 mutant should have been performed. Otherwise, the claim is not supported by the data.

TPC2021-RA-00187D Submission received	March 1, 2021

Reviewer comments on previously declined manuscript and **author responses:**

Thank you for the comments to improve our work. In the resubmitted manuscript, we made detailed revisions to the full text. A total of 8 Figures with new panels (see Figure 4, 5, 7, 8; Figure S4, S5, S6, S7), 1 new table (Table S3) were supplemented. In order to increase the readability of the data, we have replotted Figure 1D, Figure 2, and Figure 6.

In addition, here is a reply to the reviewers' comments point to point:

Reviewer #1 (Comments for the Author):

Yuan et al. demonstrated that BBX19 is involved in the regulation of the circadian clock. They found that the expression patterns of BBX19 and related BBXs resembled with the morning clock gene ones. The bbx19 single mutant, not other related bbx mutants, showed slight short period phenotypes, indicating that BBX19 may influence the circadian clock. The authors showed that BBX19 (and BBX18) physically interact with PRR5, PRR7, and PRR9 proteins in heterologous systems as well as in Arabidopsis. These interactions occurred in the different time of the day. They also demonstrated that overexpression of BBX19 altered the expression of genes related to the circadian clock, including core clock genes. BBX19 can associate with the CCA1, LHY, and REV8 promoters and overexpression of BBX19 may attenuate the expression levels of these genes. Although the contribution of BBX19 seems to be minor, their results indicate the involvement of BBX19 in the circadian clock gene circuit. Below are several major concerns and minor comments, all of which needed to be addressed.

Major concerns:

1. The difference in results shown in Figures 7D and 8A is intriguing. In Figure 7D, the effect of BBX19 overexpression on CCA1 transcript levels is very weak, while in Figure 8A, the same BBX19 overexpression eventually depressed the circadian oscillation of CCA1:LUC activities completely. Is this caused by the difference of what they measured (the CCA1 promoter activity vs the CCA1 transcript levels)?or even for Figure 7D, if the authors analyzed the expression of CCA1 transcripts under the constant light conditions for longer time courses (maybe at least for more than 2 days), will they see the clear difference at the later time points, similar to the Figure 8A result? Since the clear CCA1:LUC activity changes caused by the overexpression of BBX19 were visible at the time points later than 24 hours after the induction of BBX19 expression, the authors should analyze the expression levels of CCA1 under the same longer time course conditions for Figure 7D to 7F (i.e. they should analyze the expression of CCA1 and other clock genes for longer time courses).

Authors' Response

Thank you for the suggestions.

In the resubmitted manuscript, we analyzed the expression of *CCA1*, *LHY*, and *RVE8* for 2 days in LL under *BBX19* overexpression conditions (Figure 7). We observed that the accumulation of *CCA1*, *LHY*, and *RVE8* transcripts began to decline in Col-0 within 12 h after the treatment with estradiol. After 12 h, the level of transcripts of each gene was extremely low. This result is similar to the phenotype of *CCA1:LUC* promoter activity inhibited by BBX19 (Figure 8A).

In addition, we further analyzed the function of *BBX19* overexpression on morning-phased genes in the *prr7-3 prr9-1* and *prr5-1 7-3* mutants. The results showed that the inhibitory effect of BBX19 on *CCA1*, *LHY*, or *RVE8* expression in the mutant was significantly weaker than that of the wild type (Col-0) (Figure 7). The data predicts that PRR9, 7, 5 are required for BBX19 to negatively regulate the expression of *CCA1*, *LHY*, or *RVE8*.

2. Also, for Figures 8A to 8C, the authors need to analyze BBX19 transcript levels, so that they could show that BBX19 is induced at the same levels under these genetic backgrounds (WT, prr7 prr9 and prr5 prr7). Otherwise, it is difficult to assess the effects of the prr mutations on the BBX19 function. Are BBX19 protein levels altered in the prr7 prr9 and prr5 prr7 backgrounds? As their BBX19 construct contains the YFP-HA tag, the authors can easily analyze whether the BBX19 protein levels are affected by prr mutations or not. This information (=BBX19 protein levels) is also crucial to understand the ChIP results shown in Figures 8E and 8F. By the way, the ChIP result difference between WT and prr9 prr7 backgrounds are very small. Perhaps, the authors should try BBX19 ChIP in the prr5 prr7 mutant as well. Since the difference is minor, having more supporting evidence will make their statement more convincing.

Authors' Response

Thank you for the suggestions.

We performed a 48-hour qRT-PCR analysis of the Col-0, *prr7-3 prr9-1*, and *prr5-1 7-3* materials (Figure 7A). After treating estradiol at ZT12 under free-running conditions (LL), the *BBX19* transcript level in the *pER8-BBX19*/*prr7-3 prr9-1* or *pER8-BBX19*/*prr5-1 prr7-3* mutant is similar to its expression in the *pER8-BBX19*/Col-0.

In addition, western bolt detection was performed in the materials used for ChIP analysis (supplemental Figure S7). After 15 h of the estradiol treatment, there was no difference in the accumulation of BBX19-YFP-HA protein in the wild type, *prr7-3 prr9-1*, and *prr5-1 7-3* mutants (supplemental Figure S7). The results indicate that the background of *prr7-3 prr9-1* and *prr5-1 7-3* mutations does not affect the accumulation of BBX19 protein level.

We have completed the ChIP experiments in the three materials: Col-0, *prr7-3 prr9-1*, and *prr5-1 7-3* mutants (Figure 8E). The statistical analysis of three biological replicates showed that in the *prr7-3 prr9-1* and *prr5-1 prr7-3* mutants, the binding of BBX19 to the *CCA1*, *LHY* and *RVE8* promoter regions did decreased to a certain extent.

3. For Figure 4D, the authors analyzed the effects of either PRR9 EAR motif deletion or the PR domain deletion on the interaction with BBX19 using the luciferase complementation analysis. It is interesting to see that the BBX19 and the EAR motif deleted PRR9 pair showed higher LUC activity than the BBX19-intact PRR9 pair. Since the authors did not show whether these deletions changes the protein stability of these PRR9 variants, they cannot rule out the possibility that these deletions changed the protein stability but not the strength of the interaction. To eliminate this possibility, they need to show that these deletions did not change the stability of these PRR9 variants (or show these PRR9 variants are expressed at the similar levels with PRR9).

Authors' Response

Thank you for the suggestions.

We detected the accumulation of PRR9 protein in the materials with EAR or PR domain deletions (Figure 4E). The materials were sampled at the ZT5, which is the peak time of the interaction between BBX19 and PRR9 under 12L/12D conditions. The results showed that the levels of PRR9 protein in wild-type *PRR9-*

***nLUC*, *PRR9-delPR-nLUC*, and *PRR9-delEAR-nLUC* were similar. Therefore, the interaction between proteins affected by the deletion of PR domain of PRR9 is not related to the stability of PRR9 protein variants.**

In addition, we completed yeast two-hybrid analysis, confirming that the interaction between BBX19 and PRR9 depends on its PR domain (Figure 4F).

Minor issues:

1. On line 117, please remove the word "vital". As BBX19 has a minor role in the circadian clock, this sentence is overstated.

Authors' Response

Thank you for your revised comments.

We deleted the overstated word and changed it to point out more pertinently that BBX19 may play a certain role in regulating the circadian clock.

2. On lines 116 and 119, it would be informative if the authors add the average period length results for the BBX18 and BBX19 minigene overexpressors and the bbx18 bbx19 double mutants (together with the single mutants) in the text, since the differences in period length are small.

Authors' Response

Thank you for your revised comments.

We have added the value of the circadian periodicity to the text for easy reading and comparison.

3. On line 141, the "BBX19 homodimer" should be the "BBX18 homodimer".

Authors' Response

Thank you for reminding this typo. We have corrected this.

4. On lines 164-165, the period length phenotype results of the double mutants between bbx19 and prrs could not support the following statement "The result indicated that PRR9, 7 and BBX19 work together to regulate the pace of the clock". It is better to remove it.

Authors' Response

Thank you for the analysis. This statement in last manuscript was indeed inaccurate, and we have removed this sentence as suggested.

5. For Figures S5 C and D, it looks like the same results were plotted for two days. The authors said they analyzed only for 1 day. They need to clearly state that they plotted the same results twice in the figure legend. They also need to change the X-axis labelling. The number should not go over 24 hours as they did not analyze the results for more than 24 hours (the current figure said "-6" to "48 h", which is totally misleading).

Authors' Response

Thank you for pointing out the problem with the X-axis labeling. In new supplemental Figure S9C-D (Figure S5C-D in the last manuscript), PRC waveforms were double-plotted to emphasize the pacemaker sensitivity to light pulses. However, in the time course of light pulses processing, it should still be the actual processing time, that is, one cycle of CT0-24. In the result section of the resubmitted manuscript, that is, Figure S9C-D, we have made a revision for this.

6. On line 238, please remove the word "evidently" as the difference mentioned is very minor. Related to this comment, on line 32, the author should remove or rephrase this summary sentence. Their results did not support this statement at all. If they would like to say that PRR proteins (I assume they mean PRR5, 7 and 9) are "required" for BBX19 to bind to the G-box containing regions of CCA1, LHY, and RVE8 promoters, they should test BBX19 binding at least in the prr 5 ppr7 prr9 triple mutant background.

Authors' Response

Thank you for the revised comments. We have weakened the concluding sentence, and removed or replaced inappropriate words.

7. There is no description of number of biological replicates used for their RNA-seq analysis. The authors should provide the basic information about the RNA seq (i.e. how many reads were obtained per sample, and how many reads were mapped to the reference genome, etc.) as well.

Authors' Response

Thank you for the revised comments.

We used RNA-seq data from three biological replicates with the reads ranging from 10.9 to 11.2 million per sample. The information about the RNA seq has been updated to the Material and Method section.

Reviewer #2 (Comments for the Author):
This paper describes identification characterization of the gene BBX19, which encodes a member of the B-box zinc-finger subfamily BBX IV in Arabidopsis that is involved in controlling the function of the circadian clock. In this paper they found that overexpression of BBX19 by using the estradiol-responsible ER8 promoter strongly dampens rhythmic expression of CCA1 to a low level in LL, suggesting its significant role in suppressing CCA1 transcription and controlling the circadian-clock function. They also found that loss of the BBX19 function shortens the period length of circadian expression of CCA1, whereas moderately increasing the level of BBX19 transcription through its own promoter slightly lengthens its rhythmic expression. The authors also showed that BBX19 physically binds to PRR9, PRR7 and PRR5 in planta, which act to suppress transcription of CCA1, mainly at times when expression of BBX19 coincides with that of PRRs. BBX19 was also found to bind to the CCA1 promoter, consistent with the idea that this factor directly suppresses expression of CCA1 by physically binding to and cooperating with PRRs. This idea has been further supported by the observation of a genetic experiment that the bbx19 mutation does not further shorten the circadian period in prr5/prr7 and data that prr mutations reduce the binding efficiency of BBX19 to the CCA1 promoter.

This paper demonstrates a novel molecular mechanism by which transcription of a major clock gene CCA1 is controlled in the circadian clock in Arabidopsis thaliana. This finding would be significant in the area of the circadian clock providing general interest, and therefore suitable for publication in the Plant Cell. However, I feel that the quality of data is often not enough because required control experiments have not been carried out. Prior to publication several points including missing control experiments would need to be further addressed.

Major points

1.Data with negative controls are missing in BiFC in Fig.3. Each of proteins fused to YC alone has to be expressed in N. benthamiana to check whether the YFP signal cannot be observed. This experiment also has to be performed for each of proteins fused to YN.

Authors' Response

Thank you for the revised comments.

We uploaded the negative controls of BiFC assays, which was attached to the resubmitted manuscript as supplemental Figure S4.

2. As for experiments for checking temporal interaction between BBX18/19 and PRRs in planta in Fig. 4, data with control plants are missing. Please provide luc data in BBX18-nLuc, BBX19-cLuc and BBX19-nLuc alone. Also, as for the data showing the domain necessary for PRR9 to bind to BBX19, please provide data of PRR9 accumulation in these transgenic lines so that binding efficiency of these PRR9 proteins to BBX19 can be comparable with each other. Alternatively, please perform co-IP in N. benthamiana, by which accumulation levels of these PRR9 versions can easily be accessed.

Authors' Response

Thank you for the comments.

We uploaded the data of the controls to the supplemental Figure S5 in the resubmitted manuscript. In addition, the raw bioluminescence counts of LUC were also provided to describe the temporal relations of the BBX19-PRRs complex in the LCA assay (Figure 4B-C).

In addition, we detected the accumulation of PRR9 protein in the materials with EAR or PR domain deletions (Figure 4E in the resubmitted manuscript). The materials were sampled at the ZT5, which is the peak time of the interaction between BBX19 and PRR9 under 12L/12D conditions. The results showed that the levels of PRR9 protein in wild-type *PRR9-nLUC*, *PRR9-delPR-nLUC*, and *PRR9-delEAR-nLUC* were similar. Therefore, the interaction between proteins affected by the deletion of PR domain of PRR9 is not related to the stability of PRR9 protein variants. Also, we completed yeast two-hybrid analysis, confirming that the interaction between BBX19 and PRR9 depends on its PR domain.

3. As for experiments for checking requirement of PRRs for association of BBX19 to the CCA1 promoter, several control data are missing. In the experiment for demonstrating effects of prr mutations on BBX19-ox mediated changes in CCA1 expression rhythm, data of BBX19 mRNA levels in pER8-BBX19/Col, pER8-BBX19/prr7prr9, and pER8-BBX19/prr5prr7 are missing. Please provide this data so that luc activities among these lines can really be comparable with each other. Also, in ChIP assays it is unclear whether the effect of prr7prr9 mutations on binding of BBX19 to the CCA1 promoter is statistically significant. Please provide with statistics data in which all the three biological replicates have been combined. Overall, present data are not enough to conclude that PRRs recruit BBX19 to the CCA1 promoter.

Authors' Response

Thank you for the revised comments.

We performed a 48-hour qRT-PCR analysis of *BBX19* mRNA levels in the Col-0, *prr7-3 prr9-1*, and *prr5-1 7-3* materials (Figure 7A in the resubmitted manuscript). After treating estradiol at ZT12 under free-running conditions (LL), the BBX19 transcript level in the *pER8-BBX19/prr7-3 prr9-1* or *pER8-BBX19/prr5-1 prr7-3* mutant is similar to its expression in the *pER8-BBX19*/Col-0.

In addition, western bolt was performed in the materials used for ChIP analysis (see supplemental Figure S7 in the resubmitted manuscript). After 15 h of estradiol treatment, there was no difference in the accumulation of BBX19-YFP-HA protein in the wild type, *prr7-3 prr9-1*, and *prr5-1 7-3* mutants. The results indicated that the background of *prr7-3 prr9-1* and *prr5-1 7-3* mutations does not affect the accumulation of BBX19 protein level.

Also, we completed the ChIP assay in the wild type, *prr7-3 prr9-1*, and *prr5-1 7-3* mutants (Figure 8E in the resubmitted manuscript). Three biological replicates were analyzed for each material. The results showed that in the *prr7-3 prr9-1* and *prr5-1 prr7-3* mutants, BBX19 had a reduction in the binding of *CCA1*, *LHY* and *RVE8* promoter regions.

We further analyzed the function of *BBX19* overexpression on the morning-phased genes (*CCA1*, *LHY* and *RVE8*) in the *prr7-3 prr9-1* and *prr5-1 7-3* mutants. The results showed that the inhibitory effect of *BBX19* on *CCA1*, *LHY*, or *RVE8* expression in the mutants were significantly weakened than that of the wild type (Col-0) (new Figure 7 in the resubmitted manuscript). The data predicted that PRR9, 7, 5 are required for BBX19 to negatively regulate the expression of *CCA1*, *LHY*, or *RVE8*.

Minor points

1. On line 147 in the text, please provide explanation about the EAR domain.

Authors' Response

Thank you for the revised comments. We described the role of EAR motif in plant transcriptional regulator proteins and cited relevant reference.

2. On line 183 in the text, please explain pER8 in more detail.

Authors' Response

Thank you for the revised comments. We described the use of *pER8* vector system to check the estradiol inducible expression of foreign gene, and attached references.

3. On line 241 in the text, the authors describe "BBX19 negatively regulates morning-phased clock gene expression by recruiting PRR9, 7 proteins to their promoters". But what authors suggested in this paper is that PRR9/7 recruit BBX19 to promoters of morning-phased genes, so please change this description, or delete this sentence depending on results of additional experiments suggested in this comment. The similar description is also found on line 258 in Discussion section.

Authors' Response

You made the pertinent comments on the data. We accept your suggestion and have modified the discussion.

4. In figure 7A, please provide more detailed information about DBD, DBS and RNL.

Authors' Response

Thank you for the revised comments. We have made notes in the legend (see new Figure 6E in the resubmitted manuscript).

Reviewer #3 (Comments for the Author):

Yuan and collaborators report findings on the involvement of BBX18 and BBX19 in the circadian clock. Although only BBX19 mutation has an impact on circadian period, shortening it, overexpression of both BBX19 and BBX18 lengthens the circadian period. Interestingly, both BBX18 and BBX19 interact with themselves, with each other and with PRRs (9, 7 and 5). This interaction with PRRs seems to occur in a phased manner, coinciding with the peak expression phases of the PRRs. Next, the authors focus on BBX19 and show that it seems to impinge on a common set of target genes as the PRRs, particularly repressing morning phased clock genes. Finally, the authors propose that the PRRs are required for the recruitment of BBX19 to the CCA1 promoter, although I do not agree with this last statement as I find it not well supported by the data (and also not well depicted in the model in Figure 9).

Authors' Response

Thank you for your pertinent comments and suggestions on the data. For this reason, we have revised the description of results of the full text to tone down the claims of PRRs in the binding of BBX19 to the target gene promoter. After discussion, we give a relatively suitable description of the working model.

Little is known about regulators of the transcriptional activity of clock proteins, and hence the reported findings are interesting to the field. I find however that some statements (e. g. regarding the requirement of PRRs) should be backed up by more data or toned down. Another major concern is the language, as in many instances the text is difficult to understand and leads to confusion. The manuscript would undoubtedly benefit from English proofreading.

Authors' Response

Your comments are critical to improve the readability of our manuscript. We have done careful proofreading of the full text, especially the presentation of the results cannot be overstated.

After carefully reading the manuscript, I encourage the authors to consider the following comments.
- Line 112, Figure 1 D, the mean period length of CCA1:LUC oscillations in bbx19-3 mutants was determined to be 23.6 h but the figure shows a mean period of 23.5 h. Please revise it. Also, regarding statistical analyses, in the figure legend it is stated that "at least 24 individual seedlings were used" but supplemental Table S2 shows a maximum of 24. Additionally, an ANOVA should have been performed instead of a t-test.

Authors' Response

Thank you for the revised comments.

We checked the raw data and revised the period length of *prr19-3* to 23.5 ± 0.1 h. The sample size was at least 15 individual plants. The analysis of multiple groups was performed with one-way ANOVA followed by Tukey's multiple comparison test, P < 0.05, and the statistical differences were labeled.

- Line 114: "evolutionary analyses in multiple species suggested the roles for B-box domains at their N-terminus in DNA binding and transcriptional regulation". I wonder, how do authors deduce this from supplemental figure 2? The analyses rather show that the N-terminus is conserved.

Authors' Response

We are sorry that the content written here has caused you confusion. This sentence is indeed beyond the scope of evolutionary analysis. We have deleted inappropriate inferences.

- Line 117: "...indicating that they play a vital role in the maintenance of the circadian clock". Authors should tone down the message here. The BBX are required to keep the pace of the clock, but are not vital, as the clock still runs in the bbx mutants.

Authors' Response

Thank you for the suggestions.

We have deleted this overstated word. In the revised manuscript, we just point out more pertinently that BBX19 may play a certain role in regulating the circadian clock.

- Line 123, I would rather just say that BBX19 and 18 are involved in adjusting the pace of the clock and are rhythmically expressed in the day. BBX18:BBX18 and BBX19:BBX19 lines lengthen the pace of the clock. One would assume that both proteins are still rhythmically expressed (because their expression is driven by an endogenous promoter fragment), though they likely reach higher levels than the WT, therefore slowing down the clock. So, it is not only the rhythmic expression of these BBX proteins what is important for period length, but also their level.

Authors' Response

We appreciate your kind guidance. We accept your analysis and have revised this sentence.

-Figure 2 legend would also benefit from some revision, better stating what information refers to which panel or panels.

Authors' Response

Thank you for the revised comments. We have revised Figure 2 legend.

- Figure 3A, I see weak interaction with ELF3 and maybe also for TOC1 in yeast. Did the authors investigate these possible associations any further (through BiFC or any other assay)?

Authors' Response

You raised a good question. For this reason, we used firefly LCA (Luciferase Complementation Assay) to analyze the protein-protein interactions between BBX19 and TOC1, ELF3 proteins (see supplemental Figure S6). According to the recombinant LUC activity in transgenic Arabidopsis seedlings, BBX19 has weak interaction with TOC1 and ELF3, respectively. We described this phenotype in the main text and attached Figure S6.

- Figure 3B, please provide negative controls for BiFC assays in which each fusion protein is co-expressed with the empty vector, or ideally with the other half of YFP fused to an unrelated protein.

Authors' Response

Thank you for the revised comments. We uploaded the negative controls of BiFC assays, which was attached to the resubmitted manuscript as supplemental Figure S4.

- Line 137, I guess authors mean here that the expression of the fusion proteins is driven by their own promoters.

Authors' Response

We apologize that the description in this part is not clear enough and has caused you confusion. We have revised the manuscript and the legend in Figure 4A to highlight that the expressed fusion proteins were driven by their own promoters.

- Figure 4, it would be nice to see a negative control, given that, depending on the combination (nLUC or cLUC fused to the N or C terminus), the split luciferase assay is prone to giving non-specific signal. Nonetheless, I would assume that the BBX19-cLUC/PRR9-delPR-nLUC, where no LUC activity (as consequence of apparent lack of interaction) is detected, serves as negative control for this assay (please, include also the combination with BBX18-cLUC). In this regard, for panel D it would be nice to add a rescaled version (maybe added to supplementary figures) where the dynamics of LUC activity, if any, from the BBX19-cLUC/PRR9-delPR-nLUC F1 seedlings can be observed. Also, to serve as true negative control, the lack of interaction between BBX19 (and BBX18) with PRR9-delPR should be tested by other means. Why do panels A, B and C show "normalized LUC activity" and D "LUC activity"? How was it normalized? This happens in other figures, too, where some panels display normalized values and others do not.

Authors' Response

Thank you for the comments.

In the resubmitted manuscript, we uploaded the data of the controls to the supplemental Figure S5.

In Figure 4A-C, we mainly analyze the time specificity of the dynamic formation of BBX19-PRRs protein complex, so the "normalized LUC activity" was used.

In Figure 4D, we analyzed the interaction strength between BBX19 and PRR9 protein with different domain deletions, therefore, the recombined "LUC activity" was used.

In addition, the raw bioluminescence counts of recombined LUC in Figure S5 were provided to indicate the temporal relations of the BBX19-PRRs complex shown in the LCA (Figure 4B-C).

- Line 141, Figure 4A, isn´t the pattern of interaction more like the BBX18 homodimer?

Authors' Response

Thank you for reminding this typo. We have corrected this.

- Line 144, "Collectively, our findings suggested that BBX19 and BBX18 likely act as partners of sequentially expressed PRR9, 7, 5 to temporally regulate the expression of their target genes." I am not sure whether anything on the effect of BBX18/29 on PRR target gene expression can be stated at this point of the manuscript.

Authors' Response

We accept your opinion. We have revised the inference in this paragraph to make it more reasonable. So far, the results just show that the interaction between BBX19 and each PRRs proteins is sequential in a 24-h day.

- Line 150, nothing is shown with regard to BBX protein recruitment to the chromatin, only important domains for protein-protein interactions. Also, as stated earlier, for Figure 4D it would be nice to add a rescaled version where the dynamics of the BBX19-cLUC/PRR9-delPR-nLUC interaction, or the lack of, can be observed.

Authors' Response

We accept your opinion. This sentence is revised.

We have completed the analysis of the dynamic interaction between BBX19 and PRR9 proteins with different domain deletions (see supplemental Figure S5D). We adjusted the Y-axis value range separately and added the control. The results showed that there is no interaction between BBX19-cLUC and PRR9-

delPR-nLUC. In addition, we completed the yeast two-hybrid analysis, confirming that the interaction between BBX19 and PRR9 depends on its PR domain (Figure 4F).

- Line 164, Figure 5, when analyzing genetic interactions, an additive effect is considered as suggesting parallel roles of the investigated genes. That would be the case of BBX19 and PPR5, as well as BBX19 and CCA1, BBX19 and LHY, and BBX19 and TOC1. But the authors interpret these additive effects differently in each case. Could the authors please elaborate on this? Also, looking at the intermediate effects observed for BBX19 and PRR9/PRR7, which might be due to redundancy, it would be interesting to analyze period length in bbx19 prr9 prr7 triple mutants. When combined with clock mutants, bbx19 always shortens the period, but in the case of prr5 prr7 bbx19, which suggests that BBX19 imposes a brake to the pace of the clock. The physical interaction with PRR5, 7, and 9 and the epistasis of prr5,7 over bbx19 suggest that this action takes place through the interaction with these PRRs.

-Line 168, I would rather say that the prr7 prr5 mutation is epistatic over bbx19.

Authors' Response

First of all, we thank you for your analysis of genetic relationships. You have given us a lot of pertinent suggestions for revision. We have rewritten the text, please check the paragraph in the results section about "PRR genes are genetically required for the regulation of BBX19 on the circadian period".

- Line 223, overexpression of BBX19 strongly reduces CCA1:LUC activity. Looking back at Figure 2A, the repressive effect of BBX19 can also be seen (and in Figure 2D, too), but the opposite seems to happen for BBX18, which seems to slightly induce CCA1:LUC (see also the intermediate effect of the double bbx18 bbx19 mutant on CCA1:LUC amplitude). Could the authors please elaborate on the possible different functions of BBX18 and BBX19, even though they both interact with PRRs at the same times (Figure 4)? Figure S4B is also a bit puzzling for me, because such a reduction in TOC1:LUC would be consistent with an increase in CCA1, not a reduction. Finally, regarding the requirement of the PRRs, CCA1:LUC reduction by BBX19 in the prr7/9 and prr5/7 mutants might be less pronounced, but still very significant.

Authors' Response

You raised a good question. The materials in Figure 2A-D were used to analyze the effect of *BBX18* and *19* overexpression on the circadian period length. The *CCA1:LUC* reporter was inserted independently for each genotype, so the influence of *BBX18* or *19* expression on the amplitude of *LUC* expression or *CCA1* promoter activity is not discussed. Since the circadian period of the *bbx18* mutant is consistent with that of the wild type, we focused on exploring the mechanism of *BBX19* involved in the regulation of the circadian clock. We agree with you that the function of BBX18 protein needs to be analyzed. In view of the fact that BBX18 not only interacts with BBX19, but also interacts with PRR9, 7, and 5 sequentially. We speculate that BBX18 may be involved in the regulation of circadian clock as a cofactor of BBX19.

For Figure S4B, the amplitude of *TOC1:LUC* rhythm decreased, but the median value of LUC activity did not change significantly. From another perspective, after overexpression of *BBX19*, the expression of *CCA1* and *LHY*, which are transcription repressors, is down-regulated, while the expression of *RVE8* and *LNK2*, the activators of *TOC1*, may also be down-regulated, leading to a decrease in the *TOC1:LUC* circadian amplitude.

In the resubmitted manuscript, we analyzed the expression of *CCA1*, *LHY*, and *RVE8* for 2 days in LL under *BBX19* overexpression conditions (Figure 7). We observed that the accumulation of *CCA1*, *LHY*, and *RVE8* transcripts began to decline in Col-0 within 12 h after treatment with estradiol. After 12 h, the level of transcripts of each gene was extremely low. This result is similar to the phenotype of *CCA1:LUC* promoter activity inhibited by BBX19 (Figure 8A). In addition, we further analyzed the function of *BBX19* overexpression on morning-phased genes in the *prr7-3 prr9-1* and *prr5-1 7-3* mutants. The results showed that the inhibitory effect of BBX19 on *CCA1*, *LHY*, or *RVE8* expression in the mutant was significantly weaker than that of the wild type (Col-0) (Figure 7). The data demonstrate that PRR9, 7, 5 are required for BBX19 to negatively regulate the expression of *CCA1*, *LHY*, or *RVE8*.

-Figure 8E-F. The interpretation of results in these two panels would benefit from a reorganization of the information provided. I'd suggest the authors to show enrichments in the WT and in the mutant side-by-side for better comparison. ChIP analysis for each gene can be presented as separate panels.

Authors' Response

Thank you for the suggestions on data layout. In the new Figure 8, we have completed the ChIP experiments in the three materials: Col-0, *prr7-3 prr9-1*, and *prr5-1 7-3* mutants (Figure 8E).
Three biological replicates were analyzed for each material.

- Line 237, the "G-box-containing elements" are not necessary, or at least the authors do not show if they are or not. Only thing that can be stated is that BBX19 binds to a region around the G-box. Furthermore, the association to some promoters but not all (there is practically no difference in the association to the CCA1 promoter) is reduced in the absence of PRR7 and PRR9, which again makes the claim about the requirement of PRRs doubtful. Are PRRs required for the association, for the repressive effect of BBX19 or for both?

Authors' Response

Thank you for the revised comments. We accept your suggestion. in the revised manuscript, we modified the text to state the sequence around the G-box.

In addition, we have completed the ChIP experiments in the three materials: Col-0, prr7-3 prr9-1, and prr5-1 7-3 mutants (Figure 8E). The statistical analysis of three biological replicates showed that in the prr7-3 prr9-1 and prr5-1 prr7-3 mutants, the binding of BBX19 to the CCA1, LHY and RVE8 promoter regions did decreased to a certain extent.

In this manuscript, we focus on the BBX19 function as a regulator of circadian pace by complexing with PRR proteins to enhance their repressive effect on CCA1 transcription. Considering the molecular mechanism of PRRs interacting with BBX19, we revised the conclusion of the ChIP assay, and weakened the deductive descriptions.

- Line 241, also line 258, to show that BBX19 recruits PRRs, a ChIP of PRRs in the bbx19 mutant should have been performed. Otherwise, the claim is not supported by the data.

Authors' Response

Thank you for the revised comments. We have revised the discussion of the data, and no longer overstated the recruitment of PRRs by BBX19; instead, we emphasized the interactions between BBX19 and PRRs in BBX19's inhibition of target genes.

TPC2021-RA-00187D 1st Editorial decision – *declined* March 8, 2021

Your resubmission has been evaluated by members of the editorial board, and we regret to inform you that we are not recommending that your manuscript in its current form proceed further in the review process.

We feel it best to not send your paper out for review until you have had sufficient time to complete this work. In the decision letter of the previous version of your manuscript, we stressed that we would only consider a revised version that dealt with the major requests of the reviewers and editors. One of the issues mentioned was "In the editorial assessment, the editors were also concerned that the genetic data were based on analysis of one bbx19 mutant allele with no complementation; the conclusions on the role of BBX19 in regulating period length would need to be confirmed with a second allele or complementation." This point did not seem to be addressed in your response letter. Also, on reading your manuscript again, only bbx19-3 is shown to exhibit the circadian phenotype, and there is no transgenic complementation of the mutation using a BBX19 wild-type transgene. We are still therefore concerned that the manuscript does not demonstrate beyond reasonable doubt that the mutant phenotype shown is caused by the bbx19-3 mutant allele. Other mutations in the background might be responsible for the phenotype as has been described in other

cases in which investigators relied upon a single allele. On this basis, we are not prepared to send this version to the reviewers.

If you can attend to these items we will be happy to reconsider the manuscript, otherwise we recommend submitting to another journal. We thank you for your interest in and support of The Plant Cell. We look forward to your next submission and hope we will be able to render a more positive decision on future work.

TPC2021-RA-00221D Submission received **January 6, 2020**

Thank you for the comments to improve our work. In the resubmitted manuscript, we made detailed revisions to the full text. A total of 10 Figures with new panels (see Figure 4, 5, 7, 8; Figure S2, S5, S6, S7, S9, S10), 3 new table (Table S3, S4, S5) were supplemented. In order to increase the readability of the data, we have replotted Figure 1D, Figure 2, and Figure 6.

In addition, here is a reply to the editor and reviewers' comments point to point:

Editor:

In the editorial assessment, the editors were also concerned that the genetic data were based on analysis of one bbx19 mutant allele with no complementation; the conclusions on the role of BBX19 in regulating period length would need to be confirmed with a second allele or complementation.

Authors' Response

Thank you for the suggestions.

In the resubmitted manuscript, we attached the related data. Circadian rhythms of *CCA1:LUC* (A) or *LHCB1.1:LUC* (B) in *bbx19-1* (SALK_088902), *bbx19-2* (SALK_087493), and *bbx19-3* (SALK_032997) T-DNA insertion individual lines were monitored under free-running conditions (*see* Supplemental Figure S2A-B and Supplemental Table S3). All lines displayed the shortened period of self-sustained circadian rhythms. The genome sequence of *BBX19:BBX19* complemented the circadian phenotype of above *bbx19* T-DNA insertion mutant lines (*see* Supplemental Figure S2C and Table S3).

In addition, the *bbx19-4*, CRISPR/Cas9-mediated genome editing mutation line, was generated and also showed the shortened circadian period length (*see* Supplemental Figure S2D-F and Table S3).

We have resubmitted the manuscript with a new figure S2 and Table S3, primers and method description.

TPC2021-RA-00221D 1st Editorial decision – *revision requested* **April 5, 2021**

Thank you for submitting your best work to The Plant Cell. The editorial board agrees that the work you describe is substantive, falls within the scope of the journal, and may become acceptable for publication, pending revision and potential re-review.

We ask you to pay attention to the following points in preparing your revision:

The reviewers found this version of the manuscript to be much improved and they do not request any additional experiments. However, reviewers 1 and 3 each request several changes to the description of results, explanation of experiments and to the writing. In addition, the description of experiments in two figure legends still do not meet the requirements of The Plant Cell. In Figure 6 panels G to J please make it clear in the legend if the data are based on biological replicates. In the legend to Figure 7 you state that the data shown are based on technical replicates, and that two additional biological replicates were performed but that these are not shown. If the biological replicates cannot be combined in one figure, then we request that at least one biological replicate is shown in the Supplementary Information.

In addition, we agree with reviewer 1 that the writing suffers from some problems with grammar and awkward phrasing, and in general lacks clarity and conciseness. We strongly encourage the use of a professional editing service to ensure

that the work is presented in the best manner and reaches the broadest possible audience. Several editing services have agreed to extend a discount to authors coming to their website via this ASPB page https://aspb.org/aspb-journal-submission-editing-services/.]

-- Reviewer comments:

Reviewer #1 (Comments for the Author):

I appreciate the authors' effort to address the long list of comments made by the 3 reviewers. In my particular case, they have paid attention to most of my previous concerns and many of them were satisfactorily addressed. However, I still think there are issues that require additional attention in order to make the manuscript suitable for publication. Of particular importance is that it still needs a complete revision of English, if possible by a native speaker. Although this version has improved over the previous one, there are still parts that are difficult and cumbersome to read.

Other comments:

-Line 32. I don't see the need to mention the G-box, since authors do not show here whether or not it is necessary for binding to the promoters.

-Line 113. The BBX19 genomic construct has been used to complement bbx19-3, not other bbx19 alleles as stated in the text.

-Line 132. Authors should mention in the text that the Y2H analysis shows weak interaction of the BBXs with ELF3 and TOC1, to be coherent with what they mention in lines 145-148.

-Figure 4E. Although deletion of the EAR motif is not expected to significantly change the size of the fusion protein in the inmunoblot, I'd expect certain change in the mobility of the PRR9-delPR-nLUC protein compared to the full-length version. Is it the whole PR domain what has been deleted? Please, comment on this and add the information to the Methods section. The expected size of fusion proteins should be indicated in the figure legend.

-Lines 176-178. I do not understand why the authors here emphasize that the length of the resulting period in double mutants is equal to the WT. The bbx19 mutation compensates for the long period of mutants ppr7 or prr9, and the duration of the resulting period could be longer or shorter than that of the WT and in no case would the fact that BBX19 slows down the clock be altered.

Reviewer #2 (Comments for the Author):

In this revised manuscript, Yuan et al. successfully responded to this reviewer's previous requests and comments. With the new results, the authors sufficiently demonstrated that BBX19 (and BBX18) have functions in the circadian clock by directly interacting with PRR9, PRR7 and PRR5. I don't have any further suggestions or requests.

Reviewer #3 (Comments for the Author):

This is the resubmitted version of the paper by Yuan et al., which describes genetic and biochemical functions of BBX19 in the circadian clock in Arabidopsis thaliana. In this paper the authors demonstrate that BBX19 directly suppresses transcription of morning-expressed genes such as LHY, CCA1 and RVE8 by physically binding to and cooperating with PRRs, which is significant in maintenance of circadian rhythms in Arabidopsis. In this resubmitted version the authors performed additional experiments to show new data and revised the manuscript according to suggestions in the previous round of review. This present manuscript seems to be significantly improved also fulfilling my requests. I only have several minor comments:

1. As for constructs of PRR9-delPR and PRR9-delEAR, please provide in Material and method detailed information about exactly which peptide regions were deleted. Figure 4E represents bands that may correspond to PRR9-nLuc, PRR9-delPR-nLuc and PRR9-delEAR-nLuc, but these sizes are almost identical despite deletions of amino-acid residues.

2. Please describe the result of ChIP in figure 8E more precisely. cis elements for morning-expressed genes where the binding efficiency of BBX19 is affected by prr mutations look significantly limited. Overall, the argument that PRRs are

required for BBX19 binding to promoters of morning-expressed genes sounds overemphasized. Please tone down the representation within Abstract, Result and Discussion sections.

TPC2021-RA-00221DR1 1st Revision received April 13, 2021

Reviewer comments on previous submission and **author responses**:

Thank you for your rigorous and serious work. Your suggestion helps a lot to improve ourwork. We have carefully completed the revision of the manuscript based on your comments. Please check the following response to the editorial decision, and the list for all changes in the revised manuscript:

Editorial board:

The reviewers found this version of the manuscript to be much improved and they do not requestany additional experiments. However, reviewers 1 and 3 each request several changes to the description of results, explanation of experiments and to the writing.

Authors' Response

We accepted the comments of the editors and reviewers, and continue to make changes in this revised manuscript, including the description of the results, legends, etc. In the second half of thisdocument, we give a list for all changes of the revised content.

The uploaded file also contains tracking revisions, highlighting all the changes, and attaching thereason for the revision in the "Note" column.

In the English language, we invited a plant biologist who is currently engaged in professional English editing work (Genesis Technology Communication (Beijing), Co, Ltd.), Dr. John Hugh Snyder, to make professional changes in English usage, grammar, tense, etc. (attached the signed Language Editing Certification).

In addition, the description of experiments in two figure legends still do not meet the requirementsof The Plant Cell. In Figure 6 panels G to J please make it clear in the legend if the data are basedon biological replicates. In the legend to Figure 7 you state that the data shown are based on technical replicates, and that two additional biological replicates were performed but that these are not shown. If the biological replicates cannot be combined in one figure, then we request thatat least one biological replicate is shown in the Supplementary Information.

Authors' Response

Thank you for the suggestions. In the revised manuscript, we have attached the biological replicate data of Figure 6G-J and Figure 7, as shown in new *Supplemental* Figure S8 and new Figure S9. In addition, in the legend part of Figure 6G-J and Figure 7, the biological replicates related information is described.

In addition, we agree with reviewer 1 that the writing suffers from some problems with grammar and awkward phrasing, and in general lacks clarity and conciseness. We strongly encourage the use of a professional editing service to ensure that the work is presented in the best manner andreaches the broadest possible audience.

Authors' Response

Thank you for the suggestions on the English language. We cooperated with Company *GenesisTech Communication*. They invited Dr. John Hugh Snyder whose native language was English torevise the English expression of the manuscript, and attached the company's proof of revision.

Please note the following:

-The Plant Cell now requires authors to complete and submit an author revisions checklist upon submission of a revised manuscript. The aim of the checklist is to aid authors in preparing a highquality manuscript, facilitate the review and assessment of revised manuscripts, and help to ensure that journal standards are maintained across the board. If your

manuscript is accepted, the completed checklist will be published as supplemental material attached to the article online.Please download a copy of the checklist (pdf fillable form) at this link, for submission with your revised manuscript: https://tpc.msubmit.net/html/Author_Revisions_Checklist.pdf.

Authors' Response

Thank you for the tips on how to prepare high quality manuscript. We have downloaded the relevant PDF and checked them one by one. Please check the uploaded "Authors_Revisions_Checklist" file.

-Supplemental materials should be restricted to large datasets and tables, presentation of replicates, and validation of reagents, methods, or genotypes. Any data that are used to supportthe major claims must be in the main manuscript. Supplemental figure legends must indicate whatfigure in the main manuscript is supported by the supplemental data presented. Please justify how each of the supplemental figures meet the criteria.

Authors' Response

In this revised manuscripts, the supporting figure legends have indicated the association with thefigures in the main text.

-Sampling methods and nature of "biological replicates" should be described precisely (i.e. different plants, parts of plants, pooled tissue, independent pools of tissue, sampled at different times, etc), along with a clear description of and rationale for any statistical analyses conducted.The reader should know exactly what was sampled; what forms the basis of the calculation of anymeans and statistical parameters reported. This is also necessary to ensure that proper statistical analysis was conducted.

Authors' Response

We carefully checked the figure legends, especially the sampling methods, materials, and statistical analysis to ensure the quality of the data.

Please contact us if there are ambiguous comments or if you wish to discuss the revision.

Given the nature of the comments, we are offering you 60 days from when we have issued this decision to complete the revision. If a revision is not returned within this time frame, and if you have not been granted an extension, we will withdraw the manuscript, which will leave you free to submit the work elsewhere. If you need an extension, we encourage you to contact us at any point before the 60 days have passed. If your lab is closed or otherwise compromised due to COVID-19, a further extension of the resubmission deadline may be granted upon request.

When you are ready to submit the revised version, please upload a highlighted copy that indicatesall changes made in response to the editor and reviewer recommendations. Include an itemized list of all changes made in response to each of the reviewer's suggestions in the "Response to Reviewers" section; please note that reviewers do not have access to your cover letter, nor was this decision letter shared with them.

Authors' Response

Thank you for your suggestion. We uploaded an extra revised manuscript with the tracking changes, and added a note to the revised part, corresponding to the comments of the editor andreviewers.

Reviewer #1 (Comments for the Author):

I appreciate the authors' effort to address the long list of comments made by the 3 reviewers. In my particular case, they have paid attention to most of my previous concerns and many of them were satisfactorily addressed. However, I still think there are issues that require additional attention in order to make the manuscript suitable for publication. Of particular importance is thatit still needs a complete revision of English, if possible by a native speaker. Although this versionhas improved over the previous one, there are still parts that are difficult and cumbersome to read.

Authors' Response

We also sincerely appreciate your efforts to revise the manuscript.

This time we cooperated with a professional editing company. They invited Dr. John Hugh Snyder,a professional and native English-speaking editor, to make English language revisions for our manuscript, hoping that this revised manuscript can be more suitable for international readers.

Other comments:

-Line 32. I don't see the need to mention the G-box, since authors do not show here whether or not it is necessary for binding to the promoters.

Authors' Response

Thanks for your suggestion. We accepted your comments and removed the word "G-box" from line 32 of the revised manuscript.

-Line 113. The BBX19 genomic construct has been used to complement bbx19-3, not other bbx19alleles as stated in the text.

Authors' Response

Three T-DNA insertion lines of *bbx19* were used in this study, of which *bbx19-1* and *bbx19-2* werepreviously used and published by colleagues, see Wang et al (2014) Plant Cell 26, 3589-3602. Thus, we did *BBX19* genomic complementary analysis on the unpublished *bbx19-3*. We added the above information to the legend in *Supplemental* Figure S2A-B.

-Line 132. Authors should mention in the text that the Y2H analysis shows weak interaction of theBBXs with ELF3 and TOC1, to be coherent with what they mention in lines 145-148.

Authors' Response

Thanks for your suggestion. We have revised the description on line 132.

-Figure 4E. Although deletion of the EAR motif is not expected to significantly change the size ofthe fusion protein in the inmunoblot, I'd expect certain change in the mobility of the PRR9-delPR-nLUC protein compared to the full-length version. Is it the whole PR domain what has been deleted? Please, comment on this and add the information to the Methods section. The expectedsize of fusion proteins should be indicated in the figure legend.

Authors' Response

Thanks for your suggestion. We have added the following information to the Methods and Figurelegend:

The *PRR9-delPR* construction lacks 118 amino acids (positions 38-156), so PRR9-delPR-nLUC is expected to be 13 kDa smaller than the molecular weight of PRR9-nLUC. The *PRR9-delEAR* construction lacks 20 amino acids (positions 250-269), therefore, PRR9-delEAR-nLUC is expected to be 2 kDa smaller than the molecular weight of PRR9-nLUC. In addition, the molecularweight of the PRR9-nLUC fusion protein is expected to be about 99 kDa; PRR9-delPR-nLUC fusion protein to be about 86 kDa; PRR9-delEAR-nLUC to be about 97 kDa.

We used 10% SDS-PAGE gels to detect PRR proteins migrating. In the biological replicates we completed, the expected protein size of the PRR9-delPR-nLUC and PRR9-delEAR were not much different from that of the PRR9-nLUC. Thus, in a mini-gel system, the mobility distance of the protein potentially does not distinguish the expected certain change in the size of PRR9- delPR-nLUC and PRR9-nLUC proteins.

Lines 176-178. I do not understand why the authors here emphasize that the length of the resulting period in double mutants is equal to the WT. The bbx19 mutation compensates for the long period of mutants ppr7 or prr9, and the duration of the resulting period could be longer or shorter than that of the WT and in no case would the fact that BBX19 slows down the clock be altered.

Authors' Response

We agree with your analysis. In the revised manuscript, this type of comparison has been removed, and the emphasis is on BBX19 slows down the clock.

Reviewer #2 (Comments for the Author):

In this revised manuscript, Yuan et al. successfully responded to this reviewer's previous requestsand comments. With the new results, the authors sufficiently demonstrated that BBX19 (and BBX18) have functions in the circadian clock by directly interacting with PRR9, PRR7 and PRR5.I don't have any further suggestions or requests.

Authors' Response

Your comments help a lot to improve our work. We sincerely thank you for your efforts to revise the manuscript.

Reviewer #3 (Comments for the Author):

This is the resubmitted version of the paper by Yuan et al., which describes genetic and biochemical functions of BBX19 in the circadian clock in Arabidopsis thaliana. In this paper the authors demonstrate that BBX19 directly suppresses transcription of morning-expressed genes such as LHY, CCA1 and RVE8 by physically binding to and cooperating with PRRs, which is significant in maintenance of circadian rhythms in Arabidopsis. In this resubmitted version the authors performed additional experiments to show new data and revised the manuscript according to suggestions in the previous round of review. This present manuscript seems to be significantly improved also fulfilling my requests. I only have several minor comments:

As for constructs of PRR9-delPR and PRR9-delEAR, please provide in Material and method detailed information about exactly which peptide regions were deleted. Figure 4E represents bands that may correspond to PRR9-nLuc, PRR9-delPR-nLuc and PRR9-delEAR-nLuc, but these sizes are almost identical despite deletions of amino-acid residues.

Authors' Response

Thank you for your efforts to improve our manuscript.

We agree with your suggestions. In the revised manuscript, we added the related information ofthe constructs to the Method section, as well as the Figure 4E legend.

We used 10% SDS-PAGE gels to detect PRR proteins migrating. In the biological replicates we completed, the expected protein size of the PRR9-delPR-nLUC and PRR9-delEAR were not much different from that of the PRR9-nLUC. Thus, in a mini-gel system, the mobility distance of the protein potentially does not distinguish the expected certain change in the size of PRR9- delPR-nLUC and PRR9-nLUC proteins.

We have added the following information to the Methods and Figure legend:

The *PRR9-delPR* construction lacks 118 amino acids (positions 38-156), so PRR9-delPR-nLUC is expected to be 13 kDa smaller than the molecular weight of PRR9-nLUC. The *PRR9-delEAR* construction lacks 20 amino acids (positions 250-269), therefore, PRR9-delEAR-nLUC is expected to be 2 kDa smaller than the molecular weight of PRR9-nLUC. In addition, the molecularweight of the PRR9-nLUC fusion protein is expected to be about 99 kDa; PRR9-delPR-nLUC fusion protein to be about 86 kDa; PRR9-delEAR-nLUC to be about 97 kDa.

Please describe the result of ChIP in figure 8E more precisely. cis elements for morning- expressed genes where the binding efficiency of BBX19 is affected by prr mutations look significantly limited. Overall, the argument that PRRs are required for BBX19 binding to promotersof morning-expressed genes sounds overemphasized. Please tone down the representation within Abstract, Result and Discussion sections.

Authors' Response

We agree with your pertinent comments and suggestions on the data. For this reason, we have revised the description of results of the full text to tone down the claims of PRRs in the binding ofBBX19 to the target gene promoter. In the Abstract, we removed the previous wording "PRRs arerequired" (*see* line 32-33); in the Results, we revised it as "PRR9, 7, and 5 are involved in the binding of BBX19 to *CCA1* promoter" (*see* line 234).

List of all changes made in response to each of the reviewer's suggestions:

We uploaded an extra revised manuscript with the tracking changes, and added a note tothe revised part, corresponding to the comments of the editor and reviewers.

-Line 1, 17, 19: In English, the phrase "circadian pace" is not common used in the literature, unless the authors are referring to a "circadian pacemaker" in humans. This study is clearly referencing a circadian clock in higher plant, so some instances of "circadian pace" will be changed to "circadian rhythm".

-Line 34-35: We accepted the suggestion of the first reviewer and removed the word "G-box" from line 34 of the revised manuscript.

-Line 137: Revised in accordance with the reviewer 1's comments: "Authors should mention in the text that the Y2H analysis shows weak interaction of the BBXs with ELF3 and TOC1, to be coherent with what they mention in lines 145-148."

-Line 184-186: We accepted reviewer 1's suggestion, removed this type of comparison, and focuson BBX19 slows down the clock.

-Line 228: We accepted the editor's suggestion. In the revised manuscript, we have attached thebiological replicate data of Figure 6G-J, as shown in new Supplemental Figure S8. In addition, inthe legend part of Figure 6G-J, the biological replicates related information is described.

-Line 236: We accepted the editor's suggestion. In the revised manuscript, we have attached thebiological replicate data of Figure 7, as shown in new Supplemental Figure S9. In addition, in thelegend part of Figure 7, the biological replicates related information is described.

-Line 252: We agreed with reviewer 3's pertinent comments and suggestions on the data. For this reason, we have revised the description of results of the full text to tone down the claims of PRRs in the binding of BBX19 to the target gene promoter. In the Abstract, we removed the previous wording "PRRs are required"; in the Results, we revised it as "PRR9, 7, and 5 are involved in the binding of BBX19 to *CCA1* promoter".

-Line 380-382: We accepted reviewer 1's suggestion and added the construct information.

-Line 458-460: We accepted the editor's suggestion. We have added two new supplemental figures to the revised manuscript, see Figure S8-S9.

**TPC2021-RA-00221DR1 2nd Editorial decision – *acceptance pending***	**April 18, 2021**

We are pleased to inform you that your paper entitled "BBX19 fine-tunes circadian rhythm by interacting with PSEUDO-RESPONSE REGULATOR proteins to facilitate their repressive effect on morning-phased clock genes in Arabidopsis" has been accepted for publication in The Plant Cell, pending a final minor editorial review by journal staff. At this stage, your manuscript will be evaluated by a Science Editor with respect to scientific content presentation, compliance with journal policies, and presentation for a broad readership.

**Final acceptance from Science Editor**	**May 11, 2021**
